# Host transcriptional programs underlying lesion development in contagious bovine pleuropneumonia

**DOI:** 10.3389/fimmu.2026.1844390

**Published:** 2026-10-30

**Authors:** Arlind B. Mara, Angela Makumi, Rosemary Grace Ozyck, Massimo Scacchia, Hezron Wesonga, Mark Ackermann, Noah O. Okumu, Winnie Chebore, Morgan L. Hunte, Jeremy M. Miller, Edan R. Tulman, Steven M. Szczepanek, Elise Schieck, Steven J. Geary

**Affiliations:** 1 The Center of Excellence for Vaccine Research, Department of Pathobiology, University of Connecticut, Storrs, CT, United States; 2 The International Livestock Research Institute, Nairobi, Kenya; 3 Istituto Zooprofilattico Sperimentale dell’Abruzzo e del Molise G. Caporale, Teramo, Italy; 4 Kenya Agricultural and Livestock Research Organization, Nairobi, Kenya; 5 Department of Veterinary Pathology, College of Veterinary Medicine, Iowa State University, Ames, IA, United States

**Keywords:** contagious bovine pleuropneumonia (CBPP), lung, pathology, spatiotemporal, transcriptomics

## Abstract

Contagious bovine pleuropneumonia (CBPP), caused by *Mycoplasma mycoides* subsp. *mycoides (Mmm)*, remains a major burden to cattle health and the agricultural industry. *Mmm* is an atypical bacterial pathogen that appears to lack classical virulence factors that cause direct tissue injury (i.e. toxins), and little is known about the mechanisms driving its pathogenicity. The host immune response is believed to be implicated in CBPP pathology, though the molecular mechanisms underlying lesion initiation, progression and chronicity are poorly defined. Classical pathology describes a continuum of lung lesions starting from early inflammation to more mature necrotic lesions and formation of fibrotic sequestra. However, the host transcriptional response driving this potentially immunopathological progression during *Mmm* infection has never been resolved *in vivo*. Here, we performed lesion-stage-resolved transcriptomic profiling of pathological lung tissue collected from experimentally infected animals and compared to healthy lung tissue collected from unchallenged controls. Differential gene expression and functional enrichment analyses were used to identify biological pathways relevant to *Mmm* infection and pathological lesion formation. Early infection was dominated by interferon-stimulated genes and cytokine-responsive pathways, creating a primarily antiviral-like response environment despite the bacterial etiology. Red hepatization showed strong induction of neutrophil chemoattractants, epithelial remodeling markers, and early matrix-remodeling enzymes. Consolidation, spanning red and grey stages, was enriched for innate immune activation, leukocyte adhesion, extracellular matrix organization, and persistent interferon signaling. Grey hepatization reflected late-stage consolidation with heightened neutrophil effector activity, oxidative and proteolytic injury, and macrophage and fibroblast-linked collagen processing. Necrosis/Sequestra lesions showed reduced inflammatory signaling, robust extracellular matrix organization, adhesion, and morphogenetic pathways consistent with encapsulation and sequestrum formation. Our transcriptomic data presented here indicate that the dynamic continuum of CBPP lung pathology appears to be initiated by interferon-primed myeloid recruitment and amplified by neutrophil-driven injury and macrophage- and fibroblast-mediated matrix remodeling, which should be mechanistically investigated. These data further substantiate the role of dysregulated immunity in the development of disease during *Mmm* infection.

## Introduction

Contagious bovine pleuropneumonia (CBPP) is a severe and economically significant respiratory disease of cattle caused by infection with the atypical bacterial pathogen *Mycoplasma mycoides* subsp. *mycoides* (*Mmm*) (1, 2). Disease presents primarily as an acute fibrinous pneumonia and in chronic cases develops into a fibrotic pleuropneumonia followed by the development of fibrotic pulmonary sequestra and pleural adhesions that significantly impair lung function and animal productivity. CBPP is a highly contagious disease with mortality rates of up to 50% (1). Given its impact on animal health and livestock production, CBPP is on the list of notifiable diseases of the World Organization for Animal Health (WOAH).

While CBPP is a devastating disease*, Mmm*, its etiologic agent, appears to lack classical virulence factors (ex. toxins), and *Mmm* virulence has been primarily attributed to components that primarily play metabolic functions or modulate the immune response (1, 3). Hydrogen peroxide (H_2_O_2_) production as a result of glycerol metabolism has been hypothesized to serve as a primary virulence factor of *Mmm*, as it induces cell damage *in vitro* (1, 4). The capsular polysaccharide galactan is also considered to be a major virulence factor as it has been shown to induce vascular and cytopathic effects after intravenous infection, and to modulate the host immune response (1, 5). Immune-mediated lung injury is also thought to be a major contributor to CBPP pathology (1). Indeed, gross histopathological evaluation of CBPP lung lesions follows the classical progression of an inflammatory response as it progresses from pathogen recognition and initial inflammatory cell recruitment through wound repair and fibrosis. Gross lesions of this progression, prominent in lungs of affected animals, include red hepatization, consolidation, grey hepatization, necrosis, fibrosis, necrosis, and sequestra (1, 6). Despite the recognized role that host immune responses play in the pathology of mycoplasma-induced disease (1), we know little about the immunological pathways activated in the lung during infection with *Mmm.* An understanding of the host responses associated with *Mmm* infection is crucial, as conserved responses can inform on pathogenetic mechanisms useful for development of safe and efficacious intervention strategies.

High-throughput transcriptomics provide an opportunity to profile such host responses. While a handful of studies have conducted transcriptional analyses of whole blood or peripheral blood mononuclear cells (PBMCs) collected from infected animals or following *ex vivo* exposures of PBMCs and lung explants to *M. mycoides* species (7–9), there are no reports examining transcriptional host responses at the local site of respiratory infection and primary disease *in vivo*, especially in a lesion resolved manner. A study analyzing the whole blood transcriptome of cattle infected with *Mmm* using microarrays found that the peripheral transcriptome of infected animals reflected an immunosuppressive phenotype which did not reflect the inflammatory nature of the lung (8), making *in situ* lung transcriptomic evaluation critically important for evaluating host-pathogen interactions for *Mmm* infection. Given that interactions of recruited leukocytes with the resident cell populations at the site of infection are critical for shaping the host response and disease outcome, it is critically important that these interactions are studied within an *in vivo* system. Our group has previously utilized RNAseq to probe *in vivo* host-pathogen interactions of *Mycoplasma gallisepticum* within tracheas of infected chickens (10–12). Here, we utilize the same concept to understand host-pathogen interactions of *Mmm* with the bovine lung in the context of *in vivo* infection and specifically of differential pathologies produced as lung lesions mature. We performed RNAseq on lung tissues with specific pathological features (Healthy-appearing (B_HlthI), Red Hepatization (C_RedH), Consolidation (E_Cons), Grey Hepatization (D_GreyH), and Necrosis/Sequestra (F_NecSeq)) collected from infected animals and compared transcriptional profiles to healthy lung tissue from uninfected animals (A_HlthU) (See **Table 1** for sample metadata). These lesions follow a natural temporal maturation during bacterial pneumonia, and allow us to dissect the temporal dynamics of lesion development at the transcriptional level.

**Table 1:** Sample metadata identifying information on the animal from which the sample was collected, date of collection, *Mmm* presence via culture, PCR, and RNA reads mapping to *Mmm* protein coding ORFs.

SampleID	GroupID	Pathology	Infection status	Animal ID	Mmm culture	Mmm RNA	Mmm PCR	Date of collection
*Tissue_1_S1*	A_HlthU	Healthy Section	Uninfected	BV075	–	–	–	25-Nov-24
*Tissue_2_S2*	A_HlthU	Healthy Section	Uninfected	BV075	–	–	–	25-Nov-24
*Tissue_3_S1*	A_HlthU	Healthy Section	Uninfected	BV084	–	–	–	26-Nov-24
*Tissue_4_S3*	A_HlthU	Healthy Section	Uninfected	BV084	–	–	–	26-Nov-24
*Tissue_5_S4*	A_HlthU	Healthy Section	Uninfected	BV084	–	–	–	26-Nov-24
*Tissue_6_S5*	A_HlthU	Healthy Section	Uninfected	BV090	–	–	–	25-Nov-24
*Tissue_8_S7*	A_HlthU	Healthy Section	Uninfected	BV064	–	–	–	26-Nov-24
*Tissue_9_S2*	A_HlthU	Healthy Section	Uninfected	BV064	–	–	–	26-Nov-24
*Tissue_10_S3*	A_HlthU	Healthy Section	Uninfected	BV075	–	–	–	26-Nov-24
*Tissue_11_S8*	A_HlthU	Healthy Section	Uninfected	BV090	–	–	–	25-Nov-24
*Tissue_12_S4*	A_HlthU	Healthy Section	Uninfected	BV090	–	–	–	25-Nov-24
*Tissue_13_S5*	A_HlthU	Healthy Section	Uninfected	BV084	–	–	–	25-Nov-24
*Tissue_14_S6*	A_HlthU	Healthy Section	Uninfected	BV084	–	–	–	26-Nov-24
*Tissue_15_S7*	B_HlthI	Healthy Section	Infected	BV062	+	+	+	26-Nov-24
*Tissue_16_S8*	B_HlthI	Healthy Section	Infected	BV074	+	+	+	5-Nov-24
*Tissue_17_S9*	B_HlthI	Healthy Section	Infected	BV088	+	–	+	6-Nov-24
*Tissue_18_S10*	B_HlthI	Healthy Section	Infected	BV088	+	+	+	28-Oct-24
*Tissue_19_S11*	B_HlthI	Healthy Section	Infected	BV088	+	–	+	28-Oct-24
*Tissue_20_S12*	B_HlthI	Healthy Section	Infected	BV095	+	+	+	28-Oct-24
*Tissue_21_S13*	B_HlthI	Healthy Section	Infected	BV095	+	+	+	29-Oct-24
*Tissue_22_S14*	B_HlthI	Healthy Section	Infected	BV099	+	+	+	29-Oct-24
*Tissue_23_S15*	C_RedH	Red Hepatization	Infected	BV062	+	+	+	5-Nov-24
*Tissue_24_S16*	C_RedH	Red Hepatization	Infected	BV062	+	+	+	5-Nov-24
*Tissue_25_S17*	C_RedH	Red Hepatization	Infected	BV062	+	+	+	5-Nov-24
*Tissue_26_S18*	C_RedH	Red Hepatization	Infected	BV074	+	+	+	5-Nov-24
*Tissue_27_S19*	C_RedH	Red Hepatization	Infected	BV074	+	+	+	6-Nov-24
*Tissue_28_S20*	C_RedH	Red Hepatization	Infected	BV074	+	+	+	6-Nov-24
*Tissue_29_S21*	C_RedH	Red Hepatization	Infected	BV087	+	+	+	6-Nov-24
*Tissue_30_S22*	C_RedH	Red Hepatization	Infected	BV087	+	+	+	7-Nov-25
*Tissue_31_S23*	C_RedH	Red Hepatization	Infected	BV087	+	+	+	7-Nov-25
*Tissue_32_S24*	C_RedH	Red Hepatization	Infected	BV088	+	+	+	7-Nov-25
*Tissue_33_S25*	C_RedH	Red Hepatization	Infected	BV088	+	+	+	28-Oct-24
*Tissue_34_S26*	C_RedH	Red Hepatization	Infected	BV088	+	–	+	28-Oct-24
*Tissue_35_S27*	C_RedH	Red Hepatization	Infected	BV088	+	+	+	28-Oct-24
*Tissue_36_S9*	C_RedH	Red Hepatization	Infected	BV095	+	–	+	28-Oct-24
*Tissue_37_S28*	C_RedH	Red Hepatization	Infected	BV095	+	+	+	29-Oct-24
*Tissue_38_S29*	C_RedH	Red Hepatization	Infected	BV095	+	+	+	29-Oct-24
*Tissue_39_S30*	C_RedH	Red Hepatization	Infected	BV099	+	+	+	29-Oct-24
*Tissue_40_S10*	C_RedH	Red Hepatization	Infected	BV099	+	+	+	5-Nov-24
*Tissue_41_S1*	C_RedH	Red Hepatization	Infected	BV099	+	+	+	5-Nov-24
*Tissue_42_S2*	D_GreyH	Grey Hepatization	Infected	BV062	+	+	+	5-Nov-24
*Tissue_43_S3*	D_GreyH	Grey Hepatization	Infected	BV074	+	+	+	5-Nov-24
*Tissue_44_S4*	D_GreyH	Grey Hepatization	Infected	BV087	+	+	+	6-Nov-24
*Tissue_45_S5*	D_GreyH	Grey Hepatization	Infected	BV087	+	+	+	7-Nov-25
*Tissue_46_S6*	D_GreyH	Grey Hepatization	Infected	BV087	+	+	+	7-Nov-25
*Tissue_47_S7*	D_GreyH	Grey Hepatization	Infected	BV088	+	+	+ *weak	7-Nov-25
*Tissue_48_S8*	D_GreyH	Grey Hepatization	Infected	BV095	+	+	+	28-Oct-24
*Tissue_49_S9*	D_GreyH	Grey Hepatization	Infected	BV095	+	+	+	29-Oct-24
*Tissue_50_S10*	D_GreyH	Grey Hepatization	Infected	BV095	+	+	+	29-Oct-24
*Tissue_51_S11*	D_GreyH	Grey Hepatization	Infected	BV099	+	+	+	29-Oct-24
*Tissue_52_S12*	D_GreyH	Grey Hepatization	Infected	BV099	+	+	+	5-Nov-24
*Tissue_54_S14*	E_Cons	Consolidation	Infected	BV074	+	+	+	5-Nov-24
*Tissue_55_S15*	E_Cons	Consolidation	Infected	BV088	+	+	+	6-Nov-24
*Tissue_56_S16*	E_Cons	Consolidation	Infected	BV088	+	+	+	28-Oct-24
*Tissue_57_S17*	E_Cons	Consolidation	Infected	BV088	+	+	+	28-Oct-24
*Tissue_58_S18*	E_Cons	Consolidation	Infected	BV088	+	+	+	28-Oct-24
*Tissue_59_S19*	E_Cons	Consolidation	Infected	BV088	+	+	+	28-Oct-24
*Tissue_60_S20*	E_Cons	Consolidation	Infected	BV088	+	+	+	28-Oct-24
*Tissue_61_S21*	E_Cons	Consolidation	Infected	BV088	+	+	+	28-Oct-24
*Tissue_64_S24*	B_HlthI	Healthy Section	Infected	BV065	+	+	+	28-Oct-24
*Tissue_65_S25*	B_HlthI	Healthy Section	Infected	BV069	+	+	+	28-Oct-24
*Tissue_66_S26*	B_HlthI	Healthy Section	Infected	BV069	+	+	+*weak	29-Oct-24
*Tissue_67_S27*	B_HlthI	Healthy Section	Infected	BV069	+	+	–	25-Nov-24
*Tissue_68_S28*	B_HlthI	Healthy Section	Infected	BV072	–	–	–	26-Nov-24
*Tissue_69_S29*	B_HlthI	Healthy Section	Infected	BV072	+	+	+	26-Nov-24
*Tissue_70_S30*	B_HlthI	Healthy Section	Infected	BV091	–	–	+*weak	26-Nov-24
*Tissue_71_S11*	B_HlthI	Healthy Section	Infected	BV065	+	+	+	25-Nov-24
*Tissue_72_S12*	B_HlthI	Healthy Section	Infected	BV091	–	–	–	25-Nov-24
*Tissue_73_S13*	B_HlthI	Healthy Section	Infected	BV091	–	–	–	26-Nov-24
*Tissue_74_S14*	C_RedH	Red Hepatization	Infected	BV072	+	+	+	25-Nov-24
*Tissue_75_S15*	C_RedH	Red Hepatization	Infected	BV072	+	+	+	26-Nov-24
*Tissue_76_S16*	C_RedH	Red Hepatization	Infected	BV091	–	–	–	26-Nov-24
*Tissue_77_S17*	D_GreyH	Grey Hepatization	Infected	BV091	–	–	–	25-Nov-24
*Tissue_78_S18*	F_NecSeq	Necrotic-Sequestra	Infected	BV072	+	+	+	25-Nov-24
*Tissue_79_S19*	F_NecSeq	Necrotic-Sequestra	Infected	BV072	+	+	+	26-Nov-24
*Tissue_80_S20*	F_NecSeq	Necrotic-Sequestra	Infected	BV065	+	+	+	26-Nov-24
*Tissue_81_S21*	F_NecSeq	Necrotic-Sequestra	Infected	BV065	+	+	+	25-Nov-24
*Tissue_82_S22*	F_NecSeq	Necrotic-Sequestra	Infected	BV065	+	+	+	25-Nov-24
*Tissue_83_S23*	F_NecSeq	Necrotic-Sequestra	Infected	BV072	+	+	+*weak	25-Nov-24
*Tissue_84_S24*	F_NecSeq	Necrotic-Sequestra	Infected	BV072	+	+	+	25-Nov-24
*Tissue_85_S25*	F_NecSeq	Necrotic-Sequestra	Infected	BV072	+	+	+	25-Nov-24
*Tissue_86_S26*	F_NecSeq	Necrotic-Sequestra	Infected	BV091	+	+	+	25-Nov-24

## Results

### Nebulization challenge resulted in successful infection and development of disease in all *Mmm* exposed animals

Challenge of animals via nebulization resulted in the successful infection of animals inoculated with Mmm Afade, while animals nebulized with sterile medium did not develop disease. Infected animals began to display clinical disease roughly 2 week after infection, with some meeting humane endpoint criteria as early as 13 days post infection. **Figure 1** indicates the timeline of animal necropsies which were conducted as animals reached humane endpoint criteria, or at the end of the study period. Note that while a survival curve is shown, no animals succumbed to natural infection, but were rather humanely sacrificed once clinical endpoints were met. Viable *Mmm* was cultured out of lung samples collected from infected animals but not uninfected control animals, confirming successful infection (**Table 1**). Furthermore, *Mmm* specific DNA and RNA were detected throughout all lung specimen from infected animals, further confirming that the challenge was successful.

**Figure 1:**
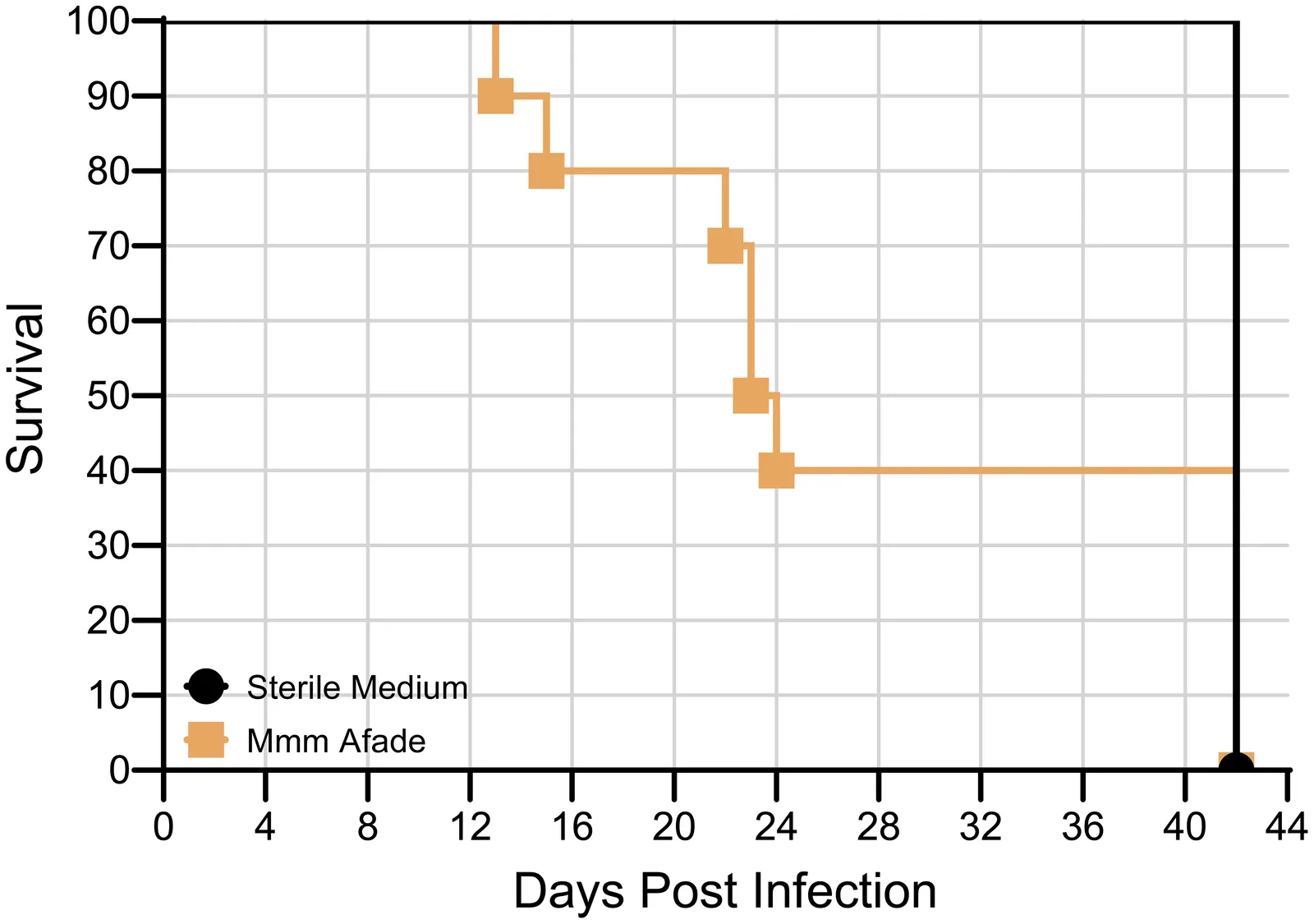
Timeline of animal necropsies in days post infection. Percent survival indicates remaining animals after humane euthanasia and necropsy of animals meeting endpoint criteria.

### Quality control, global sample structure, summary of differentially expressed genes and summary of gene ontology enriched pathways

After processing and alignment to the *Bos taurus* ARS-UCD1.2 genome, the majority of samples (63/81 | 78%) passed QC thresholds with unique alignment >50% to the reference genome. Although, the animals we used in our study are a *Bos indicus* breed, we saw no differences in read mapping rates, and, as the *Bos taurus* genome is better annotated, we moved forward with this alignment. While there are certain studies that indicate that immunological responses to infection differ between the two species (13), these are often due to differential gene expression rather than differences in gene sequences, which are highly conserved. Thus, even though we are mapping to the more well annotated *Bos taurus* genome, the transcriptional responses to infection here represent how *Bos indicus* breeds respond to *Mmm* infection. Sample removal from this QC check resulted in the ultimate analysis of samples with the following lesion breakdown: 5 A_HlthU, 12 B_HlthI, 21 C_RedH, 9 D_GreyH, 8 E_Cons, and 8 F_NecSeq. QC distributions and per condition alignment rates are shown in **Supplementary Figures S1** and **S2** and further sample metadata can be found in **Table 1**. Principal component analysis on variance-stabilized counts revealed a separation of samples by infection status and pathological condition consistent with an evolving lesion landscape, with different lesions forming their own clusters but with some expected overlap apparent between red hepatization, consolidation, and grey hepatization samples (**Supplementary Figure S3**). Notably, the PCA plot indicates that as lesions progressed in maturity, their transcriptional profile became more and more distinct from that of healthy tissue. Sample-to-sample Euclidean distance heatmaps corroborated clustering by condition and infection status (**Supplementary Figure S4**), supporting the validity of downstream differential expression and gene set enrichment analyses. Differential gene expression analysis revealed several thousand genes that were differentially regulated across different types of lesions (**Figure 2**). Compared to Healthy Uninfected tissue, a total of 162 genes were differentially expressed in Healthy Infected tissues (116 upregulated, 46 downregulated); 3160 genes in Red Hepatization (1607 upregulated, 1553 downregulated); 5370 genes in Grey Hepatization (2315 upregulated, 3055 downregulated); 4089 genes in Consolidation (1903 upregulated, 2186 downregulated); and 10,525 genes in Necrotic/Sequestra (7458 upregulated, 3067 downregulated).

**Figure 2:**
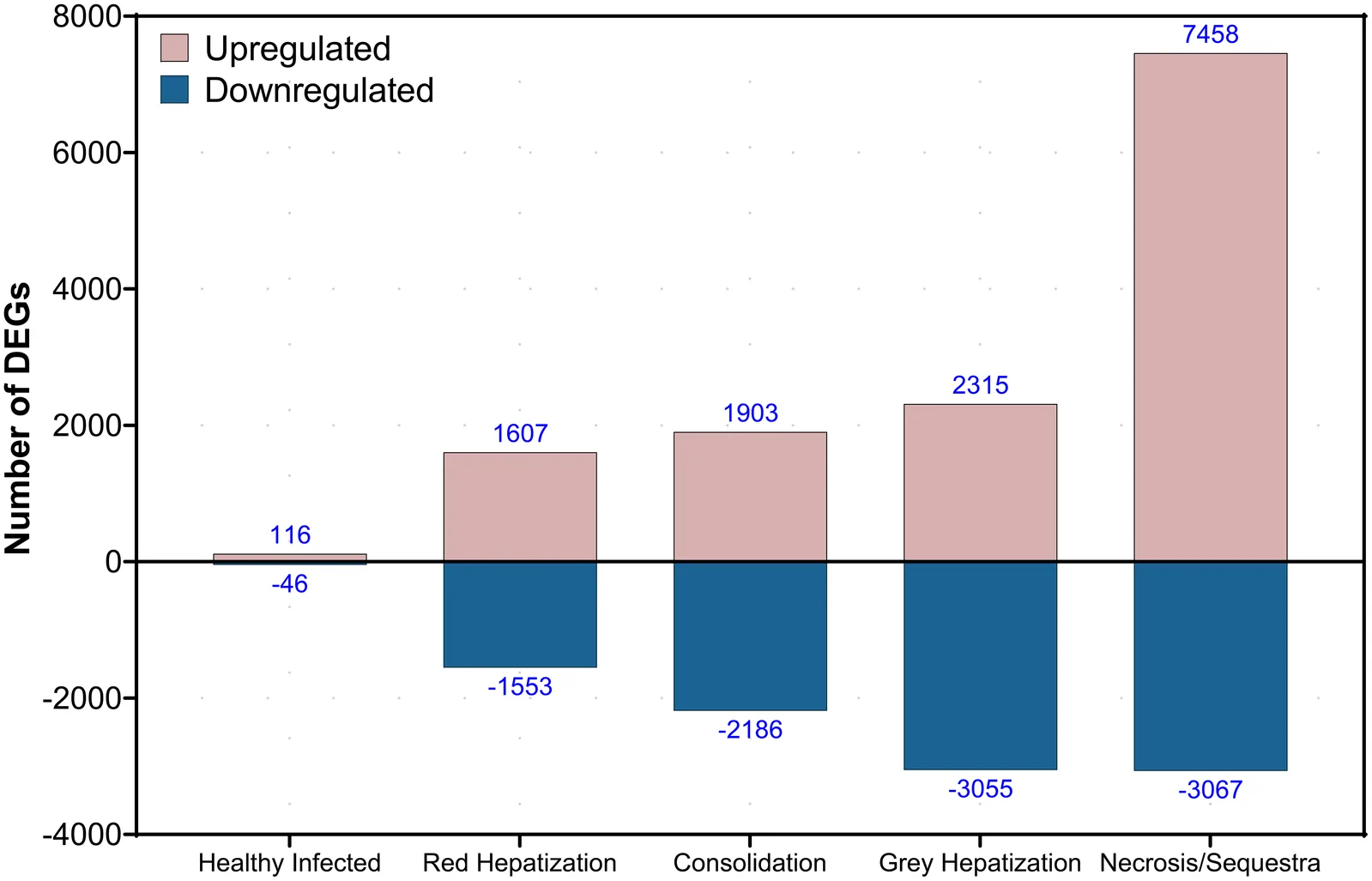
Number of significant differentially expressed genes per pathological condition as compared to healthy uninfected lung tissue.

Across all stage resolved comparisons, gene ontology analysis revealed a clear temporal progression in host biological responses, beginning with an initial dominant interferon mediated antiviral response signature, and culminating in structural remodeling and tissue reorganization (**Table 2**). In early lesions, enriched GO terms were overwhelmingly associated with *defense response to virus*, *response to biotic stimulus* and *regulation of viral processes*, despite the bacterial etiology of CBPP. This appears to be a consistent finding with members of the mycoides cluster, as investigations found *Mycoplasma mycoides* subsp. *capri* also induces an antiviral-like transcriptional profile when interacting with caprine leukocytes (7). As lesions advanced into Red Hepatization, Consolidation, and Grey Hepatization, antiviral signaling persisted but was increasingly accompanied by pathways related to *cytokine responses*, *inflammatory activation*, and *regulation of immune system processes*, consistent with the transition from acute exudation to a more regulated, yet highly active, inflammatory environment. Tissue remodeling pathways were also activated in these lesion stages. In contrast, necrotic tissue and sequestra exhibited a marked shift away from immune dominated signatures toward GO categories associated with *cell adhesion*, *actin cytoskeleton organization*, *cell-cell junction remodeling*, and broader morphogenetic processes. Although residual antiviral activity was observed, they were overshadowed by pathways reflecting fibrosis-driven structural remodeling.

**Table 2:** Summary of top 10 GO terms enriched by pathological condition compared to healthy uninfected control tissues.

Comparison	Description	GeneRatio	p.adjusted
*B_HlthI_vs_A_HlthU*	defense response to virus	21/143	8.33981E-28
*B_HlthI_vs_A_HlthU*	response to virus	21/143	1.1719E-27
*B_HlthI_vs_A_HlthU*	biological process involved in interspecies interaction between organisms	31/143	3.25801E-19
*B_HlthI_vs_A_HlthU*	response to external biotic stimulus	30/143	7.36874E-19
*B_HlthI_vs_A_HlthU*	response to other organism	30/143	7.36874E-19
*B_HlthI_vs_A_HlthU*	response to biotic stimulus	30/143	7.36874E-19
*B_HlthI_vs_A_HlthU*	regulation of viral genome replication	11/143	1.38554E-16
*B_HlthI_vs_A_HlthU*	negative regulation of viral genome replication	11/143	1.38554E-16
*B_HlthI_vs_A_HlthU*	negative regulation of viral process	11/143	2.71627E-16
*B_HlthI_vs_A_HlthU*	viral genome replication	11/143	5.09513E-16
*C_RedH_vs_A_HlthU*	response to virus	41/3787	1.24219E-13
*C_RedH_vs_A_HlthU*	defense response to virus	39/3787	7.06803E-13
*C_RedH_vs_A_HlthU*	regulation of immune system process	147/3787	6.5432E-10
*C_RedH_vs_A_HlthU*	biological process involved in interspecies interaction between organisms	147/3787	1.32117E-09
*C_RedH_vs_A_HlthU*	response to cytokine	79/3787	3.38543E-09
*C_RedH_vs_A_HlthU*	response to peptide	79/3787	3.38543E-09
*C_RedH_vs_A_HlthU*	regulation of defense response	68/3787	3.38543E-09
*C_RedH_vs_A_HlthU*	regulation of response to stress	91/3787	4.96049E-09
*C_RedH_vs_A_HlthU*	inflammatory response	70/3787	1.06552E-08
*C_RedH_vs_A_HlthU*	regulation of response to external stimulus	85/3787	1.06552E-08
*D_GreyH_vs_A_HlthU*	response to virus	41/4157	4.16395E-12
*D_GreyH_vs_A_HlthU*	defense response to virus	39/4157	1.9638E-11
*D_GreyH_vs_A_HlthU*	regulation of immune system process	159/4157	1.19546E-10
*D_GreyH_vs_A_HlthU*	positive regulation of immune system process	132/4157	1.87121E-10
*D_GreyH_vs_A_HlthU*	response to cytokine	84/4157	2.46984E-09
*D_GreyH_vs_A_HlthU*	response to peptide	84/4157	2.46984E-09
*D_GreyH_vs_A_HlthU*	regulation of defense response	72/4157	2.71982E-09
*D_GreyH_vs_A_HlthU*	activation of innate immune response	41/4157	8.96663E-09
*D_GreyH_vs_A_HlthU*	regulation of response to biotic stimulus	52/4157	1.07534E-08
*D_GreyH_vs_A_HlthU*	regulation of response to external stimulus	90/4157	1.31239E-08
*E_Cons_vs_A_HlthU*	response to virus	37/3240	4.06618E-12
*E_Cons_vs_A_HlthU*	defense response to virus	36/3240	4.06618E-12
*E_Cons_vs_A_HlthU*	response to cytokine	74/3240	4.38365E-10
*E_Cons_vs_A_HlthU*	response to peptide	74/3240	4.38365E-10
*E_Cons_vs_A_HlthU*	biological process involved in interspecies interaction between organisms	130/3240	4.53334E-09
*E_Cons_vs_A_HlthU*	cellular response to cytokine stimulus	65/3240	9.32186E-09
*E_Cons_vs_A_HlthU*	inflammatory response	64/3240	9.32186E-09
*E_Cons_vs_A_HlthU*	response to biotic stimulus	125/3240	9.32186E-09
*E_Cons_vs_A_HlthU*	regulation of immune system process	127/3240	1.14222E-08
*E_Cons_vs_A_HlthU*	response to external biotic stimulus	123/3240	2.08336E-08
*F_NecSeq_vs_A_HlthU*	cell adhesion	295/7188	5.84824E-09
*F_NecSeq_vs_A_HlthU*	actin cytoskeleton organization	221/7188	3.35736E-08
*F_NecSeq_vs_A_HlthU*	actin filament-based process	226/7188	8.42582E-08
*F_NecSeq_vs_A_HlthU*	cell-cell adhesion	196/7188	2.56612E-07
*F_NecSeq_vs_A_HlthU*	defense response to virus	43/7188	6.53806E-07
*F_NecSeq_vs_A_HlthU*	neurogenesis	273/7188	6.53806E-07
*F_NecSeq_vs_A_HlthU*	response to virus	44/7188	1.00697E-06
*F_NecSeq_vs_A_HlthU*	anatomical structure formation involved in morphogenesis	103/7188	5.9788E-06
*F_NecSeq_vs_A_HlthU*	generation of neurons	241/7188	2.13814E-05
*F_NecSeq_vs_A_HlthU*	neuron differentiation	227/7188	2.21367E-05

A_HlthU, healthy uninfected; B_HlthI, healthy infected; C_RedH, red hepatization; D_GreyH, grey hepatization; E_Cons, consolidation; F_NecSeq, necrosis/sequestra.

Looking broadly at the top differentially regulated gene signatures across pathological conditions, we see 15 downregulated transcripts and 25 upregulated transcripts that persist throughout the temporal progression of disease as defined by the different pathological states (**Figure 3**). The downregulated signature is dominated by loss of epithelial-basement membrane and tight junction programs. Multiple transcripts associated with structural integrity of the basement membrane were downregulated, including the collagen IV α5 chain *COL4A5*. The laminin γ2 chain *LAMC2*, and the laminin anchor receptor *ITGA3*, which together form the laminin-integrin-collagen axis required for stable epithelial anchoring and basement membrane organization, were also downregulated (14–16). Tight junction and epithelial adhesion molecules such as claudin 7 (*CLDN7)*, the junctional adhesion molecule A (*F11R*/*JAM-A*) and the EpCAM-like tumor-associated calcium signal transducer 2 (*TACSTD2* -*EpCAM/TROP2* family) were also decreased, consistent with impaired barrier function and reduced epithelial cohesion (17, 18).Downregulation of other ECM organizing factors such as the latent-transforming growth factor β binding protein 2 (*LTBP2)*, the laminin related netrin-4 (*NTN4)* and the cytoskeleton-membrane linker chloride intracellular channel 5 (*CLIC5)* further point toward destruction of the epithelial barrier architecture during infection (19–21). Altogether, these changes indicate a coordinated collapse of epithelial barrier and basement membrane integrity during *Mmm* infection.

**Figure 3:**
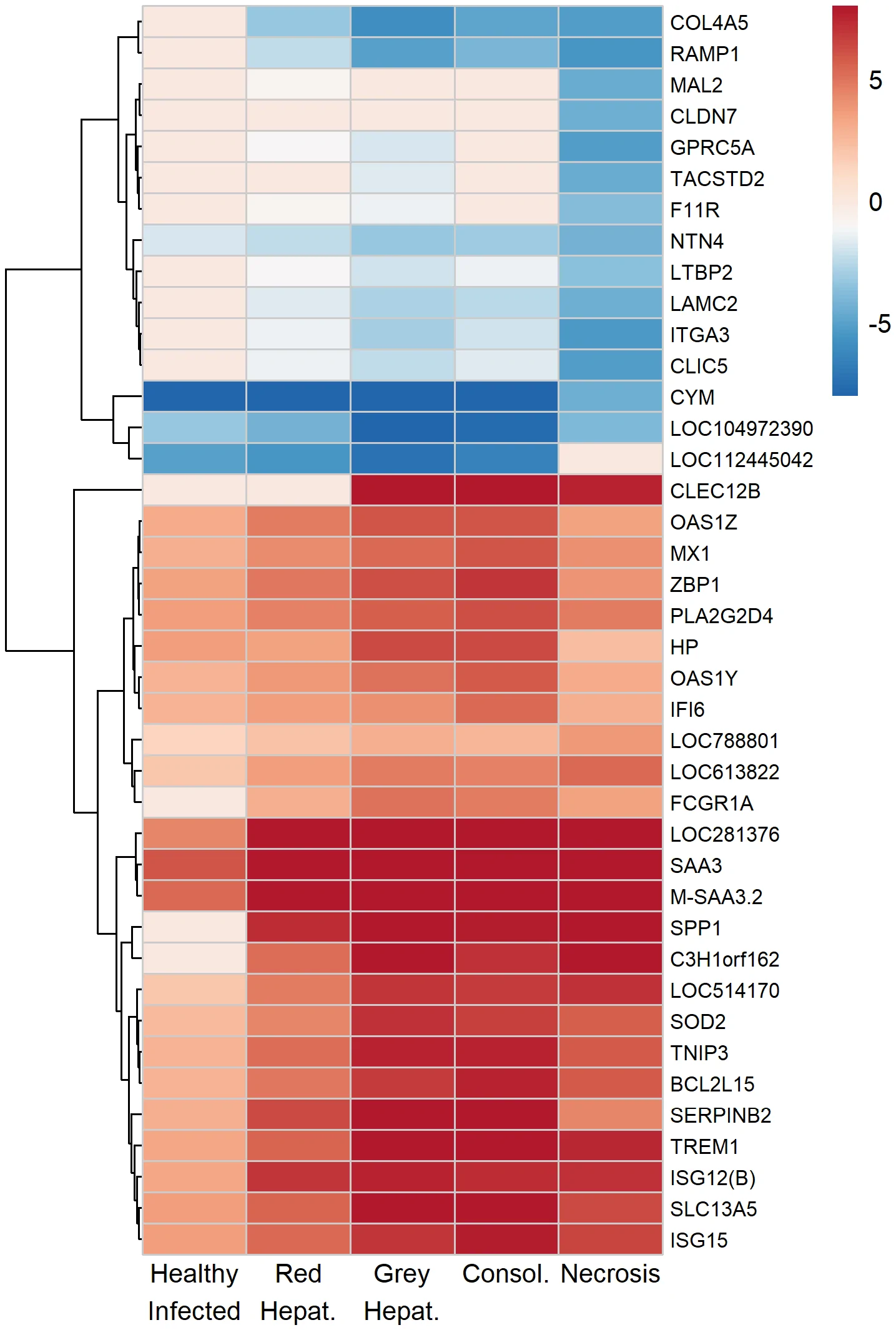
Top differentially expressed genes across pathological conditions. Heatmap reflects log2 fold-change values compared to healthy uninfected tissue from control animals.

Canonical interferon stimulated antiviral effectors such as *MX1, OAS1Z/Y, ZBP1, IFI6, ISG15* and *ISG12(B)* were strongly induced across all infected tissues, consistent with activation of the 2’-5’ oligoadenylate/Rnase L pathway, dynamin-like restriction of viral replication, and cytosolic DNA/Z-DNA sensing (22–25). *ZBP1* sensing of Z-nucleic acid has been shown to result in necroptotic cell death and to heighten the inflammatory response, which may at least partially explain the areas of necrosis observed pathologically in the infected lungs, and the extreme inflammatory environment that is observed at the site of infection. Also upregulated by the infection were acute phase mediators such as haptoglobin (*HP*), serum amyloid A (*SAA3/M-SAA3.2*), and secreted phosphoprotein/osteopontin (*SPP1*) which are known to orchestrate hemoglobin scavenging, neutrophil recruitment, HDL remodeling and Th1-biased macrophage retention at the site of infection (26). Upregulation of the *FCGR1A* (CD64, high affinity Fc-gamma receptor) and the myeloid triggering receptor *TREM1* transcripts further supports the idea that the inflammatory landscape is composed of monocytes/macrophages poised for opsonophagocytosis (27, 28). Collectively, these data indicate that as epithelial barrier integrity programs are suppressed, an interferon-driven antiviral and inflammatory myeloid axis becomes the dominant response. Overwhelming innate responses have been shown to contribute to lung pathology (29), and it is possible that these responses drive the severe and fibrotic lung damage that is observed in infected animals.

Superimposed on this inflammatory environment, several upregulated genes also suggest metabolic and regulatory tuning of the response. The upregulation of the mitochondrial superoxide dismutase *SOD2* is consistent with increased regulation of mitochondrial ROS production in activated cells, while *SLC13A5* upregulation indicates enhanced citrate import to support lipid and energy metabolism during activation (30, 31). At the same time, upregulation of negative regulators such as *TNIP3* (*ABIN-3)* an A20-binding inhibitor of NF-κB, the inhibitory C-type lectin *CLEC12B*, the serine protease inhibitor *SERPINB2* (a.k.a plasminogen activator inhibitor type 2 or PAI-2), and the Bcl-2 family member *BCL2L15* indicates attempts to limit the inflammatory response (32–35). These transcriptional signatures should be further investigated as they may be of utility as biomarkers associated with CBPP disease severity and may inform development of downstream diagnostics.

#### Lesion stage resolved transcriptional profiles: healthy infected versus uninfected

Comparison of healthy lung tissue from uninfected controls and healthy appearing tissue from infected animals provides insights regarding the transcriptional programs that underpin the early host responses to infection. While the healthy infected lung tissue appears normal during gross observation, and shows mainly mild alveolar septal wall thickening when evaluated histologically (**Supplementary Figure S5**), transcriptomic data reveal that this tissue is already engaged in the initial phases of pathogen recognition and response. Surprisingly, despite the bacterial etiology of CBPP, the comparison between healthy infected and healthy uninfected lungs revealed strong enrichment of antiviral and innate immune responses, with the most significantly enriched Gene Ontology categories including GO:0051607 - *defense response to virus*, and GO:0009615 - *response to virus*, driven by a large cluster of interferon-stimulated gene transcripts (ISGs) (**Figure 4**, **Supplementary Table S1**) Processes related to *viral genome replication* and its regulation (GO:0045069, GO:0045071, GO:0019079) were also enriched. Interferon pathways were prominent, with enrichment of *interferon-mediated signaling pathway* (GO:0140888), *type I interferon-mediated signaling pathway* (GO:0060337) and *cellular response to type I interferon* (GO:0071357), underscoring the activation of canonical antiviral defense cascades. Other GO pathways such as *response to external biotic stimulus* (GO:0043207) and *response to other organism* (GO:0051707) reflect recognition of foreign microbial components, indicative of early responses. Further underscoring early responses was the upregulation of lipid mediator and acute phase pathways such as eicosanoid secretion (GO:0032309) and arachidonate transport (GO:1903963).

**Figure 4:**
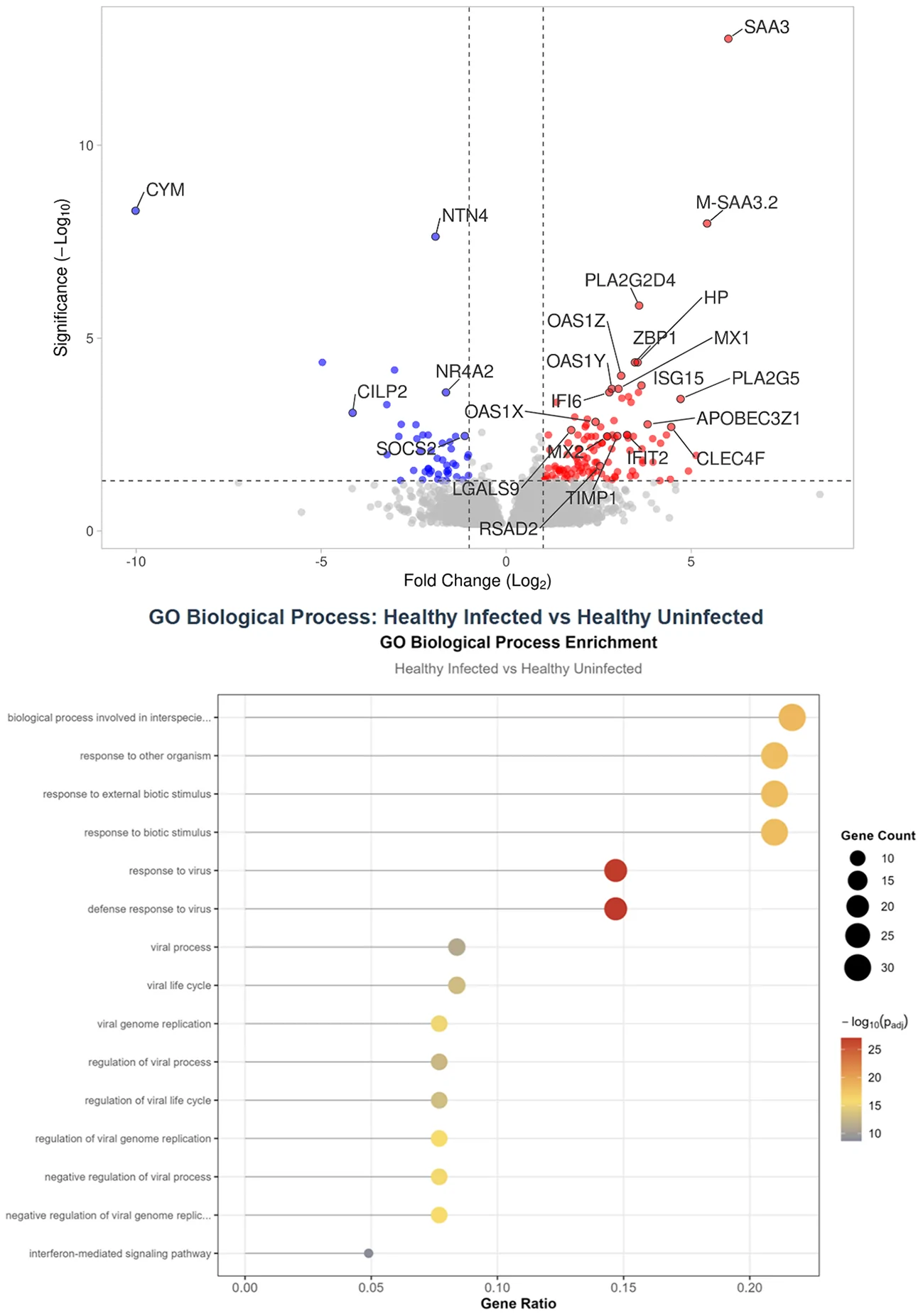
Transcriptional landscape of healthy infected tissues. (Top) Volcano plot highlighting differentially expressed genes with red marking significantly upregulated genes and blue marking significantly downregulated genes. Cut off values defining significant DEGs are a |log2FC|>2 and p-value of less than 0.05 (-log10p of greater than 1.301). (Bottom) Top 15 Enriched GO Terms.

Within these enriched processes, the acute phase protein transcripts *SAA3*, *M-SAA3.2*, and haptoglobin (*HP*) were strongly upregulated, consistent with the rapid phase response to proinflammatory cytokines in bovine epithelia, reinforcing their role as biomarkers of bovine respiratory disease (**Supplementary Table S2**) (26, 36–38). Furthermore, upregulation of phospholipases *PLA2G5* and *PLA2G2D4* is indicative of activation of arachidonic acid metabolite secretion which points to early remodeling of lipid signaling that drive early inflammatory responses through prostaglandin and leukotriene production (39). These factors serve not only as markers of inflammation but also contribute to iron sequestration and neutrophil and macrophage modulation, creating an environment hostile to pathogens but also potentially contributing to immunopathology and bystander injury to lung tissue. The significant upregulation of *ZBP1*, a cytosolic sensor of Z-DNA/RNA that can trigger PANoptosis (pyroptosis, apoptosis, and necroptosis) may indicate that the host immune response contributes to lung pathology during *Mmm* infection (40, 41). A coordinated ISG cluster was evident, including *ISG15*, *MX1*, *MX2*, *OAS1X/Y/Z/Y/X*, *OAS2*, *IFI6*, *RSAD2/viperin*, *IFIT2*, and *APOBEC3Z1* genes which mediate antiviral defense and immune modulation (23, 42–45). *TIMP1*, an inhibitor of matrix metalloproteinases, was upregulated, suggesting early regulation of tissue integrity (46, 47). *CHI3L1*, a chitinase-like protein linked to fibrosis and chronic inflammation was also upregulated, while downregulation of *NTN4* and *CILP2* indicated suppression of structural homeostasis of lung tissue and loss of barrier function (21, 48, 49). Furthermore, *LGALS9* and *CLEC4F* were upregulated while *SOCS2, NR4A2* was downregulated, consistent with enrichment of GO categories for negative regulation of T cell proliferation and negative regulation of lymphocyte activation, which highlights the suppression of the adaptive response during this early phase of innate dominance (50–52).

The upregulation of ISGs aligns well with the enriched GO categories of antiviral defense, confirming that infected but grossly normal lung tissue is already engaged in an antiviral transcriptional reprogramming due to *Mmm* infection, despite the bacterial etiology of CBPP. It is possible that these responses highlight a previously unappreciated intracellular niche for *Mmm* persistence in infected cattle. Importantly, however, type I interferons are also known to suppress adaptive lymphocyte proliferation and promote exhaustion during persistent infections (24). During Mycoplasma infection, these responses may favor pathogen persistence and disease. Strong activation of innate immunity damages lung tissue and remodels the lung environment while also blunting or delaying adaptive immune responses, potentially indicating that *Mmm* may induce these maladaptive immune responses as a way to avoid clearance and promote chronic persistence within the infected lung.

#### Lesion stage resolved transcriptional profiles: uninfected versus red hepatization

Red hepatization represents the first grossly visible stage of CBPP pathology and the next temporally resolved lesion following healthy appearing but infected lung tissue. Acute phase and interferon stimulated transcripts remained elevated and the GO pathways *response to virus* (GO:0009615) and *defense response to virus* (GO:0051607) remained enriched during Red Hepatization, reflecting continued persistence and recognition of *Mmm* infection (**Figure 5**, **Supplementary Table S3**). Other broader immune categories such as *regulation of immune system process* (GO:0002682), *inflammatory response* (GO:0006954), *cell activation* (GO:0001775), and *leukocyte activation* (GO:0045321) were also enriched in Red Hepatization, highlighting expansion of both innate and adaptive immune pathways. Cytokine signaling was prominent, with enrichment of *response to cytokine* (GO:0034097) and *cytokine-mediated signaling pathways* (GO:0019221), underscoring the role of cytokine networks in orchestrating lesion development. Structural remodeling of lung tissue was evidenced through enrichment of *cell adhesion* (GO:0007155), *actin cytoskeleton organization* (GO:0030036), and *cell migration* (GO:0016477), consistent with leukocyte infiltration and tissue breakdown. Importantly, categories such as *positive regulation of immune system process* (GO:0002684) co-occurred with *negative regulation of lymphocyte activation* (GO:0051250), indicating a complex balance of immune stimulation and adaptive suppression that may be actively influenced by *Mmm* infection.

**Figure 5:**
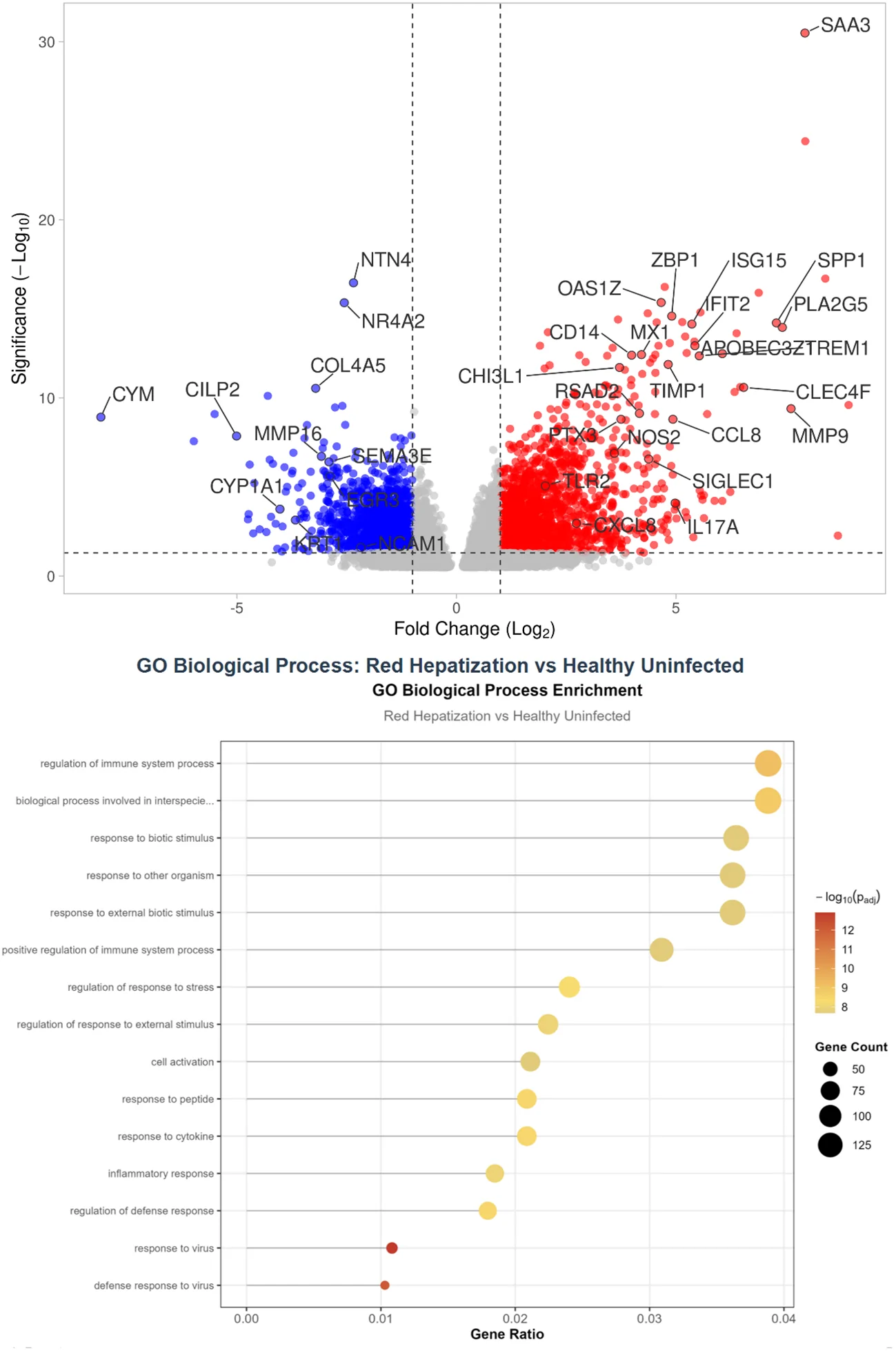
Transcriptional landscape red hepatized tissues. (Top) Volcano plot highlighting differentially expressed genes with red marking significantly upregulated genes and blue marking significantly downregulated genes. Cut off values defining significant DEGs are a |log2FC|>2 and p-value of less than 0.05 (-log10p of greater than 1.301). (Bottom) Top 15 Enriched GO Terms.

Acute phase proteins such as (*SAA3, M-SAA3.2, SAA2, SPP1*), ISGs such as (*ISG15, ISG12(B), MX1, MX2, OAS1X/Y/Z, IFIT2, RSAD2/viperin* and *ISG20***),** lipid mediator signaling factors (phospholipases *PLA2G5, PLA2G2D4*) and other early transcripts such as *ZBP1, APOBEC3Z1*, and *TREM1* that were upregulated in the healthy infected tissue remained upregulated in Red Hepatization, indicating continued persistence and recognition of *Mmm* within the lesion (**Supplementary Table S4**). Inflammatory mediators such as *CCL8*, *CCL19*, *CCL20*, *CXCL2*, *CXCL5*, *CXCL8*, were upregulated, reflecting chemokine driven leukocyte recruitment, primarily of neutrophils, but also monocytes and T cells (53). *IL19, IL17A, IL27* and *IL21* were also upregulated, pointing to cytokine activation of Th17/Th1-associated immune pathways (53).

Matrix remodeling factors such as matrix metalloproteinases *MMP9*, *MMP1*, *MMP13* and *MMP25* were strongly upregulated, while structural and extracellular matrix genes such as collagen *COL4A4*, *COL4A5*, *COL4A6*, *COL15A1*, *COL21A1*, *COL25A1* and *COL2A1* were downregulated, reflecting collagen and basement membrane degradation and suppression of structural homeostasis that retains barrier integrity. *MMP15* and *MMP16* were downregulated, while *TIMP1*, a metalloproteinase inhibitor was upregulated, indicative of selective tissue remodeling (54–56). Chitinase-like protein transcripts *CHI3L1* and *CHI3L2* which are linked to fibrosis (57, 58) were upregulated and can help explain the profibrotic nature of CBPP pathology. Cell adhesion, migration, and synapse formation transcripts such as *ISLR*, *SEMA3E*, *OPCML*, *NCAM1*, *NLGN1* and *KRT1* were downregulated, reflecting epithelial remodeling, and loss of normal lung function (59–64). Transcripts such as *SIGLEC1*, *CLEC4F*, *CD14*, *LBP*, *PTX3*, *NOS2*, *SOD2*, *TLR2*, and *TLR7*, associated with enhanced pathogen recognition, opsonization, reactive oxygen species and neutrophil effector function were upregulated, while metabolic pathway transcripts involved in xenobiotic metabolism such as *CYP1A1, CYP2A13, CYP2C90* and *CYP3A24* were downregulated, indicating loss of normal lung function and a shift toward inflammatory processes (65–72). Despite upregulation of chemokines involved in the recruitment of lymphocytes, several transcription factors linked to adaptive immunity were downregulated (*NR4A1*, *NR4A2*, *NR4A3*, *EGR3*), indicating suppression of T and B cell activation and downstream adaptive immunity (73, 74), which may further support sustained persistence of *Mmm* in infected lungs. Taken together, these transcriptional signatures suggest that Red Hepatization continues to be a stage dominated by amplification of innate responses, immune cell infiltration in the lung parenchyma, continued restrain of adaptive immune responses, and initiation of tissue remodeling. Histopathologic appearance of red hepatized tissue supports the transcriptional landscape describing this stage as one marked by significant leukocyte recruitment and concomitant hemorrhage and edema due to increased vascular permeability (**Supplementary Figure S5**).

#### Lesion stage resolved transcriptional profiles: uninfected versus consolidation

Transcriptionally speaking, Consolidation appeared to represent an intermediate lesion stage between Red and Grey Hepatization, where the acute inflammatory landscape begins to coexist with early adaptive immune activation and structural remodeling. *Pathogen recognition* (GO:0002221, GO:0032496, GO:0002237), *antiviral* (GO:0009615, GO:0051607, GO:0045069, GO:0045071, GO:1903900) and *cytokine/interferon mediated transcriptional signatures* (GO:0034097 GO:0140888, GO:0060337, GO:0071357) that were apparent in the earlier lesions remained enriched in consolidated lesions, indicating continued persistence and recognition of *Mmm (***Figure 6**, **Supplementary Table S5***)*. A pronounced ISG transcriptional signature remained evident, with strong upregulation of genes such as *ISG12(B), ISG15, ISG20, IFIT2, IFIT3, IFIT5,IFI6, IFI16, IFI44, IFI44L, OAS1X/Y/Z, OAS2, MX1, MX2. RSAD2, USP18, XAF1, CMPK2, and EPSTI1* (**Supplementary Table S6**) (45, 75). Transcripts for pattern recognition receptors such as *TLR2, TLR7, NOD1, NOD2, IFIH1 (MDA5), CGAS, AIM2, NLRP1/2/3/12, ZBP1, DHX58 and NLRC4* (76, 77) were upregulated and were accompanied by high-expression of myeloid-associated receptors such as *SIGLEC1, CLEC4D/E/F, CLEC12A/B, CLEC10A and CLEC5A*, consistent with enhanced pathogen recognition and phagocytosis (78, 79). Chemokines and neutrophil-associated transcripts were also highly expressed, including recruiting factors such as *CXCL2, CXCL3, CXCL5, CXCL8, CXCR1, CXCR2* and activity factors such as *S100A8, S100A9, S100A12, LCN2, OLFM4, NCF1/4 and CYBB*, indicating active neutrophil recruitment and effector function (80–82).

**Figure 6:**
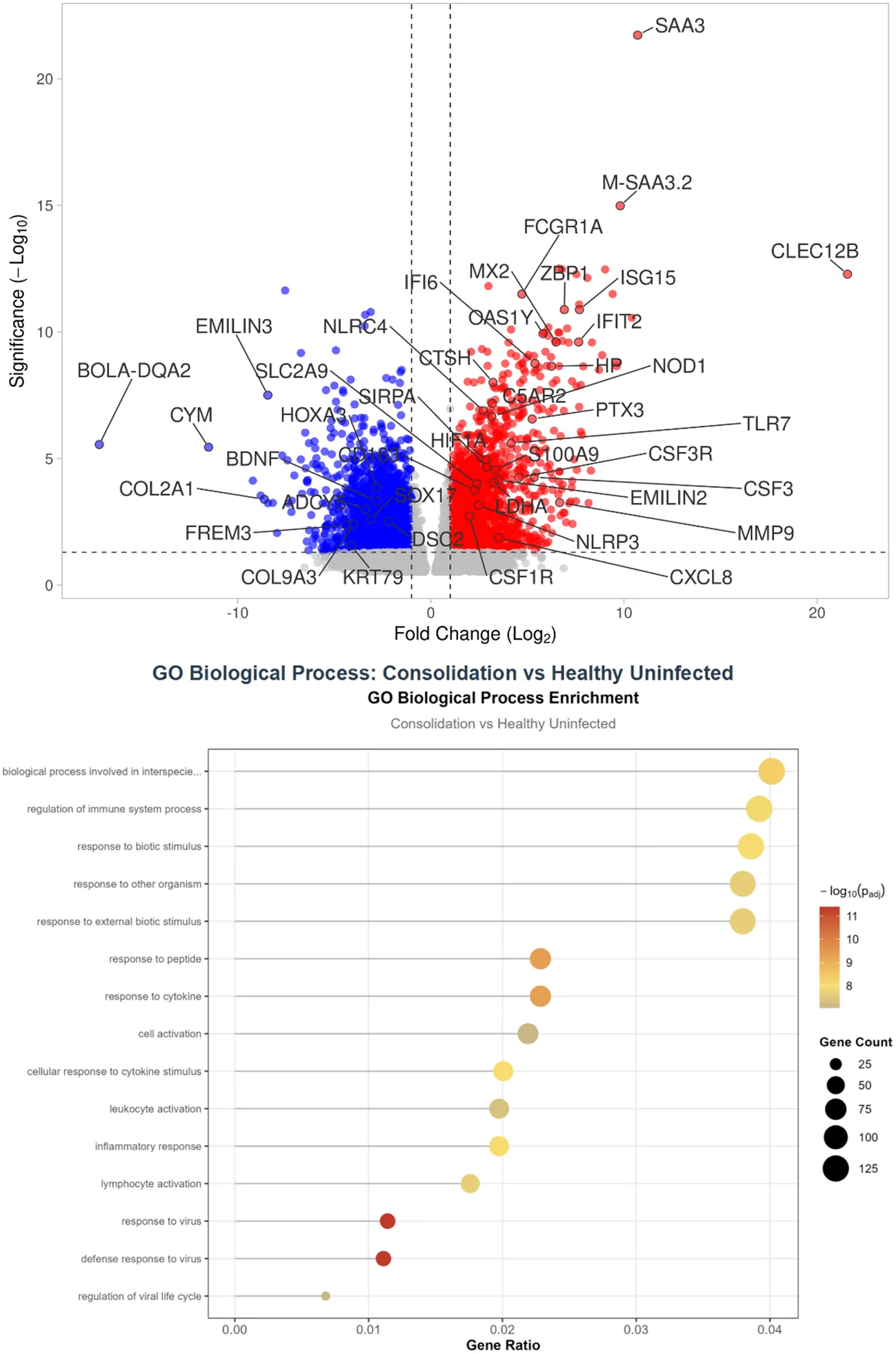
Transcriptional landscape of consolidated tissues. (Top) Volcano plot highlighting differentially expressed genes with red marking significantly upregulated genes and blue marking significantly downregulated genes. Cut off values defining significant DEGs are a |log2FC|>2 and p-value of less than 0.05 (-log10p of greater than 1.301). (Bottom) Top 15 Enriched GO Terms.

In addition to these inflammatory cues, pathways associated with cell adhesion and early structural remodeling were also enriched in Consolidation lesions (GO:0098609 – *cell-cell adhesion*, GO:0007159 – *leukocyte cell-cell adhesion*, GO:0030198 – *extracellular matrix organization*, and GO:0045229 – *external encapsulating structure organization*), indicating the beginning of the development of characteristic fibrotic lesions and sequestra that are observed with CBPP. Macrophage activation markers (*CD68, CD163, CSF1R, CSF3R, CSF3, FCGR1A, FCGR2B, SIRPA, SIRPB1/2, TREM1 and TREM2*) (83–85) and acute-phase/complement genes (*SAA2, SAA3, HP, HPX, LBP, PTX3, C2, C5AR1, C5AR2*) (26, 69, 86) were upregulated, consistent with heightened phagocytic and complement activity. The increase in macrophage associated transcripts also signals the initiation of profibrotic processes and structural remodeling, which was further reflected by the upregulation of *MMP1/9/25, TIMP1, CTSL, CTSK, CTSS, VCAN, MXRA5, EMILIN2, PLOD1*, and *P4HA1*, indicating loss of lung architectural integrity (56, 87, 88). Hypoxia-linked metabolic reprogramming was also indicated by upregulated *HIF1A, EGLN3, BNIP3, LDHA, HK2, SLC2A1* and *SLC2A3*, further highlighting loss of normal lung function (89–91). This was further supported by loss of transcripts associated with epithelial cell identity, cilia and airway function, and structural components of healthy lung tissue such as *SFTPC, AQP5, AGER, BPIFA2A, COL2A1, COL9A1/2/3. COL4A2/3/4/5/6, HAPLN1, FREM2, FREM3, PCDH20, DSC2, KRT79* (92–94). Many neuronal associated transcripts (*STMN2, GRIN2B, NTRK2, BDNF, ADCY5*) and developmental regulators (*SOX7/17. FOXO4/6, HOXA3/5*) were also downregulated (95–97). Together, these transcriptional signatures indicate that Consolidation represents a stage characterized by intense innate immune activation, interferon signaling, neutrophil-driven inflammation and damage, macrophage recruitment, and progressing extracellular matrix and lung architecture remodeling that result in lung pathology and loss of normal organ function. Supportive of the landscape described by the transcriptional profile of the lesion, histopathological evaluation of consolidated lung tissue revealed significant leukocyte recruitment into the conducting airways and alveolar spaces, necrosis of bronchial epithelia, as well as characteristic perivascular and peribronchial lymphocytic aggregation indicative of hyperplasia of Bronchus Associated Lymphoid Tissue that is often observed during respiratory Mycoplasma infections (**Supplementary Figure S5**).

#### Lesion stage resolved transcriptional profiles: uninfected versus grey hepatization

Gene set enrichment analysis of Grey Hepatization revealed a transcriptional landscape that still retains the strong innate immune imprint seen in Red Hepatization and Consolidation, evidenced by the persistent enrichment of *response to virus* (GO:0009615), defense response to virus (GO:0051607), *pattern-recognition receptor signaling* (GO:0002221), and *interferon mediated signaling* (GO:0140888, GO:0060337), which is expected given the chronic nature of *Mmm* infection (**Figure 7**, **Supplementary Table S7**). Unlike Consolidation, however, the transcriptional profile of Grey Hepatization shows a shift toward immune regulation and resolution, with pathways related to *negative regulation of immune system process* (GO:0002683) and *negative regulation of signaling* (GO:0023057) being highly enriched. Concurrently, Grey Hepatization displays a stronger emphasis on tissue remodeling and structural reorganization, reflected in the enrichment of pathways relating to *extracellular matrix organization* (GO:0030198), *extracellular structure organization* (GO:0043062), *external encapsulating structure organization* (GO:0045229) and *actin cytoskeleton organization* (GO:0030036). The enrichment of these pathways marks the transition from the exudative, neutrophil-rich environment of Red Hepatization and Consolidation, toward the macrophage and fibroblast driven restructuring the defines the fibrotic nature of CBPP pathology. In addition, pathways related to *angiogenesis* (GO:0001525), b*lood vessel morphogenesis* (GO:0048514) and *circulatory system development* (GO:0072359) are far more prominent in Grey Hepatization than in Consolidation, consistent with the vascular remodeling that may result in granulation tissue formation following this stage.

**Figure 7:**
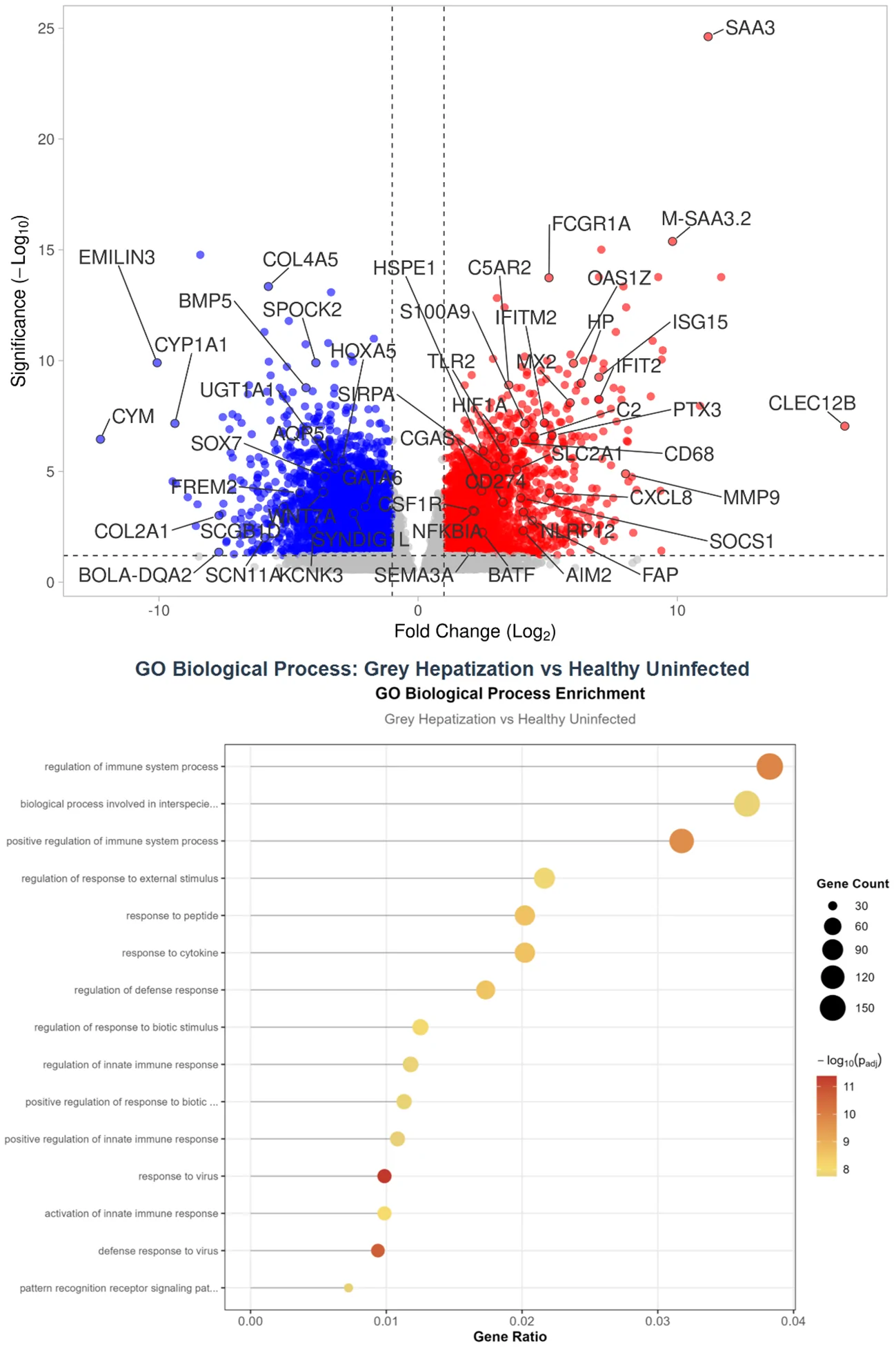
Transcriptional landscape of grey hepatized tissues. (Top) Volcano plot highlighting differentially xpressed genes with red marking significantly upregulated genes and blue marking significantly downregulated genes. Cut off values defining significant DEGs are a |log2FC|>2 and p-value of less than 0.05 (-log10p of greater than 1.301). (Bottom) Top 15 Enriched GO Terms.

At the gene level, Grey Hepatization lesions exhibited transcriptional programs that retained many of the innate immune features observed in previous stages but with a clear shift toward immune modulation, transition to a macrophage and fibroblast dominated environment and structural remodeling of lung tissue (**Supplementary Table S8**). Upregulated pattern-recognition receptors (*TLR2, TLR7, IFIH1, CGAS, AIM2, ZBP1, DHX58* and *NLRP12*) and ISGs (*ISG15, ISG12, IFIT2, IFIT3, IFITM1/2/10, MX1, MX2, OAS1X/Y/Z, OAS2, RSAD2, USP18, XAF1, CMPK2*, and *EPSTI1*) indicate that pathogen sensing and interferon mediated pathways remain active, indicative of persistent infection despite an overwhelming inflammatory response (43–45, 77). However, the concurrent upregulation of regulatory mediators including *SOCS1/3, TNFAIP3, TNIP1/3, NFKBIA, DDIT4* and *IKBKE* suggest that the inflammatory pathways are increasingly counterbalanced by negative feedback circuits characteristic of resolution of inflammatory responses (98–102). However neutrophil-associated transcripts (*CXCL2, CXCL3, CXCL5, CXCL8, CXCR1, CXCR2, S100A8, S100A9, S100A12, LCN2, OLFM4, NCF1/4* and *CYBB)* remained elevated, indicating that neutrophil activity persists into this mature pathological lesion (80–82). Macrophage activation markers such as *CD68, CD163, TREM1, TREM2, CSF1R, CSF3R, FCGR1A, FCGR2A/B, SIRPA, SIRPB1, TYROBP* and *OSMR* are strongly upregulated, reflecting the transition toward a macrophage-rich environment that orchestrates tissue remodeling and clearance of debris (83–85). Acute phase and complement opsonization transcripts (*SAA2, SAA3, HP, HPX, LBP, PTX3, C2, C9, C5AR1* and *C5AR2*) remained elevated, indicative of ongoing complement activation and phagocytic clearance (26, 69, 86).

Extracellular matrix (ECM) remodeling and stromal activation pathways were prominent in Grey Hepatization, with strong upregulation of *MMP1, MMP9, MMP13, MMP14, MMP25, CTSL, CTSK CTSH, CTSZ, TIMP1, VCAN, MXRA5, EMILIN2, CTHRC1, PLOD1, PLOD2, P4HA1*, COL-related regulators (87–89), and fibroblast associated transcripts such as *FAP*, *TGFBI*, *SULF1*, and *MEDAG* (103–106). These changes align with the enrichment for GO pathways for *extracellular matrix organization*, *extracellular structure organization*, and *external encapsulating structure organization*, marking the transition toward dense, airless, structurally reorganized fibrotic tissue that is characteristic of this lesion stage, and eventually leads to encapsulated sequestra.

Vascular remodeling signatures were also prominent. Upregulated angiogenesis-associated transcripts included *SEMA3A, SEMA4D, PROK2, FGF23, IL11, IL21R, GDF15, GUCY2D, VCAM1, NID2, THBS2, CREB3L1, RASA4B* and *ETV3L*, which indicates neovascular remodeling as the lung transitions from exudative inflammation toward structural reorganization and fibrotic remodeling that impairs normal lung function and is characteristic of CBPP pathology (107–111). Consistent with these changes, metabolic and hypoxia-associated genes such as *HIF1A, EGLN33, BNIP3, LDHA, HK2, ENO1, ALDOC, SLC2A1* and *SLC2A3* were upregulated, indicating persistent metabolic reprogramming within hypoxic damaged lung tissue (112–115). Additional stress-response and survival genes such as *DDIT3 (CHOP)*, *GDF10*, *SESN2*, *HSPH1*, *HSPA1A* and *HSPE1* further indicate that the lung microenvironment is undergoing damage from oxidative stress, nutrient deprivation and cellular turnover (116–119).

Grey hepatization was also characterized by a sweeping downregulation of transcripts associated with alveolar epithelial identity, surfactant biology, metabolic specialization, structural integrity, and neuro-epithelial signaling, which reflected profound architectural collapse and extreme loss of functional integrity of affected lung tissue. Downregulation of transcripts such as *SFTPC, AQP5, SCNN1B, CLDN18, CLDN5, AGER, SLC34A2, SLC22A17, SLC22A31, SLC9A4, SLC5A9, SLC16A11*, and *SLC45A1* indicates loss of pneumocyte maintenance programs and destruction or dedifferentiation of epithelial cells and replacement of alveoli with dense proteinaceous and fibrinous scar tissue (120–126). Downregulation of *BPIFA2A, SCGB1D, SCGB2A2, MUC4*, and *SPINK5* further supports loss of secretory and barrier forming epithelial cells (127–131). Downregulation of components that mediate maintenance of extracellular matrix and basement membrane architecture such as *COL2A1, COL4A1/3/4/5, COL6A6, COL9A1/2/3, COL11A2, COL13A1, COL15A1, COL21A1, COL24A1, COL25A1, COL26A1, COL27A1, COL5A3, COLQ, FREM1/2/3, HMCN1/2, SPOCK1/2, WFDC1, WFIKKN2, PRELP, EMID1, EMCN* and *MXRA7* further reflect degradation of the alveolar scaffold and replacement with disorganized fibrotic matrix (132–138), a hallmark of CBPP pathology.

A suppression of developmental and morphogenetic regulators also indicates the collapse of epithelial-mesenchymal signaling networks that maintain tissue architecture with transcripts such as *SOX4/7/13/17, HOXA2/3/5, HOXB3/5/6, TBX1/2/3/4, TCF21, PRDM6, PRDM16, GATA3/5/6, IRX1/2/3/5, NKD1/2, AXIN2, WNT2/7A/8B/9A/11, BMP5/6, BMPER, FGF1/12/20, FGFR1/2/3/4* and *NOTUM* being downregulated in grey hepatized tissue (138–148). Downregulation extended into neuronal, neuroendocrine, and synaptic signaling pathways with decreased expression of *STMN2, GAP43, NTM, GRIN1/2B/3A, CHRNB3/4, CNR1/2, ADCY5, ADCYAP1R1, PDYN, CRHR2, GALR3, GPR37/55/ASP1/ASP2, SYT7/8/13/25B, RIMS1/3, SCGN* and *SYNDIG1L* (95).

Further supporting loss of lung function is the downregulation of transcripts encoding factors involved in surfactant metabolism, xenobiotic detoxification, lipid handling, with *UGT1A6/2A1*, *HPGD*, *ABCC6*, *ABCB1*, *CYP1A1/1A2/2A13/2C87/2C90/4A22/4B1/17A1*, *FM01/5, GSTA1/2/3, GSTT2* and *GSTM3*, which reflect the collapse of epithelial metabolic capacity and the inability of damaged alveoli to maintain surfactant composition, lipid turnover, or oxidative homeostasis (72). Furthermore, downregulation of ion channels, transporters, and signaling receptors (*SCN11A/3B, KCNK3/B2/D3/H2*, *CNGA2*, *TRPV4/M4/C3*, *P2RX1/5*, *ASIC2/3*, *AQP1/5, SLC16A9/5A9/24A3/6A1/8A3,22A31*) reflects the functional shutdown of epithelial transport capacity and the loss of barrier physiology (149–156).

In the immune axis, numerous immune modulatory and lymphocyte associated transcripts were differentially regulated. Transcripts like *IL7R, IL23R, CCR1, CCR2*, *CCR5*. *CXCL10*, *LAG3*, *CTLA4*, *CD69*, *CD274* (PD-L1), *BATF, SPI1, POU2F2*, and *PRDM1* reflect a shift toward adaptive immune engagement and regulation, aligning with the enriched GO pathways for T cell activation, B cell activation, lymphocyte proliferation and regulation of lymphocyte activation (53, 157–164). This indicates that adaptive immunity becomes more prominent during Grey Hepatization as innate neutrophilic responses begin to resolve. Downregulation of genes associated with antigen presentation such as *BOLA-DQA2*/*DQA5*, *CD1A/B* indicates suppressed cycling of antigen presentation machinery which supports adaptive immune activation (165–167).

Taken together, the transcriptional profile of Grey Hepatization reveals a lung environment undergoing epithelial collapse, loss of specialized metabolic and structural integrity programs, loss of neuronal and developmental processes, and dismantling of normal extracellular matrix architecture, underscoring a transition toward a structurally reorganized, fibrinous, and functionally impaired tissue state. Histopathological appearance of grey hepatized tissue is marked by apparent fibrosis of interlobular septa, edema, and marked necrosis of cells that make up the lung parenchyma (**Supplementary Figure S5**), supporting the transcriptomic observations.

#### Lesion stage resolved transcriptional profiles: uninfected versus necrosis

Necrotic tissue from fibrotic enclosed sequestra represents the most mature, chronic lesion produced by *Mmm* infection. A large proportion of the most strongly upregulated transcripts in this lesion corresponded to unannotated or partially annotated loci (*LOC*-). Because limited functional annotation exists for these transcripts, their biological role in the context of *Mmm* infection is difficult to interpret. However, their high fold-change values and consistent enrichment among the top differentially expressed genes may indicate that substantial components of the sequestrum-associated transcriptional response remain uncharacterized at the gene level. As such, these loci may represent important targets for future annotation and functional investigation, especially in the context of chronic tissue remodeling and immune modulation.

Pathway analysis on annotated DEGs from necrotic tissue displayed a transcriptional profile enriched by *cell adhesion*, *actin cytoskeleton organization* and *cell-cell junction pathways* (GO:0007155, GO:0030036, GO:0098609), consistent with the formation of dense, fibrotic capsules that physically isolate necrotic lung tissue (i.e. Sequestra) (**Figure 8**, **Supplementary Table S9**). Upregulated transcripts included multiple profibrotic and matrix modifying enzymes (*MMP1/3/9/13/12/17/19/25*) (54–56) as well as adhesion molecules (*CDH9/18, CDHR2, PCDH8/15, CNTN2* and *CNTNAP2)* (168, 169) (**Supplementary Table S10**). Enrichment of morphogenetic processes such as *anatomical structure formation*, *tube morphogenesis*, *vasculature development*, and *regulation of anatomical structure morphogenesis* (GO: 0048646, GO:0035239, GO001944, GO:0022603) is indicative for activation of fibroblast driven tissue remodeling programs that culminate in sequestrum development, and reorganize peripheral architecture. This was further supported by enrichment of *cell morphogenesis*, *cell projection organization*, and *actin filament dynamics* (GO:0000902, GO:0031344, GO:0030041/42), indicating ongoing cytoskeletal reorganization within fibroblasts, endothelial cells and residual leukocytes as the lesion matures. Transcription factors such as *HOXA10/1,1 HOXC8/9, HOXD10/13, PAX1/2/3/6/8, POU3F1/2/3/, DLX1/3/4/5*, and *BARX1* were upregulated alongside development-linked genes (*WNT6/9B/10A, RSPO4, BMP1, GDF15*) (140, 145, 146), indicating that certain repair processes that may retain tissue function are also induced. Pathways related to *angiogenesis* and *blood vessel morphogenesis* (GO:0001525, GO:0048514) further support the development of new vasculature at the sequestrum periphery where granulation tissue persists, further indicating an active effort to restore normal function. This is evidenced by transcripts such as *ANGPTL6*, *VEGF*-responsive channels (*KCNK9, KCNH4*), *ECM1*, *COL22A1, COL19A1, COL23A1*, and *MXRA5* (87–89, 150) being upregulated. Newly evident in these mature lesions was the upregulation of numerous transcripts associated with cell cycle progression, DNA replication, and cell proliferation (*CCNB1, CCNB2, CDC20, CDC6, MCM10, BIRC5, AURKA/B, PLK1, KIF20A/18B*), which indicates ongoing turnover of stromal and immune cells within the lesion associated with healing and repair processes exemplified by the enrichment of *regulation of multicellular organismal processes* (GO: 0051239) and *regulation of developmental growth* (GO:0048638) (170, 171).

**Figure 8:**
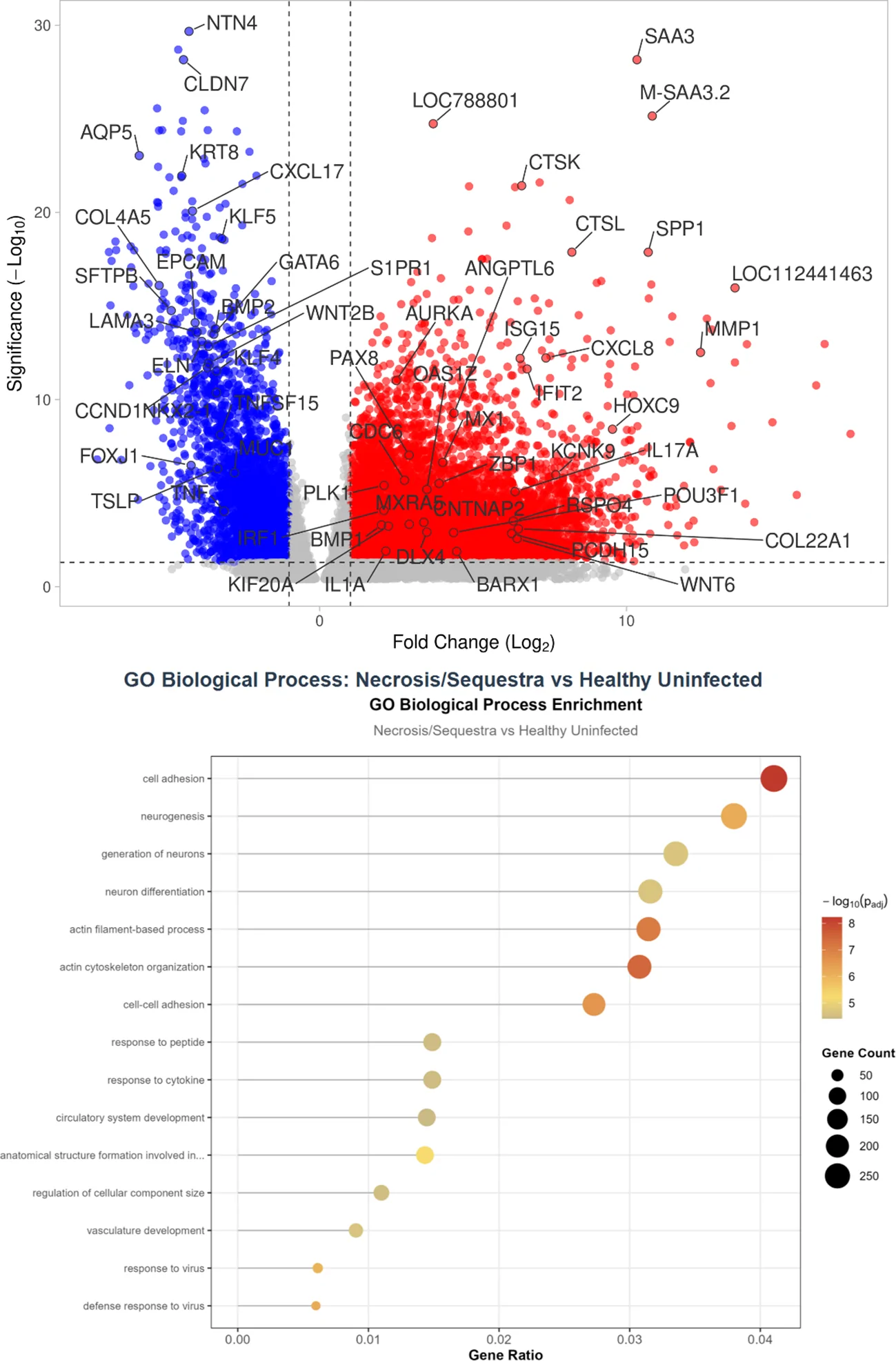
Transcriptional landscape of sequestra and necrotic tissues. (Top) Volcano plot highlighting differentially expressed genes with red marking significantly upregulated genes and blue marking significantly downregulated genes. Cut off values defining significant DEGs are a |log2FC|>2 and p-value of less than 0.05 (-log10p of greater than 1.301). (Bottom) Top 15 Enriched GO Terms.

On the other hand, downregulated transcripts indicated a profound loss of epithelial cell identity, with transcripts encoding hallmark airway and alveolar structural components such as surfactants, secretoglobins, keratins, epithelial adhesion factors (*SFTPA1/B/C/D, SCGB1A1/B1D/B3A2/B2A2,AQP5 EPCAM, KRT7/8/18/19, AGER, S1PR1*) and tight-junction constituents (*CLDN1/3/4/5/7/18* and *OCLN*) being reduced compared to healthy tissue controls (120–126). Downregulation of *NKX2-1, FOXA1, GATA6* and *HOPX*, and epithelial cell renewal transcripts *CCND1*, *KLF4/5*, *TEAD1/3/4* and *YAP1* further indicates collapse of the transcriptional network that maintains lung epithelial lineage identity (92–94). This was further supported by downregulation in developmental genes involved in Wnt, BMP and Notch signaling (*DKK2/3, WNT2B/7A/11, BMP2/3/4/6, NOTCH3* and *JAG2*) (146, 148). Genes associated with ciliary assembly and motility were downregulated, including *FOXJ1, DNAH5/11, DNAL1/4, CFAP43/47/52, CFAP299, IFT57*, and *TEKT1/3* (172). The downregulation of *MUC1/15* and *PIGR* further support the collapse of the mucosal barrier function (130). Multiple collagen IV isoforms (*COL4A1/2/3/4/5/6*), laminins (*LAMA3/5, LAMB2/3*), laminin related netrin (*NTN4*) and other matrix associated proteins (*ELN, FBLN5, HMCN1, PRELP, SPON2, ITIH5, CLIC5*) were downregulated, indicating degradation of the epithelial basement membrane and consistent with the necrotic appearance of the tissue (55, 87–89, 132). This was accompanied by the downregulation of angiogenic and endothelial genes *CD34*, *CDH5*, *TEK*, *TIE1*, *ESAM*, *RASIP1*, *GJA5*, *PLVAP*, *AQP1* and *MMRN2*, which indicates loss of microvascular endothelium within necrotic cores and aligns with the avascular nature of sequestra and the replacement of lung tissue with fibrotic encapsulation (173). Furthermore, metabolic and transport pathways were also broadly suppressed with xenobiotic and lipid-metabolizing enzymes (CYP1A1/2A13/2B6/2F1/4B1, AOX1/2. FMO2/3/5, UGT1A1/1A6, UGT2A1), and solute carriers (SLC6A14/6A20/16A11/22A31/45A1) being downregulated (72, 126). Furthermore, several transcripts relating to epithelial-driven immune response (*CXCL17, IL17RB, IL20RB, CD83, CD209, KLRK1, TNFSF15, TNF, ILDR1/2* and *TSLP*) were downregulated, reflecting the collapse of epithelial derived cytokine networks that is likely resultant from epithelial cell death (53, 92).

Surprisingly, despite the obliterated normal tissue architecture in necrotic lesions, we observed persistent, though attenuated, *interferon-mediated signaling* and *response to virus pathways* (GO:0140888, GO:0009615), suggesting persistence of low level innate immune activation without the robust inflammatory response seen in earlier stages. ISG transcripts such as *ISG15, IFIT2/3, IFI6/30, IFIH1, OAS1X/Y/Z, OAS2, RSAD2, MX1, MX2, USP18, ZBP1, IRF1/4/7* and *IFI44/44L* were upregulated, indicating a persistent interferon imprinting (22–25). Chemokine and cytokine related genes such as *CXCL2/5/8, CCL3/8*, *IL1A/B*, *IL22/24/26/17A/17F/21/21R/23R/7R*) were also upregulated (53). This indirectly indicates that *Mmm* continues to persist in late-stage lesions, and has the ability to survive the overwhelming inflammatory host response. Importantly, we are able to culture viable *Mmm* out of all lesion types, and detect Mmm DNA and RNA in these lesions via PCR and RNAseq read mapping (**Table 1**), which demonstrates the ability of Mmm to survive the inflammatory landscape it encounters in the host, and continue to persist even in heavily damaged lung tissue. Other immune related pathways indicated largely regulatory processes, rather than effector-driven, with *negative regulation of viral processes*, *negative regulation of cytokine responses*, and *modulation of leukocyte proliferation and activation* (GO: 0048525, GO0032101, GO:0045321)GO terms being enriched.

Collectively, the transcriptional profile mirrors the pathological phenotype of necrotic tissue and depicts these lesions as terminally remodeled, non-viable tissue compartments in which epithelial identity, barrier integrity, ciliary and metabolic function have collapsed, while fibrotic structural remodeling and persistent interferon imprinting dominate the surviving cellular landscape. The significant suppression of airway and alveolar functional programs (such as surfactant production, mucociliary clearance, tight junction architecture), underscores the complete loss of functional parenchyma that is visually apparent in the affected lung tissue. Concurrently, the induction of matrix-modifying enzymes, angiogenic modulators, and morphogenesis-linked transcripts reflects a microenvironment focused on encapsulation, containment, and long-term stabilization of necrotic tissue rather than regeneration. This polarized transcriptional state supports sequestra as chronic, structurally isolated lesions maintained by stromal and immune programs that prioritize physical isolation of damaged/necrotic tissue over eventual restoration of normal lung architecture. These observations were mirrored in histopathological evaluation of necrotic tissue which displayed overt areas of necrotic epithelial cell debris, fibrin deposition, and hints of organizing pneumonia and granulation tissue (**Supplementary Figure S5**).

### Cell-type deconvolution analysis supports a coordinated shift from structural integrity to innate, adaptive, and chronic inflammatory programs across CBPP stages

Differential gene expression and gene ontology analysis above indicate that CBPP lung lesions result from an evolving inflammatory landscape, however traditional bulk RNA sequencing cannot distinguish whether these transcriptional programs arise from transcriptional programming within stable cell populations or from changes in cellular composition. To resolve this ambiguity, and assess whether the inferred biological processes correspond to shifts in the underlying cellular landscape, we performed a z-score enrichment based cell-type deconvolution using curated cell specific gene signatures obtained from the Cattle Cell Atlas (CattleCA) (174). Cell type deconvolution is a computational technique that aims to infer the composition of cell populations within a heterogenous tissue using bulk RNAseq data (175). By refining cell specific transcripts using single cell RNAseq data from a bovine host, we hoped that this approach would allow for adequate estimation of the evolving cellular landscape in CBPP lung lesions.

Our analysis revealed a stage-dependent reorganization of the cellular landscape during CBPP progression that was supportive of the inferences made from differential gene expression and gene ontology analysis above, and supported by the histopathological appearance of the lesions. Healthy uninfected lungs were dominated by epithelial and endothelial signatures, consistent with intact alveolar and vascular architecture (**Figure 9**; **Supplementary Figure S6**). These signatures declined steadily as the lesions progressed toward the Necrotic/Sequestra lesion stage, further indicating cell death and loss of functional and structural integrity of the lung due to progressing infection. As the lesion progressed toward Red Hepatization, we see enrichment of signatures associated with myeloid cells such as neutrophils and monocytes/macrophages consistent with recruitment of innate effectors that are characteristic of this pathological stage. Much like previous analysis indicated, Grey Hepatization was dominated by macrophage and fibroblast signatures, indicating profibrotic processes that culminate in the replacement of damaged lung tissue into fibrinous scar tissue that is a hallmark of CBPP lesions. Lastly, an enrichment of T cell and B cell signatures in late-stage lesions is indicative of the formation of germinal centers within developing tertiary lymphoid structures in the lung, which are a prominent histopathological characteristic of chronic Mycoplasma infection. The ongoing enrichment of neutrophil signatures throughout lesion maturation further indicates continued pathogen persistence in infected lungs, indicating the host response is not productive, and is unable to clear *Mmm* infection, and our ability to detect Mmm DNA and RNA, as well as culture viable *Mmm* out of these lesions, heavily indicates that the immunological host responses mounted against *Mmm* infection are not protective (**Table 1**).

**Figure 9:**
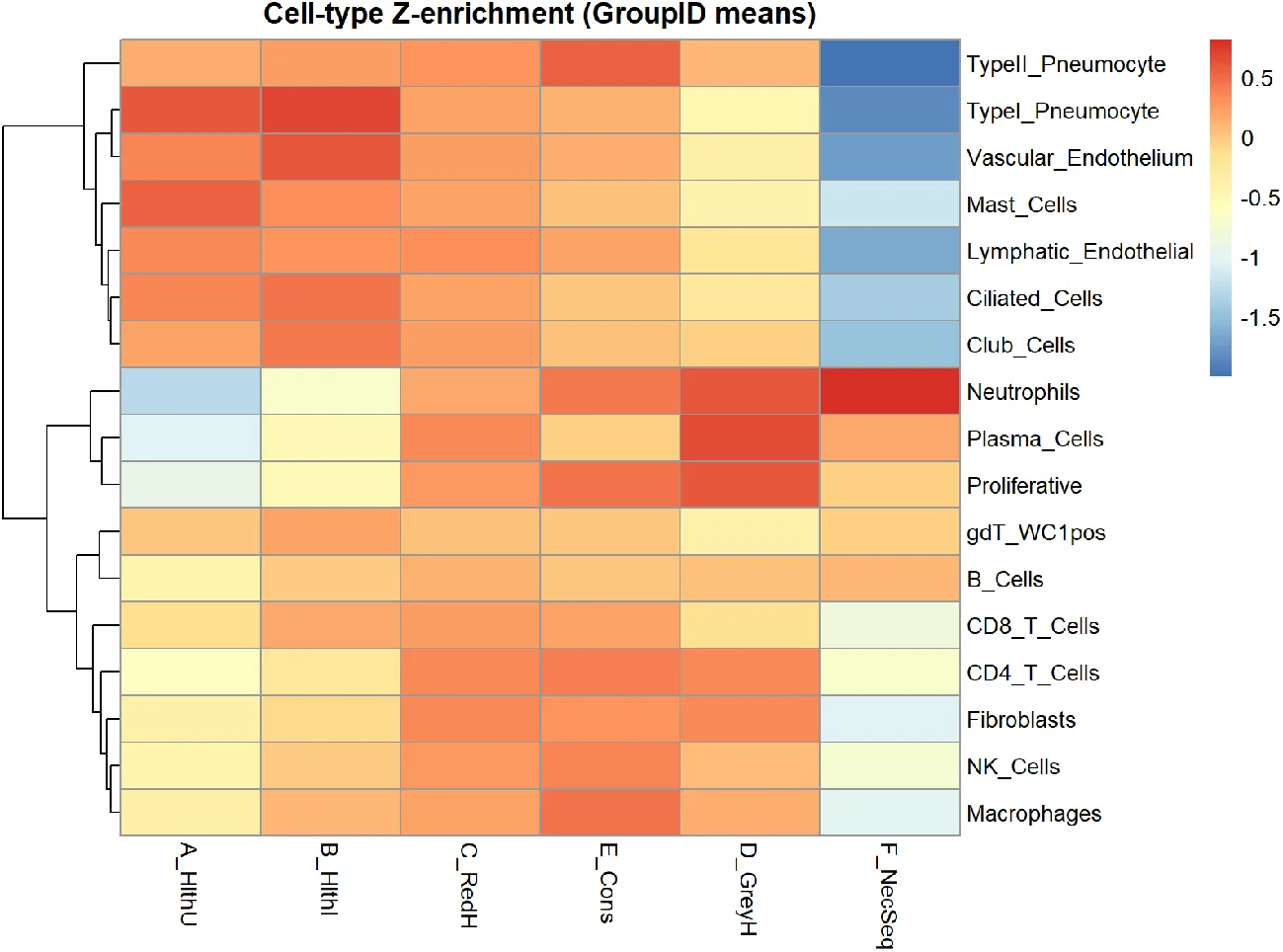
Z-score signature enrichment across CBPP lesion stages. The heatmap shows Z-score normalized enrichment values for curated bovine lung cell-type gene signatures (Macrophages, B cells, Neutrophils, Plasma cells, Dendritic Cells, Fibroblasts, T cells/ILCs, NK cells, Endothelial cells , Epithelial cells, etc).

## Discussion

Current literature indicates that the bovine response to *Mycoplasma mycoides* subsp. *mycoides* (*Mmm*) is heavily anatomically compartmentalized. CBPP is characterized by an intense fibrinous broncho-and-pleuropneumonia, vascular injury, edema, and pleural effusion in the lung and pleural cavity marked by significant leukocytic inflammation as observed histopathologically (176). To date, the most comprehensive, published, transcriptomic analysis comparing healthy versus *Mmm* infected animals was done using peripheral blood (8). Using whole blood collected from six cattle before contact challenge, and again at the onset of clinical disease, the study identified 680 differentially expressed annotated genes, with the overwhelming majority being downregulated (544, ~80%), characterizing an immunosuppressive environment. Pro-inflammatory markers such as TNFα, TLR2, IL-12B and IL-6 were not found to be upregulated in the blood of infected animals, despite a clear inflammatory environment being observed in infected lungs. Indeed, immunohistochemical analysis indicates proinflammatory cytokines TNFα, IL-1β and IL-17A are prevalent in lung lesions (176). Given that transcriptomic analysis of peripheral blood does not reflect the host response at the local site of infection, we aimed to assess the pulmonary host response *in situ*.

Our study provides a lesion-stage-resolved transcriptomic framework for understanding the progression of lung lesions during Contagious Bovine Pleuropneumonia (CBPP). Our data reveal how *Mycoplasma mycoides* subsp. *mycoides* infection drives a coordinated sequence of inflammatory injury, immune modulation, and structural remodeling in the bovine lung that culminates in characteristic lung pathology. By assessing differential gene expression across lesions of differential maturity, we obtain a temporal look at the host response to infection, and how the inflammatory landscape in the lung evolves from the initial moments of colonization and pathogen recognition down to tissue repair following pathologic sequelae. We show that each lesion stage is defined by a molecular program that aligns closely with its pathological phenotype and reflects the hosts shifting priorities as the disease progresses (**Figure 10**). However, transcriptional programs associated with pathogen recognition, interferon stimulated pathways, and innate immune responses persist throughout lesion progression, indirectly indicating chronic persistence of *Mmm* in infected tissue, despite an overwhelming inflammatory response. Indeed, we were able to culture Mmm as well as detect Mmm nucleic acid via PCR and sequencing within these lesions (**Table 1**), indicating that Mmm survives the inflammatory onslaught that develops in the infected lung. Indeed, previous reports have indicated the same (177). This further substantiates the idea that infection-induced host responses to mycoplasma infections are maladaptive, serving to contribute to immunopathology without contributing to bacterial clearance.

**Figure 10:**
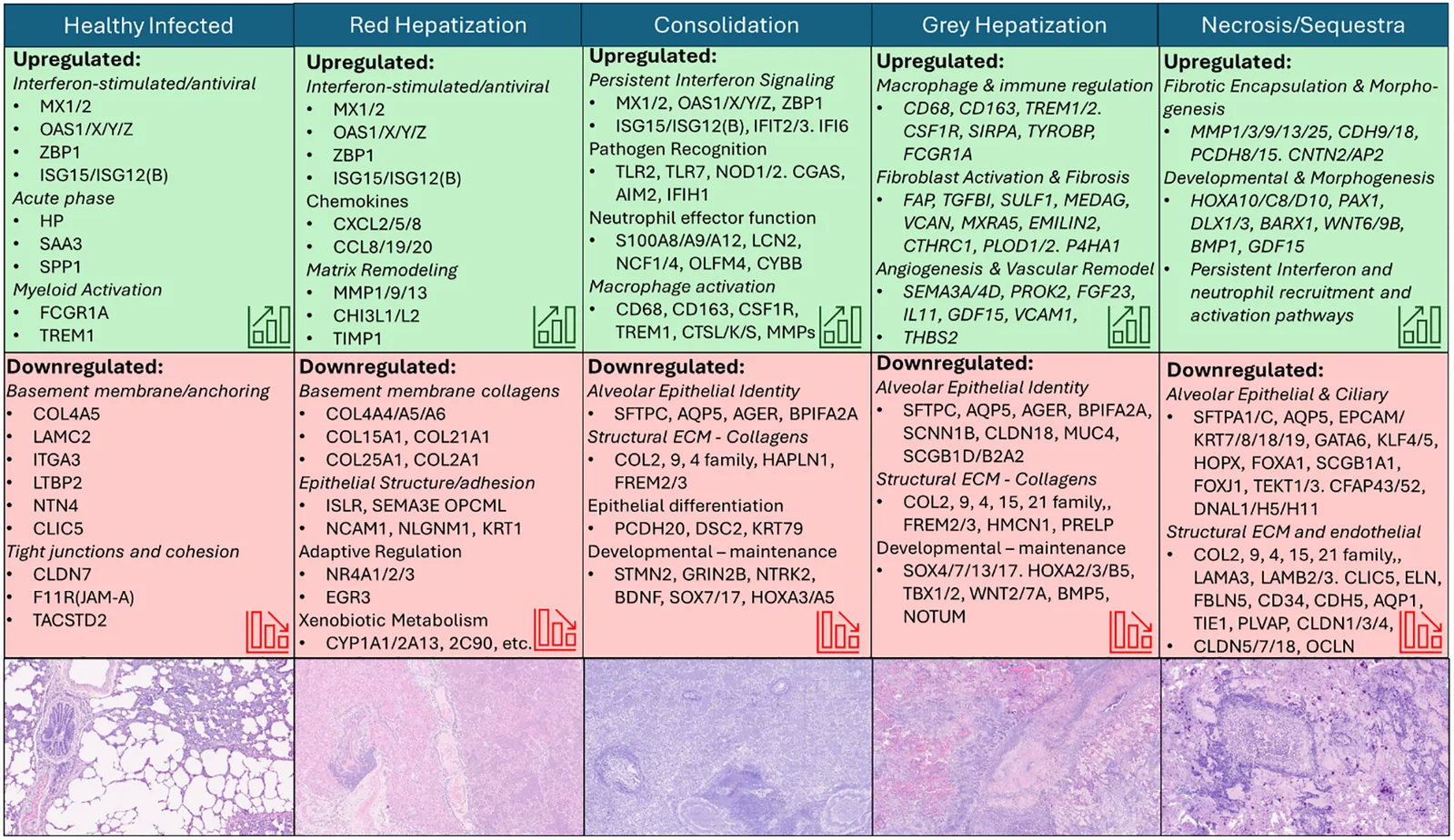
Summary of key findings per lesion stage and representative histopathological appearance of analyzed lung lesions.

Early lesions (Healthy Infected, Red Hepatization, and to an extent Consolidation) were dominated by a strong induction of innate immune pathways, including interferon stimulated gene expression, neutrophil recruitment, and inflammatory cytokines. Acute phase protein transcripts were significantly upregulated throughout infected lung tissue in our study, and previous studies have proteomically confirmed their expression in pleural effusion fluid collected from cattle with CBPP (178). Several pro-inflammatory and neutrophil associated transcripts such as IL1B, CXCL8, and NOS2 were found to be upregulated in our study, consistent with reports that studied *in vitro* exposures of bovine polymorphonuclear cells (PMNs) and lung explants to *Mmm* (9, 179). These signatures reflect an intense but ultimately ineffective attempt to control *Mmm*, consistent with the chronic nature of disease. Concurrent upregulation of early extracellular matrix remodeling pathways aligns with the gross pathological characteristics of CBPP. Grey hepatization represented a transitional state in which inflammatory programs remained active but were increasingly accompanied by macrophage-and-fibroblast-mediated profibrotic processes, extracellular matrix deposition and early tissue remodeling. However, continued pathogen persistence was hinted by the retained antiviral response signatures and neutrophil-associated transcripts. Unlike earlier stages, where adaptive immune responses were repressed, grey hepatization revealed the upregulation of transcriptional pathways that point toward mobilization of T and B cell responses, and initiation of adaptive immunity. The concurrent persistence of pathways involved in extracellular matrix destruction with reparative morphogenetic and angiogenic pathways indicate healing attempts with the goal of preserving tissue function. However continued pathogen persistence likely does not allow for functional tissue repair, thereby shifting the host response toward terminal tissue necrosis and sequestrum formation. The transcriptional landscape of Necrotic/Sequestra revealed a profound loss of epithelial identity of lung tissue, including suppression of surfactant genes, mucociliary machinery, epithelial tight-junctions, and epithelial lineage-defining transcription factors. These changes reflect the destruction and functional loss of airway and alveolar epithelium within the necrotic cores that form from cell death. Profibrotic processes were upregulated, driving remodeling processes associated with non-functional scar tissue formation rather than functional tissue repair. In an effort to contain infection and isolate necrotic tissue, this mature lesion reflected a transcriptional response focused on encapsulation and sequestra formation which is characteristic of chronic CBPP. Our transcriptomic findings align very closely with pathomorphological changes in infected lungs that are apparent in our own histopathological analysis (**Supplementary Figure S5**) and have been previously described by other groups (176). Consistent with the observed increase in leukocyte transcripts, the lung parenchyma and stroma frequently contained variable amounts of neutrophils, macrophages, and lymphocytes, further highlighting the massive inflammatory landscape in the lung. Necrotic epithelial cell debris was noted in the bronchioles and alveoli, consistent with disappearing epithelial cell transcriptional signatures as the lesions matured. As indicated by increases in fibroblast associated and pro-fibrotic transcriptional signatures, histopathological examination also revealed fibrinous bronchopneumonia, fibrinous pleuritis, and fibrotic dilated interlobular septa, especially in more mature lesions (grey hepatization, necrosis). Thrombosis and vasculitis of blood vessels in the interstitial septa was often noted, and occasionally granulation tissue was evident in the lung. This was reflected in our transcriptomic data by enrichment of transcripts associated with extracellular matrix organization and morphogenetic and angiogenic pathways. These features are all characteristic pathomorphological changes for CBPP, further validating our transcriptomic observations. Across all stages, the prominence of unannotated LOC- transcripts, many of which were strongly differentially expressed, highlights a substantial gap in bovine genome annotation and indicates that key regulatory elements involved in lung injury and chronic remodeling remain uncharacterized. Their consistent expression patterns indicate that *Mmm* infection activates transcriptional programs that extend beyond currently annotated pathways, highlighting the need for further research in the area, as our limited knowledge about the function of these gene products limits our interpretation of the data we present herein.

Despite these limitations, beyond defining the transcriptomic progression of CBPP disease, our work provides several important contributions to the field. First, it establishes the first comprehensive stage-resolved transcriptomic atlas of CBPP to date, linking classical pathological findings with their underlying molecular programs. This framework offers a reference point for future studies seeking to understand how *Mmm* may manipulate host immunity and tissue architecture over time. While the idea that immunopathology plays a role in disease due to mycoplasma infections is not new in the field, our work significantly substantiates the hypothesis. Uniquely, our study revealed an aspect of *in vivo* Mmm pathogenesis that has not been previously appreciated in the field owing to a scarcity of global transcriptomics studies at the local site of infection. That is, that *Mmm* infection induces an overwhelming anti-viral like transcriptional signature in the lung which is dominated by type 1 interferon and interferon stimulated gene transcripts such as IFNAR1, IFNAR2, STAT1, STAT2, IRF9, ISG15, ISG12(B), MX1, MX2, OAS1, OAS2, and several members of IFIT (interferon induced proteins with tetratricopeptide repeats) and IFI (interferon inducible) transcripts. While IFN-γ production by PBMCs and CD4+ T cells has been reported during stimulation or infection with Mmm, we have not been able to find references in the literature that reports upregulation of type-I interferon responses that mediate the antiviral environment we observe here (180, 181). Notably The significantly low p-values and high absolute fold changes of these transcripts indicate they are the predominant host response to *Mmm* infection. However, as we are able to detect viable *Mmm* across all lesion types, these responses do not appear to be protective against *Mmm*. Indeed, type I interferon induced responses may sometime suppress bactericidal functions that are critical for bacterial clearance (182). It is possible, therefore, that *Mmm* infection induces maladaptive antiviral host responses as a means of immune evasion to facilitate chronic infection. The hypothesis that Mycoplasmas induce dysregulated immune responses to facilitate infection is one that the community has long considered, and here we provide solid data to indicate this to be the case for *Mmm* infection in the natural bovine host. More mechanistic studies are needed to confirm this hypothesis, as the answer may be essential for informing the development of efficacious therapeutic or prophylactic methods to help control the disease. Furthermore, by providing a global look at the host response to *Mmm* infection, our data provide opportunity for biomarker discovery that can inform diagnostic development.

Altogether, these contributions position our work as a resource for the CBPP community and as a conceptual advance for understanding chronic respiratory mycoplasmoses. The molecular roadmap presented here provides a platform for hypothesis generation, comparative studies, and translational efforts aimed at mitigating the burden CBPP places on animal health and the agricultural industry.

## Materials and methods

### Study design and samples

Fourteen Small East African Zebu cattle (>12 months of age) were sourced from CBPP-free zones in western Kenya and enrolled in the study. Prior to inclusion, all animals were confirmed seronegative for CBPP using the complement fixation test (CFT). Animals were dewormed and vaccinated against foot-and-mouth-disease (FMD) according to routine husbandry practices and were housed under standard conditions with ad libitum access to hay, water and concentrate feed.

Mycoplasma mycoides subsp. mycoides (*Mmm*) strain Afade (183) was expanded in 100mL of PPLO broth to achieve approximately 1x10^8^ color-changing units (CCU)/mL and frozen. Twenty-four hours prior to infection, the frozen culture aliquots were used to initiate fresh 100mL cultures for animal challenge. Only freshly propagated cultures were used for infection as preliminary assessments demonstrated reduced viability in thawed cultures.

Animals were randomly allocated into two experimental groups and challenged via nebulization with sterile growth medium (n=4) to serve as uninfected controls while others (n=10) were challenged with *M*mm. Aerosol challenge was administered using a three-jet Collison nebulizer (CH Technologies, Westwood, NJ, USA) integrated into a face-mask exposure system developed at the Vaccine and Infectious Disease Organization (VIDO), University of Saskatchewan. The nebulizer was loaded with freshly cultured Mmm at 1x10^8^ CCU/mL and operated using medical grade oxygen at 20 psi. Aerosolized inoculum was delivered directly through a close-fitting face mask, while exhaled air was directed through a disinfectant-containing impinger to minimize environmental contamination. The system generated a respirable aerosol predominantly consisting of droplets measuring approximately 1 to 3 um in diameter, facilitating deposition within the respiratory tract.

Prior characterization of the nebulization system demonstrated stable aerosol production and maintenance of *Mmm* viability during aerosolization. Based on these studies and subsequent animal infection experiments (Gicheru et al., forthcoming manuscript), a single three-minute nebulization exposure was selected as the standard challenge protocol. This exposure duration consistently produced clinical disease, pathological lesions, and bacteriological recovery from animals while minimizing animal handling and restraint. Previous studies have shown that animals develop disease similar to that observed in the field when challenged via aerosols (184), hence we chose this option for our study.

Following infection, all animals were monitored daily for clinical signs of disease. Humane endpoint criteria included: endpoint criteria included: (i) rectal temperature ≥39.5 °C for six consecutive days, with a single intervening day below 39.5 °C considered part of the febrile period if preceded and followed by temperatures ≥39.5 °C; (ii) rectal temperature >40 °C for more than three consecutive days; (iii) lateral recumbency lasting more than one day; or (iv) failure to feed for three consecutive days. Animals reaching humane endpoint criteria were humanely euthanized by intravenous administration of sodium pentobarbitone (200 mg/mL) at a dose of 100 mg/kg body weight, with death confirmed by a veterinarian. Animals that did not reach humane endpoints were maintained until 42 days post-infection, after which they were euthanized and subjected to complete post-mortem examination.

Lung tissue samples (n = 81) were collected from necropsied cattle representing six pathological conditions: A_HlthU/Healthy Uninfected (Healthy tissue from uninfected animals), B_HlthI/Healthy Infected (Healthy looking tissue from infected animals), C_RedH/Red Hepatization), D_GreyH/Grey Hepatization), E_Cons/Consolidation and F_NecSeq/Necrotic tissue and sequestra). Pathological samples were identified by consensus of two independent, study-blind pathologists experienced with CBPP pathology. Sample metadata are provided in **Supplementary Table S1**. All animal procedures were performed under the approved institutional animal care and use (IACUC) protocol (ILRI-IACUC2024-23/1).

### *Mmm* isolation and molecular confirmation

Detection and molecular confirmation of (*Mmm*) were performed on tissue samples collected during necropsy. The recovery and detection of *Mmm* from challenged animals confirmed successful establishment of infection following aerosol exposure and supported the effectiveness of the nebulization challenge model. Detection of *Mmm* was conducted first through culturing from lung tissue in selective PPLO broth supplemented with penicillin, thallic acetate and amphotericin B, then on agar plates to confirm colony morphology. Cultured bacteria were confirmed by PCR to be *Mmm*. Genomic DNA was also extracted from lung tissue samples using a Proteinase K lysis buffer protocol. Initial screening was performed using PCR amplification of the 16S rRNA gene with the Mycoplasma-specific primer pair GC-341F (5′-CGCCCGCCGCGCGCGGCGGGCGGGGCGGGGGCACGGGGGGCCTACGGGAGGCAGCAG-3′) and R543 (5′-ACCTATGTATTACCGCG-3′), as described by McAuliffe et al., 2005 (185). This assay was used for genus-level detection of members of the class Mollicutes and served as a broad screening method for the presence of Mycoplasma DNA. Samples positive by the 16S rRNA PCR were subsequently subjected to species-specific confirmation using primers targeting the lppQ gene, a species-specific marker of M. mycoides subsp. mycoides previously described by Abdo et al., 2000 (186). Amplification of the expected lppQ product was used to confirm the presence of *Mmm* DNA within tissue samples. The sequential application of a genus-level 16S rRNA PCR followed by *Mmm*-specific lppQ PCR enabled reliable molecular identification of the challenge organism in infected animals.

### RNA extraction, sequencing, and preprocessing

Collected tissues were immediately placed into TRIzol at the time of collection and frozen at -80C until all tissues from all experimental animals were collected. In preparation for RNA extraction, samples were defrosted at room temperature, tissues were minced with scissors, then placed back into the original TRIZol aliquot and refrozen overnight. The next day, samples were thawed at room temperature and RNA was extracted using a modified phenol/chloroform extraction protocol from the recommended Invitrogen TRIzol Reagent User Guide. Briefly, the TRIzol supernatant was transferred to a new sterile tube and 200uL of both chloroform and tissue culture grade water was added. Samples were vigorously mixed then incubated at room temperature for 3 minutes. Samples were centrifuged at 12000xg for 15 minutes to separate the phases. The upper, colorless aqueous phase was collected and transferred to a new tube, and the chloroform step was repeated to wash the fraction. The samples were treated with DNAase I to remove contaminating DNA, then RNA was precipitated by adding 500uL of isopropanol, mixing vigorously, then incubating at room temperature for 10 minutes. The RNA was pelleted via centrifugation at 12000xg for 10 minutes. The pellet was resuspended in 1mL 75% ethanol, vortexed briefly, and centrifuged for 5 minutes at 7500xg. This step was repeated twice to sufficiently wash the RNA pellet, which was then air-dried for 7 minutes to evaporate the remaining ethanol. The pellets were resuspended in 50uL of tissue culture grade water and incubated in a heat block at 60C for 15 minutes. RNA concentrations and quality were measured using NanoDrop, then verified with fluorometric quantitation using the Qubit fluorometer. Dual rRNA depleted RNA libraries were prepared using the Illumina Stranded Total RNA Prep with Ligation, Ribo-Zero Plus Microbiome kit following manufacturer recommended instructions and samples were sequenced using the P4 NextSeq2000 P4 cartridges targeting 100M paired-end reads of 100-150bp in length per sample. Qubit quantification of RNA following rRNA depletion and of cDNA following library preparation indicated successful sample preparation.

### Data analyses

Raw FASTQ files were quality trimmed using Trimmomatic v0.39 with the following parameters: ILLUMINACLIP: IlluminaStrandedTotalRNA.fa:2:30:10:2:true, HEADCROP:1, LEADING:3. TRAILING:3. SLIDINGWINDOW:4:15, MINLEN:36. Trimmed reads were aligned to the to the *Bos taurus* (ARS-UCD1.2) reference genome using HISAT2 v2.2.1 (Ref). Genome indexes were generated using hisat-2 and paired-end reads were aligned using default parameters with strand specificity set according to library preparation. Gene level quantification was performed using featureCounts (Subread v2.0.3) with settings -p -s 2 -B -C to enforce paired-end counting, strandedness and chimeric read handling. Annotation was based on Ensembl GTF release 110. While Small East African Zebus are a *Bos indicus* species, we observed no significant differences in alignment rates between *Bos taurus* and *Bos indicus*, and given the better annotation of the *Bos taurus* genome, decided to move forward with the *Bos taurus* alignment. As an additional quality control step, samples with unique alignment of less than 50% were removed from analysis. Differential gene expression analysis was conducted using DESeq2 version 1.50.2 which models count data with a negative binomial generalized linear model and estimates gene-specific dispersion parameters using an empirical Bayes shrinkage procedure. This approach stabilized variance across conditions and is robust to unequal sample size and animal-animal variability because dispersion estimates for groups with fewer replicates borrow strength from the global mean-variance trend. After model fitting, log2 fold-changes are stabilized using apeglm shrinkage, which reduces exaggerated effect sizes for genes with high dispersion or low counts without altering statistical significance. This produces more reliable and interpretable fold-change estimates while preserving the underlying differential expression results. Differentially expressed genes (DEGs) were defined as those with absolute log2FC of greater than or equal to 2, and false-discovery-rate adjusted p values of less than 0.05.Gene Ontology Enrichment Analysis was performed using clusterProfiler version 4.18.4 using the human biological process (BP) GO universe after ortholog mapping (187–190). Cell-type deconvolution was performed using a z-score–based approach applied to variance-stabilized transcript (VST) expression values (see **Supplementary File** for methods and R-code). Volcano plots and heatmaps were generated in R using built in plotting functions using ggplot.

## Data Availability

The datasets presented in this study can be found in the NCBI Sequence Read Archive (SRA) under Bioproject number PRJNA1543624: In situ transcriptomic profiling of lung lesions from cattle experimentally infected with Mycoplasma mycoides subsp. mycoides.
