# Supplementary material for: Host transcriptional programs underlying lesion development in contagious bovine pleuropneumonia
Source: Front Immunol. 2026 Oct 30;17:1844390. doi: 10.3389/fimmu.2026.1844390 (PMC13759428; doi:10.3389/fimmu.2026.1844390)
Supplement: Supplementary file 1 [file Supplementaryfile1.docx]

**Supplementary Information for:**

***Host transcriptional programs underlying lesion development in contagious bovine pleuropneumonia***

**Authors:**

Arlind B. Mara^1,*,^, Angela Makumi^2,*^, R. Grace Ozyck^1,*^, Massimo Scacchia^3^, Hezron Wesonga^4^, Mark Ackermann^5^, Noah O. Okumu^2^, Winnie Chebore^2^, Morgan Hunte^1^, Jeremy M. Miller^1^, Edan R. Tulman^1^, Steven M. Szczepanek^1^, Elise Schieck^2,$^, Steven J. Geary^1,$^

**Author Affiliations:**

^1^ The Center of Excellence for Vaccine Research, Department of Pathobiology, University of Connecticut, Storrs, Connecticut, USA.

^2^ The International Livestock Research Institute, Nairobi, Kenya.

^3^ Istituto Zooprofilattico Sperimentale dell'Abruzzo e del Molise G. Caporale, Teramo, Italy.

^4^ Kenya Agricultural and Livestock Research Organization, Nairobi, Kenya.

^5^Department of Veterinary Pathology, College of Veterinary Medicine, Iowa State University, Ames, Iowa, USA.

^$^Corresponding Authors

SJG: [steven.geary@uconn.edu](mailto:steven.geary@uconn.edu)

ES: e.schieck@cgiar.org

*Equal Contribution

**Author Contributions:**

Conceptualization (*A.B.M., R.G.O., E.R.T., S.M.S., S.J.G.*)

Methodology (*A.B.M, R.G.O., E.R.T., S.M.S., S.J.G.*)

Software (*A.B.M.*)

Formal Analysis (*A.B.M.*)

Validation (*A.B.M., E.S., E.R.T., S.M.S., S.J.G.*)

Investigation (*A.B.M, A.M., R.G.O., M.S., H.W., M.A., N.O.O., W.C., M.L.H., J.M.M., E.S.*)

Resources (*S.J.G., E.S.*)

Data Curation (*A.B.M, A.M.*)

Writing – Original Draft (*A.B.M*)

Writing – Review & Editing (*A.B.M, A.M., R.G.O., M.S., H.W., M.A., N.O.O., W.C., M.L.H., J.M.M., E.R.T., S.M.S., E.S., S.J.G.*)

Vizualization (*A.B.M.*)

Supervision (*E.S., S.J.G.*)

Project Administration (*A.B.M., A.M., E.R.T., S.M.S., E.S., S.J.G.*)

Funding Acquisition (*S.J.G., S.M.S., E.R.T*.)

**Supplementary Methods: Cell-type Deconvolution**

Raw gene-level count matrices and metadata were imported into R (v4.5.2). Count matrix columns were aligned to metadata by exact matching of SampleID, and both objects were subset and reordered to ensure one-to-one correspondence. All downstream analyses were performed on this aligned dataset. Cell-type marker genes were curated from the Cattle Cell Atlas (CattleCA) for a signature list delineating: Ciliated Cells, Type I Pneumocytes, Type II Pneumocytes, Mast Cells, Plasma Cells, Proliferative Cells, Club Cells, Lymphatic and Vascular Endothelium, NK Cells, Fibroblasts, γδ T cells (WC1+), B Cells, Macrophages, CD4 T cells, CD8 T cells, and neutrophils. For each signature, only genes present in the dataset were retained. To stabilize variance across the dynamic range of expression values, raw counts were transformed using log_2_(count+1). For each gene, a Z-score was computed across all samples. Genes with zero variance were assigned a value of 1 to avoid undefined values. For each cell-type signature, enrichment scores were calculated as the mean Z-score of all genes belonging to that signature within each sample. To evaluate cell-type trends across disease progression, enrichment scores were aggregated by GroupID.

*R Code Used:*

**# 0. Setup and directories**

library(pheatmap)

count_file	<- "count_matrix_filtered.csv"

metadata_file <- "metadata_filtered.csv"

out_dir <- "Z_en_scRNA_Tcsplit"

if (!dir.exists(out_dir)) dir.create(out_dir, recursive = TRUE)

**# 1. Load count matrix & metadata**

metadata <- read.csv(metadata_file, header = TRUE, stringsAsFactors = FALSE)

counts_raw <- read.csv(count_file, header = TRUE, stringsAsFactors = FALSE, check.names = FALSE)

\# Assume first column = gene IDs

rownames(counts_raw) <- counts_raw[[1]]

counts_raw <- counts_raw[, -1, drop = FALSE]

\# Align samples

common_samples <- intersect(colnames(counts_raw), metadata$SampleID)

counts <- counts_raw[, common_samples, drop = FALSE]

metadata_sub <- metadata[match(common_samples, metadata$SampleID), ]

rownames(metadata_sub) <- metadata_sub$SampleID

saveRDS(counts, file = file.path(out_dir, "counts_aligned.rds"))

saveRDS(metadata_sub, file = file.path(out_dir, "metadata_aligned.rds"))

**# 2. Define scRNA signatures**

celltype_signatures_raw <- list(

Ciliated_Cells = c("CFAP299","PHGR1","PIGR","SEC14L3","TUBA1D","CETN4",

"LRRIQ1","RARRES1","CAPS","FAM183A","TPPP3","FOXJ1","CCDC78"),

TypeI_Pneumocyte = c("AGER","CYP4B1","GSTA2","GSTT2","CYP2B6","HSD17B13",

"LMO7","SPADH1","KRT7","CLDN18","ADIRF","CLIC5","ICAM1"),

Mast_Cells = c("PTI","TPSB2","LTC4S","VIPR2","FCER1A","GZMB","KIT"),

TypeII_Pneumocyte = c("SFTPC","SFTPA1","WFDC18","S100G","SFTPB","SLC34A2",

"SCD","SFTPD","NPC2","SFTA2","ABCA3","MUC1"),

Plasma_Cells = c("TXNDC5","JCHAIN","MZB1"),

Proliferative = c("TMSB10","TOP2A","CENPF","MKI67","PCLAF","UBE2C",

"SPC24","TPX2","STMN1","BIRC5","PTTG1"),

Club_Cells = c("SCGB1A1","SCGB2A2","SCGB1D","SCGB3A2","CLDN4","BPIFA1",

"KRT15","BPIFB1","TFF3","AOX2"),

Lymphatic_Endothelial = c("LYVE1","CCL21","MMRN1","PTN","RELN","S100A1",

"PKHD1L1","CCN3","PKDCC","TNFAIP8L3","APOLD1","PDPN"),

Vascular_Endothelium = c("CYYR1","CALCRL","CCDC85A","SMAD6","MYZAP","LDB2",

"HMCN1","PRKG1","RAMP3","PRICKLE2","PECAM1","KDR","FLT1"),

NK_Cells = c("NKG2A","DNAJC6","IDO1","KLRB1","TXK","NCR1","CX3CR1","FHIP1A","KLRK1"),

Fibroblasts = c("DCN","COL1A1","MGP","COL3A1","COL1A2","PI16","C1S","C1R",

"FBLN1","CFH","PDGFRA","FAP","TGFBI","SULF1","MEDAG"),

gdT_WC1pos = c("WC1.3","RHEX","BLK","PLCL1","CD163L1","SLC16A10"),

B_Cells = c("MS4A1","PAX5","TNFRSF13C","SOX5","BLNK","MYO1E","BANK1",

"BCL11A","FCRL1","EBF1","MA4A1/CD19","CD21","CD79B","CD79A"),

Macrophages = c("SGMS2","CD5L","MARCO","C1QB","C1QA","C1QC","LYZ","CD36",

"CD68","HMOX1","MS4A7","MRC1","MSR1","CD74","CD14","CD16","FCGR3A"),

CD4_T_Cells = c("CTLA4","CD28","LEF1","CD4","ICOS","CAMK4","SLC37A3",

"INPP4B","MAF","CD5","IL7R","CD3E"),

CD8_T_Cells = c("CTSW","CRTAM","CD8B","GZMM","CD96","CCL5","PRF1","THEMIS",

"NKG7","GNLY","CD8A","CD3G","CD3D","CSF1R"),

Neutrophils = c("HSD11B1","SDS","MEFV","GPR84","TGM3","RUBCNL","TCN1",

"CXCR1","CXCL8","CSF3R","S100A9","S100A8","S100A12","IL1RN")

)

\# Harmonize case

rownames(counts) <- toupper(rownames(counts))

celltype_signatures <- lapply(celltype_signatures_raw, toupper)

\# Intersect with available genes

celltype_signatures_filtered <- lapply(celltype_signatures, function(g)

intersect(g, rownames(counts))

)

signature_sizes <- sapply(celltype_signatures_filtered, length)

write.csv(data.frame(Signature = names(signature_sizes),

N_genes = signature_sizes),

file = file.path(out_dir, "signature_sizes.csv"),

row.names = FALSE)

saveRDS(celltype_signatures_filtered,

file = file.path(out_dir, "celltype_signatures_filtered.rds"))

**# 3. Compute per-gene Z-scores**

counts_mat <- as.matrix(counts)

mode(counts_mat) <- "numeric"

log_counts <- log2(counts_mat + 1)

gene_means <- rowMeans(log_counts)

gene_sds	<- apply(log_counts, 1, sd)

gene_sds[gene_sds == 0] <- 1

z_mat <- sweep(log_counts, 1, gene_means, "-")

z_mat <- sweep(z_mat, 1, gene_sds, "/")

saveRDS(z_mat, file = file.path(out_dir, "z_scores_gene_by_sample.rds"))

**# 4. Signature enrichment (mean Z)**

signature_names <- names(celltype_signatures_filtered)

sig_by_sample <- matrix(NA_real_,

nrow = length(signature_names),

ncol = ncol(z_mat),

dimnames = list(signature_names, colnames(z_mat)))

for (sig in signature_names) {

g <- celltype_signatures_filtered[[sig]]

if (length(g) > 0)

sig_by_sample[sig, ] <- colMeans(z_mat[g, , drop = FALSE])

}

sig_by_sample_df <- as.data.frame(sig_by_sample)

write.csv(sig_by_sample_df,

file = file.path(out_dir, "signature_enrichment_by_sample.csv"))

saveRDS(sig_by_sample_df,

file = file.path(out_dir, "signature_enrichment_by_sample.rds"))

**# 5. GroupID aggregation**

group_order <- c("A_HlthU","B_HlthI","C_RedH","E_Cons","D_GreyH","F_NecSeq")

group_vec <- metadata_sub$GroupID

names(group_vec) <- metadata_sub$SampleID

sig_by_group <- matrix(NA_real_,

nrow = nrow(sig_by_sample_df),

ncol = length(group_order),

dimnames = list(rownames(sig_by_sample_df), group_order))

for (g in group_order) {

samples <- names(group_vec)[group_vec == g]

if (length(samples) > 0)

sig_by_group[, g] <- rowMeans(sig_by_sample_df[, samples, drop = FALSE])

}

sig_by_group_df <- as.data.frame(sig_by_group)

write.csv(sig_by_group_df,

file = file.path(out_dir, "signature_enrichment_by_GroupID.csv"))

saveRDS(sig_by_group_df,

file = file.path(out_dir, "signature_enrichment_by_GroupID.rds"))

**# 6. Heatmaps**

\# Sample-level ordering

sample_order <- order(factor(metadata_sub$GroupID, levels = group_order),

metadata_sub$Time,

metadata_sub$AnimalID)

sig_by_sample_ord <- sig_by_sample_df[, sample_order, drop = FALSE]

metadata_ord	<- metadata_sub[sample_order, ]

annotation_col <- data.frame(

GroupID = factor(metadata_ord$GroupID, levels = group_order),

Time	= metadata_ord$Time,

Batch	= metadata_ord$Sequencing_Batch

)

rownames(annotation_col) <- metadata_ord$SampleID

\# Sample-level heatmap

pdf(file.path(out_dir, "heatmap_signature_by_sample.pdf"), width = 10, height = 6)

pheatmap(sig_by_sample_ord,

cluster_rows = TRUE,

cluster_cols = FALSE,

annotation_col = annotation_col,

show_colnames = FALSE,

main = "Cell-type Z-enrichment (Sample-level)")

dev.off()

\# Group-level heatmap

pdf(file.path(out_dir, "heatmap_signature_by_GroupID.pdf"), width = 6, height = 6)

pheatmap(sig_by_group_df[, group_order, drop = FALSE],

cluster_rows = TRUE,

cluster_cols = FALSE,

main = "Cell-type Z-enrichment (GroupID means)")

dev.off()

**Supplementary Figure S1.** All sample Raw Read Count distribution indicating % unique alignment to the *Bos taurus* genome.

**Supplementary Figure S2.** QC filtered sample Read Count distribution indicating % unique alignment to the *Bos taurus* genome separated out by pathological condition.

**Supplementary Figure S3.** Principal Component Analysis indicating sample distribution by infection status (top) and pathological condition (bottom).

**Supplementary Figure S4.** Sample-to-Sample Distance Heatmap

**Supplementary Figure S5.** Pathomorphological features of transcriptomically analyzed lesions. (**A**) A representative panel of a gross lung cross-section displaying areas of red hepatization, grey hepatization, necrosis, and thickened and fibrotic interlobular septa and pleura. Note the characteristic marbelization and fibrotic features that develop during CBPP. (**B**) H&E stain of lung section from infected animals that appeared healthy upon gross examination. Note the mild alveolar septal wall thickening which signals early reactions to Mmm infection. (**C**) Histological appearance of a lung section identified as Red Hepatization lesion. Note the characteristic hemorrhage, edema, and leukocytic exudate in the airways that define this lesion. (**D**) Histological appearance of a lesion identified as Consolidation. Note the exuberant leukocyte infiltration in the lung parenchyma, plugging of airways by leukocytes and necrotic epithelial cell debris, as well as hyperplasia of bronchus associated lymphoid tissue characteristic of Mycoplasma infection. (**E**)Histological appearance of a lesion identified as Grey Hepatization. Note the breakdown of red blood cells in the airways, and the dilated, fibrotic interlobular septum and loss of structural integrity of lung parenchyma. (**F**) Histological appearance of a lung section identified as a necrotic lesion enveloped in a fibrotic sequestrum. Note the necrotic cell debris, fibrin deposition, and organizing pneumonia/granulation tissue.

**Supplementary Figure S6.** Z-score Signature Enrichment defining relative cellular make-up across CBPP lesion stages at the sample level.

**Supplementary Table S1**. *Enriched GO Terms for Healthy Infected versus Healthy Uninfected Comparisons.*

| **ID** | **Description** | **GeneRatio** | | **p.adjust** |
| --- | --- | --- | --- | --- |
| GO:0051607 | defense response to virus | | 21/143 | 8.33981E-28 |
| GO:0009615 | response to virus | | 21/143 | 1.1719E-27 |
| GO:0044419 | biological process involved in interspecies interaction between organisms | | 31/143 | 3.25801E-19 |
| GO:0043207 | response to external biotic stimulus | | 30/143 | 7.36874E-19 |
| GO:0051707 | response to other organism | | 30/143 | 7.36874E-19 |
| GO:0009607 | response to biotic stimulus | | 30/143 | 7.36874E-19 |
| GO:0045069 | regulation of viral genome replication | | 11/143 | 1.38554E-16 |
| GO:0045071 | negative regulation of viral genome replication | | 11/143 | 1.38554E-16 |
| GO:0048525 | negative regulation of viral process | | 11/143 | 2.71627E-16 |
| GO:0019079 | viral genome replication | | 11/143 | 5.09513E-16 |
| GO:1903900 | regulation of viral life cycle | | 11/143 | 6.7023E-14 |
| GO:0019058 | viral life cycle | | 12/143 | 9.39566E-14 |
| GO:0050792 | regulation of viral process | | 11/143 | 1.9642E-13 |
| GO:0016032 | viral process | | 12/143 | 4.59937E-12 |
| GO:0140888 | interferon-mediated signaling pathway | | 7/143 | 2.2727E-09 |
| GO:0098542 | defense response to other organism | | 16/143 | 1.81324E-08 |
| GO:0060337 | type I interferon-mediated signaling pathway | | 6/143 | 5.88557E-08 |
| GO:0071357 | cellular response to type I interferon | | 6/143 | 5.88557E-08 |
| GO:0034340 | response to type I interferon | | 6/143 | 9.22522E-08 |
| GO:0140546 | defense response to symbiont | | 15/143 | 1.08848E-07 |
| GO:0034341 | response to type II interferon | | 6/143 | 1.32572E-07 |
| GO:0045087 | innate immune response | | 14/143 | 2.36069E-07 |
| GO:0035456 | response to interferon-beta | | 5/143 | 8.04066E-07 |
| GO:0046597 | host-mediated suppression of symbiont invasion | | 5/143 | 8.04066E-07 |
| GO:0051851 | host-mediated perturbation of symbiont process | | 5/143 | 1.31381E-06 |
| GO:0042130 | negative regulation of T cell proliferation | | 5/143 | 3.14785E-06 |
| GO:0035821 | modulation of process of another organism | | 5/143 | 4.51444E-06 |
| GO:0032945 | negative regulation of mononuclear cell proliferation | | 5/143 | 7.07414E-06 |
| GO:0050672 | negative regulation of lymphocyte proliferation | | 5/143 | 7.07414E-06 |
| GO:0070664 | negative regulation of leukocyte proliferation | | 5/143 | 7.07414E-06 |
| GO:0042098 | T cell proliferation | | 6/143 | 7.07414E-06 |
| GO:0042129 | regulation of T cell proliferation | | 6/143 | 7.07414E-06 |
| GO:0008285 | negative regulation of cell population proliferation | | 7/143 | 7.07414E-06 |
| GO:0034097 | response to cytokine | | 11/143 | 7.07414E-06 |
| GO:1901652 | response to peptide | | 11/143 | 7.07414E-06 |
| GO:0019221 | cytokine-mediated signaling pathway | | 10/143 | 8.02724E-06 |
| GO:0071345 | cellular response to cytokine stimulus | | 10/143 | 1.72033E-05 |
| GO:0002682 | regulation of immune system process | | 15/143 | 2.12386E-05 |
| GO:0032944 | regulation of mononuclear cell proliferation | | 6/143 | 4.24354E-05 |
| GO:0050670 | regulation of lymphocyte proliferation | | 6/143 | 4.24354E-05 |
| GO:0070663 | regulation of leukocyte proliferation | | 6/143 | 4.24354E-05 |
| GO:0050868 | negative regulation of T cell activation | | 5/143 | 4.66871E-05 |
| GO:1903038 | negative regulation of leukocyte cell-cell adhesion | | 5/143 | 4.66871E-05 |
| GO:0032943 | mononuclear cell proliferation | | 6/143 | 6.33237E-05 |
| GO:0046651 | lymphocyte proliferation | | 6/143 | 6.33237E-05 |
| GO:0070661 | leukocyte proliferation | | 6/143 | 6.33237E-05 |
| GO:0042110 | T cell activation | | 7/143 | 6.33237E-05 |
| GO:0022408 | negative regulation of cell-cell adhesion | | 5/143 | 7.57238E-05 |
| GO:0051250 | negative regulation of lymphocyte activation | | 5/143 | 0.000106032 |
| GO:0050863 | regulation of T cell activation | | 6/143 | 0.000108817 |
| GO:0002695 | negative regulation of leukocyte activation | | 5/143 | 0.000189597 |
| GO:0050866 | negative regulation of cell activation | | 5/143 | 0.000189597 |
| GO:0007162 | negative regulation of cell adhesion | | 5/143 | 0.000285229 |
| GO:0032309 | icosanoid secretion | | 4/143 | 0.000314865 |
| GO:0050482 | arachidonate secretion | | 4/143 | 0.000314865 |
| GO:1903963 | arachidonate transport | | 4/143 | 0.000314865 |
| GO:0002683 | negative regulation of immune system process | | 6/143 | 0.000352274 |
| GO:0001775 | cell activation | | 9/143 | 0.00040335 |
| GO:0002694 | regulation of leukocyte activation | | 7/143 | 0.000406834 |
| GO:0071715 | icosanoid transport | | 4/143 | 0.000447548 |
| GO:0050865 | regulation of cell activation | | 7/143 | 0.000508487 |
| GO:1903037 | regulation of leukocyte cell-cell adhesion | | 5/143 | 0.000645498 |
| GO:0045321 | leukocyte activation | | 8/143 | 0.000778729 |
| GO:0051249 | regulation of lymphocyte activation | | 6/143 | 0.001175348 |
| GO:0007159 | leukocyte cell-cell adhesion | | 5/143 | 0.001494776 |
| GO:0046649 | lymphocyte activation | | 7/143 | 0.001608389 |
| GO:0022407 | regulation of cell-cell adhesion | | 5/143 | 0.001879425 |
| GO:0015909 | long-chain fatty acid transport | | 4/143 | 0.002199281 |
| GO:0052547 | regulation of peptidase activity | | 5/143 | 0.003653752 |
| GO:0032496 | response to lipopolysaccharide | | 4/143 | 0.00420803 |
| GO:0042127 | regulation of cell population proliferation | | 9/143 | 0.005176789 |
| GO:0051241 | negative regulation of multicellular organismal process | | 6/143 | 0.005215816 |
| GO:0002237 | response to molecule of bacterial origin | | 4/143 | 0.005422903 |
| GO:0008283 | cell population proliferation | | 9/143 | 0.007721213 |
| GO:0030162 | regulation of proteolysis | | 6/143 | 0.011590389 |
| GO:0015908 | fatty acid transport | | 4/143 | 0.012000958 |
| GO:0006869 | lipid transport | | 7/143 | 0.01415157 |
| GO:0030155 | regulation of cell adhesion | | 5/143 | 0.015523121 |
| GO:0010876 | lipid localization | | 7/143 | 0.018680887 |
| GO:0010466 | negative regulation of peptidase activity | | 4/143 | 0.019501774 |
| GO:0050776 | regulation of immune response | | 8/143 | 0.019501774 |
| GO:0002252 | immune effector process | | 6/143 | 0.020685542 |
| GO:0009617 | response to bacterium | | 6/143 | 0.02187603 |
| GO:0006915 | apoptotic process | | 10/143 | 0.022544334 |
| GO:0051239 | regulation of multicellular organismal process | | 11/143 | 0.023085756 |
| GO:0046942 | carboxylic acid transport | | 6/143 | 0.023334218 |
| GO:0015849 | organic acid transport | | 6/143 | 0.023835247 |
| GO:0008219 | cell death | | 10/143 | 0.024705376 |
| GO:0012501 | programmed cell death | | 10/143 | 0.024705376 |
| GO:0071222 | cellular response to lipopolysaccharide | | 3/143 | 0.026174042 |
| GO:0045861 | negative regulation of proteolysis | | 4/143 | 0.026174042 |
| GO:0046634 | regulation of alpha-beta T cell activation | | 2/143 | 0.030562963 |
| GO:0006879 | intracellular iron ion homeostasis | | 3/143 | 0.030562963 |
| GO:0071219 | cellular response to molecule of bacterial origin | | 3/143 | 0.032793219 |
| GO:0050778 | positive regulation of immune response | | 7/143 | 0.032993569 |
| GO:0001959 | regulation of cytokine-mediated signaling pathway | | 2/143 | 0.034538069 |
| GO:0003254 | regulation of membrane depolarization | | 2/143 | 0.034538069 |
| GO:0060759 | regulation of response to cytokine stimulus | | 2/143 | 0.034538069 |
| GO:0045089 | positive regulation of innate immune response | | 4/143 | 0.034538069 |
| GO:0006644 | phospholipid metabolic process | | 7/143 | 0.034538069 |

**Supplementary Table S2.** *Relevant Differentially Expressed Genes for Healthy Infected versus Healthy Uninfected Comparisons.*

| **Gene** | **Change** | **log2 F.C.** | **p.adjusted** |
| --- | --- | --- | --- |
| SAA3 | Increased | 6.005 | 1.72312E-13 |
| CYM | Decreased | -10.018 | 4.99042E-09 |
| M-SAA3.2 | Increased | 5.43 | 1.06564E-08 |
| NTN4 | Decreased | -1.912 | 2.33537E-08 |
| PLA2G2D4 | Increased | 3.592 | 1.4319E-06 |
| HP | Increased | 3.553 | 4.24554E-05 |
| ZBP1 | Increased | 3.48 | 4.24554E-05 |
| OAS1Z | Increased | 3.107 | 9.42156E-05 |
| ISG15 | Increased | 3.655 | 0.000168436 |
| MX1 | Increased | 3.035 | 0.000206667 |
| OAS1Y | Increased | 2.853 | 0.000206667 |
| IFI6 | Increased | 2.787 | 0.000255557 |
| NR4A2 | Decreased | -1.628 | 0.000255557 |
| PLA2G5 | Increased | 4.713 | 0.000379031 |
| CILP2 | Decreased | -4.15 | 0.000865537 |
| OAS1X | Increased | 2.415 | 0.001484307 |
| APOBEC3Z1 | Increased | 3.823 | 0.001718113 |
| CLEC4F | Increased | 4.461 | 0.002005364 |
| LGALS9 | Increased | 1.757 | 0.002418581 |
| IFIT2 | Increased | 3.268 | 0.003240784 |
| TIMP1 | Increased | 3.004 | 0.003444879 |
| SOCS2 | Decreased | -1.118 | 0.003444879 |
| MX2 | Increased | 2.729 | 0.003525088 |
| RSAD2 | Increased | 2.538 | 0.020735251 |

**Supplementary Table S3**. *Enriched GO Terms for Red Hepatization versus Healthy Uninfected Comparisons.*

| **ID** | **Description** | **GeneRatio** | | **p.adjust** |  |
| --- | --- | --- | --- | --- | --- |
| GO:0009615 | response to virus | | 41/3787 | 1.24219E-13 |  |
| GO:0051607 | defense response to virus | | 39/3787 | 7.06803E-13 |  |
| GO:0002682 | regulation of immune system process | | 147/3787 | 6.5432E-10 |  |
| GO:0044419 | biological process involved in interspecies interaction between organisms | | 147/3787 | 1.32117E-09 |  |
| GO:0031347 | regulation of defense response | | 68/3787 | 3.38543E-09 |  |
| GO:0034097 | response to cytokine | | 79/3787 | 3.38543E-09 |  |
| GO:1901652 | response to peptide | | 79/3787 | 3.38543E-09 |  |
| GO:0080134 | regulation of response to stress | | 91/3787 | 4.96049E-09 |  |
| GO:0006954 | inflammatory response | | 70/3787 | 1.06552E-08 |  |
| GO:0032101 | regulation of response to external stimulus | | 85/3787 | 1.06552E-08 |  |
| GO:0045321 | leukocyte activation | | 71/3787 | 2.07105E-08 |  |
| GO:0001775 | cell activation | | 80/3787 | 2.07105E-08 |  |
| GO:0002684 | positive regulation of immune system process | | 117/3787 | 2.07105E-08 |  |
| GO:0009607 | response to biotic stimulus | | 138/3787 | 2.07105E-08 |  |
| GO:0043207 | response to external biotic stimulus | | 137/3787 | 2.07105E-08 |  |
| GO:0051707 | response to other organism | | 137/3787 | 2.07105E-08 |  |
| GO:0046649 | lymphocyte activation | | 61/3787 | 8.30733E-08 |  |
| GO:1903900 | regulation of viral life cycle | | 23/3787 | 1.46369E-07 |  |
| GO:0071345 | cellular response to cytokine stimulus | | 68/3787 | 1.46369E-07 |  |
| GO:0098542 | defense response to other organism | | 97/3787 | 1.98905E-07 |  |
| GO:0007155 | cell adhesion | | 173/3787 | 2.19796E-07 |  |
| GO:0045087 | innate immune response | | 88/3787 | 4.51215E-07 |  |
| GO:0140546 | defense response to symbiont | | 94/3787 | 9.44504E-07 |  |
| GO:0051249 | regulation of lymphocyte activation | | 43/3787 | 1.31367E-06 |  |
| GO:0002831 | regulation of response to biotic stimulus | | 45/3787 | 1.61312E-06 |  |
| GO:0034340 | response to type I interferon | | 14/3787 | 1.69797E-06 |  |
| GO:0050792 | regulation of viral process | | 23/3787 | 2.03215E-06 |  |
| GO:0050865 | regulation of cell activation | | 50/3787 | 2.03215E-06 |  |
| GO:0019221 | cytokine-mediated signaling pathway | | 61/3787 | 2.03215E-06 |  |
| GO:0098609 | cell-cell adhesion | | 118/3787 | 2.03215E-06 |  |
| GO:0044093 | positive regulation of molecular function | | 111/3787 | 2.46925E-06 |  |
| GO:0045069 | regulation of viral genome replication | | 16/3787 | 2.88496E-06 |  |
| GO:0045071 | negative regulation of viral genome replication | | 16/3787 | 2.88496E-06 |  |
| GO:0032944 | regulation of mononuclear cell proliferation | | 27/3787 | 3.02256E-06 |  |
| GO:0050670 | regulation of lymphocyte proliferation | | 27/3787 | 3.02256E-06 |  |
| GO:0070663 | regulation of leukocyte proliferation | | 27/3787 | 3.02256E-06 |  |
| GO:0045088 | regulation of innate immune response | | 42/3787 | 3.05422E-06 |  |
| GO:0002694 | regulation of leukocyte activation | | 48/3787 | 3.18323E-06 |  |
| GO:0060337 | type I interferon-mediated signaling pathway | | 13/3787 | 4.39475E-06 |  |
| GO:0071357 | cellular response to type I interferon | | 13/3787 | 4.39475E-06 |  |
| GO:0140888 | interferon-mediated signaling pathway | | 14/3787 | 6.72967E-06 |  |
| GO:0019058 | viral life cycle | | 26/3787 | 6.89663E-06 |  |
| GO:0032943 | mononuclear cell proliferation | | 28/3787 | 6.89663E-06 |  |
| GO:0046651 | lymphocyte proliferation | | 28/3787 | 6.89663E-06 |  |
| GO:0070661 | leukocyte proliferation | | 28/3787 | 6.89663E-06 |  |
| GO:0048525 | negative regulation of viral process | | 16/3787 | 8.07015E-06 |  |
| GO:0002252 | immune effector process | | 63/3787 | 8.21965E-06 |  |
| GO:0002833 | positive regulation of response to biotic stimulus | | 39/3787 | 1.03577E-05 |  |
| GO:0050776 | regulation of immune response | | 95/3787 | 1.35586E-05 |  |
| GO:0032103 | positive regulation of response to external stimulus | | 49/3787 | 1.63603E-05 |  |
| GO:1902531 | regulation of intracellular signal transduction | | 147/3787 | 1.70094E-05 |  |
| GO:0007159 | leukocyte cell-cell adhesion | | 30/3787 | 1.81499E-05 |  |
| GO:0045089 | positive regulation of innate immune response | | 37/3787 | 1.86958E-05 |  |
| GO:0051239 | regulation of multicellular organismal process | | 152/3787 | 1.97153E-05 |  |
| GO:0019079 | viral genome replication | | 16/3787 | 2.21139E-05 |  |
| GO:0031349 | positive regulation of defense response | | 40/3787 | 2.24899E-05 |  |
| GO:0050790 | regulation of catalytic activity | | 149/3787 | 3.21827E-05 |  |
| GO:0030036 | actin cytoskeleton organization | | 124/3787 | 3.65667E-05 |  |
| GO:0042110 | T cell activation | | 36/3787 | 3.67377E-05 |  |
| GO:0050778 | positive regulation of immune response | | 84/3787 | 4.44938E-05 |  |
| GO:0030029 | actin filament-based process | | 127/3787 | 5.24665E-05 |  |
| GO:0002218 | activation of innate immune response | | 32/3787 | 5.7127E-05 |  |
| GO:0022407 | regulation of cell-cell adhesion | | 30/3787 | 6.98827E-05 |  |
| GO:0002263 | cell activation involved in immune response | | 23/3787 | 8.28719E-05 |  |
| GO:0051241 | negative regulation of multicellular organismal process | | 48/3787 | 9.22042E-05 |  |
| GO:0051250 | negative regulation of lymphocyte activation | | 19/3787 | 0.000140202 |  |
| GO:0002221 | pattern recognition receptor signaling pathway | | 23/3787 | 0.000144809 |  |
| GO:0002366 | leukocyte activation involved in immune response | | 22/3787 | 0.000198213 |  |
| GO:0043408 | regulation of MAPK cascade | | 54/3787 | 0.000227485 |  |
| GO:1903706 | regulation of hemopoiesis | | 19/3787 | 0.000266072 |  |
| GO:0048585 | negative regulation of response to stimulus | | 114/3787 | 0.000283372 |  |
| GO:0002695 | negative regulation of leukocyte activation | | 20/3787 | 0.000349668 |  |
| GO:0050866 | negative regulation of cell activation | | 20/3787 | 0.000349668 |  |
| GO:0000165 | MAPK cascade | | 59/3787 | 0.000349668 |  |
| GO:1903037 | regulation of leukocyte cell-cell adhesion | | 24/3787 | 0.000463304 |  |
| GO:0002253 | activation of immune response | | 65/3787 | 0.000463304 |  |
| GO:0002683 | negative regulation of immune system process | | 31/3787 | 0.000541837 |  |
| GO:0030097 | hemopoiesis | | 40/3787 | 0.000569976 |  |
| GO:0043085 | positive regulation of catalytic activity | | 80/3787 | 0.000728807 |  |
| GO:0030155 | regulation of cell adhesion | | 40/3787 | 0.000755416 |  |
| GO:0050863 | regulation of T cell activation | | 26/3787 | 0.000861654 |  |
| GO:0002758 | innate immune response-activating signaling pathway | | 28/3787 | 0.000971013 |  |
| GO:0042098 | T cell proliferation | | 18/3787 | 0.001076651 |  |
| GO:0042129 | regulation of T cell proliferation | | 18/3787 | 0.001076651 |  |
| GO:0016477 | cell migration | | 117/3787 | 0.001076651 |  |
| GO:0050727 | regulation of inflammatory response | | 25/3787 | 0.001138037 |  |
| GO:0051251 | positive regulation of lymphocyte activation | | 25/3787 | 0.001138037 |  |
| GO:0044403 | biological process involved in symbiotic interaction | | 12/3787 | 0.001424533 |  |
| GO:0002275 | myeloid cell activation involved in immune response | | 11/3787 | 0.001699522 |  |
| GO:0016032 | viral process | | 27/3787 | 0.001849519 |  |
| GO:0035456 | response to interferon-beta | | 9/3787 | 0.001882249 |  |
| GO:0002696 | positive regulation of leukocyte activation | | 29/3787 | 0.001882249 |  |
| GO:0050867 | positive regulation of cell activation | | 29/3787 | 0.001882249 |  |
| GO:0050900 | leukocyte migration | | 31/3787 | 0.001882249 |  |
| GO:0002250 | adaptive immune response | | 32/3787 | 0.001882249 |  |
| GO:0009117 | nucleotide metabolic process | | 70/3787 | 0.001882249 |  |
| GO:0050864 | regulation of B cell activation | | 17/3787 | 0.00236608 |  |
| GO:0050868 | negative regulation of T cell activation | | 15/3787 | 0.002470792 |  |
| GO:1903038 | negative regulation of leukocyte cell-cell adhesion | | 15/3787 | 0.002470792 |  |
| GO:0051336 | regulation of hydrolase activity | | 77/3787 | 0.002475696 |  |

**Supplementary Table S4.** *Relevant Differentially Expressed Genes for Red Hepatization versus Healthy Uninfected Comparisons.*

| **Gene** | **Change** | **log2 F.C.** | | **p.adjusted** |
| --- | --- | --- | --- | --- |
| SAA3 | Increased | 7.936 | 3.24E-31 | |
| M-SAA3.2 | Increased | 7.944 | 3.86E-25 | |
| ISG12(B) | Increased | 6.884 | 1.24E-16 | |
| OAS1Z | Increased | 4.662 | 4.36E-16 | |
| NR4A2 | Decreased | -2.555 | 4.50E-16 | |
| SOD2 | Increased | 4.356 | 1.77E-15 | |
| ZBP1 | Increased | 4.904 | 2.55E-15 | |
| SPP1 | Increased | 7.286 | 6.10E-15 | |
| ISG15 | Increased | 5.361 | 7.11E-15 | |
| MX2 | Increased | 4.602 | 1.17E-13 | |
| IFIT2 | Increased | 5.43 | 1.17E-13 | |
| APOBEC3Z1 | Increased | 6.055 | 3.25E-13 | |
| MX1 | Increased | 4.213 | 3.70E-13 | |
| CD14 | Increased | 3.989 | 3.97E-13 | |
| TREM1 | Increased | 5.53 | 4.33E-13 | |
| CHI3L1 | Increased | 3.714 | 1.95E-12 | |
| OAS1Y | Increased | 3.833 | 2.64E-12 | |
| ISG20 | Increased | 4.232 | 4.50E-12 | |
| OAS1X | Increased | 3.462 | 2.38E-11 | |
| CLEC4F | Increased | 6.541 | 2.57E-11 | |
| COL4A5 | Decreased | -3.207 | 2.88E-11 | |
| MMP9 | Increased | 7.619 | 4.00E-10 | |
| RSAD2 | Increased | 4.167 | 7.39E-10 | |
| CYM | Decreased | -8.098 | 1.19E-09 | |
| PTX3 | Increased | 3.748 | 1.57E-09 | |
| CCL8 | Increased | 4.929 | 1.57E-09 | |
| COL25A1 | Decreased | -3.404 | 3.30E-09 | |
| MMP25 | Increased | 3.57 | 1.67E-08 | |
| NR4A1 | Decreased | -3.461 | 1.82E-08 | |
| COL4A6 | Decreased | -2.727 | 5.27E-08 | |
| CHI3L2 | Increased | 4.86 | 5.41E-08 | |
| NOS2 | Increased | 3.595 | 1.29E-07 | |
| SIGLEC1 | Increased | 4.377 | 2.68E-07 | |
| SEMA3E | Decreased | -2.903 | 3.89E-07 | |
| NR4A3 | Decreased | -3.029 | 1.20E-06 | |
| TLR7 | Increased | 2.618 | 1.33E-06 | |
| IL27 | Increased | 3.249 | 1.44E-06 | |
| EGR3 | Decreased | -2.879 | 2.47E-06 | |
| COL4A4 | Decreased | -2.233 | 4.81E-06 | |
| TLR2 | Increased | 2.023 | 8.60E-06 | |
| OPCML | Decreased | -3.603 | 1.08E-05 | |
| MMP13 | Increased | 3.849 | 2.54E-05 | |
| ISLR | Decreased | -2.902 | 3.07E-05 | |
| CCL19 | Increased | 3.335 | 5.83E-05 | |
| MMP1 | Increased | 5.878 | 5.97E-05 | |
| IL19 | Increased | 6.054 | 6.03E-05 | |
| IL17A | Increased | 4.98 | 7.98E-05 | |
| CXCL2 | Increased | 3.033 | 1.73E-04 | |
| CYP1A1 | Decreased | -4.02 | 1.74E-04 | |
| CYP2C90 | Decreased | -3.017 | 1.90E-04 | |
| COL21A1 | Decreased | -2.077 | 2.40E-04 | |
| COL15A1 | Decreased | -2.029 | 2.43E-04 | |
| CCL20 | Increased | 3.715 | 3.55E-04 | |
| CXCL5 | Increased | 2.19 | 3.80E-04 | |
| CYP2A13 | Decreased | -3.144 | 3.98E-04 | |
| SAA2 | Increased | 5.011 | 5.90E-04 | |
| KRT1 | Decreased | -3.668 | 7.16E-04 | |
| CXCL8 | Increased | 2.733 | 1.10E-03 | |
| IL21 | Increased | 2.749 | 2.74E-03 | |
| CYP3A24 | Decreased | -2.062 | 7.99E-03 | |
| COL2A1 | Decreased | -3.666 | 1.45E-02 | |
| NCAM1 | Decreased | -2.177 | 2.50E-02 | |
| NLGN1 | Decreased | -2.146 | 4.34E-02 | |
| PLA2G2D4 | Increased | 4.559 | 2.55E-15 | |
| PLA2G5 | Increased | 7.421 | 1.09E-14 | |
| NTN4 | Decreased | -2.345 | 3.43E-17 | |
| CILP2 | Decreased | -5.01 | 1.38E-08 | |
| TIMP1 | Increased | 4.82 | 1.32E-12 | |
| MMP15 | Decreased | -2.02 | 7.41E-06 | |
| MMP16 | Decreased | -3.08 | 1.91E-07 | |

**Supplementary Table S5**. *Enriched GO Terms for Consolidation versus Healthy Uninfected Comparisons.*

| **ID** | **Description** | **GeneRatio** | | **p.adjust** |
| --- | --- | --- | --- | --- |
| GO:0009615 | response to virus | | 37/3240 | 4.06618E-12 |
| GO:0051607 | defense response to virus | | 36/3240 | 4.06618E-12 |
| GO:0034097 | response to cytokine | | 74/3240 | 4.38365E-10 |
| GO:1901652 | response to peptide | | 74/3240 | 4.38365E-10 |
| GO:0044419 | biological process involved in interspecies interaction between organisms | | 130/3240 | 4.53334E-09 |
| GO:0071345 | cellular response to cytokine stimulus | | 65/3240 | 9.32186E-09 |
| GO:0006954 | inflammatory response | | 64/3240 | 9.32186E-09 |
| GO:0009607 | response to biotic stimulus | | 125/3240 | 9.32186E-09 |
| GO:0002682 | regulation of immune system process | | 127/3240 | 1.14222E-08 |
| GO:0043207 | response to external biotic stimulus | | 123/3240 | 2.08336E-08 |
| GO:0051707 | response to other organism | | 123/3240 | 2.08336E-08 |
| GO:0046649 | lymphocyte activation | | 57/3240 | 2.08336E-08 |
| GO:0045321 | leukocyte activation | | 64/3240 | 4.60256E-08 |
| GO:1903900 | regulation of viral life cycle | | 22/3240 | 8.33545E-08 |
| GO:0001775 | cell activation | | 71/3240 | 8.88109E-08 |
| GO:0019221 | cytokine-mediated signaling pathway | | 58/3240 | 1.85703E-07 |
| GO:0002684 | positive regulation of immune system process | | 102/3240 | 2.24113E-07 |
| GO:0045069 | regulation of viral genome replication | | 16/3240 | 4.57965E-07 |
| GO:0045071 | negative regulation of viral genome replication | | 16/3240 | 4.57965E-07 |
| GO:0098542 | defense response to other organism | | 86/3240 | 5.54524E-07 |
| GO:0045087 | innate immune response | | 79/3240 | 5.60973E-07 |
| GO:0051249 | regulation of lymphocyte activation | | 40/3240 | 6.15234E-07 |
| GO:0050792 | regulation of viral process | | 22/3240 | 8.88937E-07 |
| GO:0031347 | regulation of defense response | | 56/3240 | 1.10142E-06 |
| GO:0042110 | T cell activation | | 36/3240 | 1.30791E-06 |
| GO:0050865 | regulation of cell activation | | 46/3240 | 1.30791E-06 |
| GO:0048525 | negative regulation of viral process | | 16/3240 | 1.30791E-06 |
| GO:0032101 | regulation of response to external stimulus | | 71/3240 | 1.3111E-06 |
| GO:0140546 | defense response to symbiont | | 83/3240 | 2.32734E-06 |
| GO:0002694 | regulation of leukocyte activation | | 44/3240 | 2.82908E-06 |
| GO:0007159 | leukocyte cell-cell adhesion | | 29/3240 | 3.60493E-06 |
| GO:0019079 | viral genome replication | | 16/3240 | 3.7628E-06 |
| GO:0002221 | pattern recognition receptor signaling pathway | | 23/3240 | 1.58929E-05 |
| GO:0080134 | regulation of response to stress | | 72/3240 | 1.61404E-05 |
| GO:1902531 | regulation of intracellular signal transduction | | 130/3240 | 2.75012E-05 |
| GO:0032943 | mononuclear cell proliferation | | 25/3240 | 3.15823E-05 |
| GO:0046651 | lymphocyte proliferation | | 25/3240 | 3.15823E-05 |
| GO:0070661 | leukocyte proliferation | | 25/3240 | 3.15823E-05 |
| GO:0032103 | positive regulation of response to external stimulus | | 44/3240 | 3.27825E-05 |
| GO:0051604 | protein maturation | | 89/3240 | 3.5489E-05 |
| GO:0019058 | viral life cycle | | 23/3240 | 4.14812E-05 |
| GO:0002831 | regulation of response to biotic stimulus | | 38/3240 | 4.14812E-05 |
| GO:0050900 | leukocyte migration | | 32/3240 | 4.40037E-05 |
| GO:0098609 | cell-cell adhesion | | 100/3240 | 4.5953E-05 |
| GO:0030198 | extracellular matrix organization | | 49/3240 | 4.76214E-05 |
| GO:0045229 | external encapsulating structure organization | | 49/3240 | 4.76214E-05 |
| GO:0034340 | response to type I interferon | | 12/3240 | 5.43141E-05 |
| GO:0032944 | regulation of mononuclear cell proliferation | | 23/3240 | 6.02584E-05 |
| GO:0050670 | regulation of lymphocyte proliferation | | 23/3240 | 6.02584E-05 |
| GO:0070663 | regulation of leukocyte proliferation | | 23/3240 | 6.02584E-05 |
| GO:0002833 | positive regulation of response to biotic stimulus | | 34/3240 | 6.9578E-05 |
| GO:0043062 | extracellular structure organization | | 49/3240 | 7.22688E-05 |
| GO:0002218 | activation of innate immune response | | 29/3240 | 9.35286E-05 |
| GO:0009117 | nucleotide metabolic process | | 67/3240 | 0.000117233 |
| GO:0045088 | regulation of innate immune response | | 35/3240 | 0.000118688 |
| GO:0022407 | regulation of cell-cell adhesion | | 27/3240 | 0.000144284 |
| GO:0140888 | interferon-mediated signaling pathway | | 12/3240 | 0.000144284 |
| GO:1903037 | regulation of leukocyte cell-cell adhesion | | 23/3240 | 0.000144284 |
| GO:0006457 | protein folding | | 45/3240 | 0.000144284 |
| GO:0045089 | positive regulation of innate immune response | | 32/3240 | 0.000144496 |
| GO:0050776 | regulation of immune response | | 81/3240 | 0.000159544 |
| GO:0060337 | type I interferon-mediated signaling pathway | | 11/3240 | 0.000162201 |
| GO:0071357 | cellular response to type I interferon | | 11/3240 | 0.000162201 |
| GO:0031663 | lipopolysaccharide-mediated signaling pathway | | 9/3240 | 0.000163774 |
| GO:0051851 | host-mediated perturbation of symbiont process | | 10/3240 | 0.00017446 |
| GO:0050863 | regulation of T cell activation | | 25/3240 | 0.000203262 |
| GO:0031349 | positive regulation of defense response | | 34/3240 | 0.000290458 |
| GO:0032496 | response to lipopolysaccharide | | 20/3240 | 0.000302487 |
| GO:0002237 | response to molecule of bacterial origin | | 21/3240 | 0.000311297 |
| GO:0050778 | positive regulation of immune response | | 72/3240 | 0.000329125 |
| GO:0007155 | cell adhesion | | 138/3240 | 0.000356704 |
| GO:0051250 | negative regulation of lymphocyte activation | | 17/3240 | 0.00037093 |
| GO:0002263 | cell activation involved in immune response | | 20/3240 | 0.000469163 |
| GO:0035456 | response to interferon-beta | | 9/3240 | 0.00062404 |
| GO:0046597 | host-mediated suppression of symbiont invasion | | 9/3240 | 0.00062404 |
| GO:0042098 | T cell proliferation | | 17/3240 | 0.00062404 |
| GO:0042129 | regulation of T cell proliferation | | 17/3240 | 0.00062404 |
| GO:0002683 | negative regulation of immune system process | | 28/3240 | 0.000652741 |
| GO:0002695 | negative regulation of leukocyte activation | | 18/3240 | 0.000652741 |
| GO:0050866 | negative regulation of cell activation | | 18/3240 | 0.000652741 |
| GO:0071216 | cellular response to biotic stimulus | | 20/3240 | 0.00068133 |
| GO:0044093 | positive regulation of molecular function | | 89/3240 | 0.00068133 |
| GO:0030155 | regulation of cell adhesion | | 36/3240 | 0.000793313 |
| GO:0019693 | ribose phosphate metabolic process | | 48/3240 | 0.000913754 |
| GO:0002253 | activation of immune response | | 57/3240 | 0.000921153 |
| GO:0071222 | cellular response to lipopolysaccharide | | 17/3240 | 0.00096274 |
| GO:0009259 | ribonucleotide metabolic process | | 46/3240 | 0.00096274 |
| GO:0071219 | cellular response to molecule of bacterial origin | | 18/3240 | 0.000992347 |
| GO:0002366 | leukocyte activation involved in immune response | | 19/3240 | 0.000997206 |
| GO:0048585 | negative regulation of response to stimulus | | 98/3240 | 0.001107309 |
| GO:0006163 | purine nucleotide metabolic process | | 54/3240 | 0.001368296 |
| GO:0050864 | regulation of B cell activation | | 16/3240 | 0.001446125 |
| GO:0002758 | innate immune response-activating signaling pathway | | 25/3240 | 0.001487243 |
| GO:0002224 | toll-like receptor signaling pathway | | 13/3240 | 0.001737891 |
| GO:0030595 | leukocyte chemotaxis | | 22/3240 | 0.001871794 |
| GO:0008219 | cell death | | 110/3240 | 0.001871794 |
| GO:0012501 | programmed cell death | | 110/3240 | 0.001871794 |
| GO:0032945 | negative regulation of mononuclear cell proliferation | | 11/3240 | 0.001871794 |
| GO:0050672 | negative regulation of lymphocyte proliferation | | 11/3240 | 0.001871794 |
| GO:0070664 | negative regulation of leukocyte proliferation | | 11/3240 | 0.001871794 |

**Supplementary Table S6.** *Relevant Differentially Expressed Genes for Consolidation versus Healthy Uninfected Comparisons.*

| **Gene** | **Change** | **log2 F.C.** | **p.adjusted** |
| --- | --- | --- | --- |
| CLEC12B | Increased | 21.566 | 5.21539E-13 |
| ZBP1 | Increased | 6.906 | 1.31062E-11 |
| ISG15 | Increased | 7.689 | 1.31062E-11 |
| OAS1Y | Increased | 5.807 | 1.16066E-10 |
| OAS1Z | Increased | 6.076 | 1.41796E-10 |
| IFIT2 | Increased | 7.649 | 2.49755E-10 |
| MX2 | Increased | 6.481 | 2.49755E-10 |
| MX1 | Increased | 5.933 | 6.02953E-10 |
| EPSTI1 | Increased | 4.148 | 6.99509E-10 |
| IFI44 | Increased | 5.052 | 8.10501E-10 |
| XAF1 | Increased | 4.417 | 9.03662E-10 |
| CLEC4F | Increased | 9.637 | 1.6537E-09 |
| NCF1 | Increased | 4.716 | 1.6537E-09 |
| IFI6 | Increased | 5.388 | 1.75785E-09 |
| OAS2 | Increased | 5.441 | 3.15718E-09 |
| DHX58 | Increased | 5.657 | 5.59446E-09 |
| ISG12(B) | Increased | 7.308 | 8.74925E-09 |
| RSAD2 | Increased | 6.312 | 1.06298E-08 |
| NCF4 | Increased | 3.622 | 1.69935E-08 |
| ISG20 | Increased | 5.638 | 1.98881E-08 |
| IFI44L | Increased | 4.755 | 4.2613E-08 |
| USP18 | Increased | 5.497 | 6.70977E-08 |
| OAS1X | Increased | 4.592 | 9.09734E-08 |
| IFIT3 | Increased | 5.756 | 1.09699E-07 |
| IFIT5 | Increased | 3.417 | 1.19461E-07 |
| IFIH1 | Increased | 3.862 | 1.25588E-07 |
| NLRC4 | Increased | 2.709 | 1.2832E-07 |
| NOD1 | Increased | 3.151 | 2.19512E-07 |
| CGAS | Increased | 3.15 | 4.6792E-07 |
| SIGLEC1 | Increased | 6.715 | 1.29322E-06 |
| IFI16 | Increased | 2.89 | 1.74899E-06 |
| TLR7 | Increased | 4.156 | 2.44759E-06 |
| CMPK2 | Increased | 4.467 | 4.30565E-06 |
| CLEC12A | Increased | 3.669 | 5.7764E-06 |
| CYBB | Increased | 2.366 | 9.85023E-06 |
| NOD2 | Increased | 2.392 | 2.19314E-05 |
| S100A9 | Increased | 3.342 | 2.76486E-05 |
| LCN2 | Increased | 5.463 | 3.5332E-05 |
| TLR2 | Increased | 3.002 | 5.49039E-05 |
| CXCL3 | Increased | 3.409 | 8.00006E-05 |
| CXCL2 | Increased | 5.162 | 8.00006E-05 |
| CLEC4E | Increased | 1.984 | 0.000247279 |
| CXCR2 | Increased | 3.748 | 0.000657483 |
| NLRP3 | Increased | 2.461 | 0.000689706 |
| CXCR1 | Increased | 4.021 | 0.000876188 |
| CLEC4D | Increased | 2.887 | 0.001065884 |
| CXCL5 | Increased | 3.272 | 0.00126845 |
| S100A8 | Increased | 2.593 | 0.001472722 |
| CLEC10A | Increased | 3.633 | 0.001567044 |
| NLRP1 | Increased | 2.471 | 0.002773415 |
| OLFM4 | Increased | 5.375 | 0.010356347 |
| CLEC4A | Increased | 1.721 | 0.011527559 |
| AIM2 | Increased | 3.748 | 0.013355297 |
| CXCL8 | Increased | 3.497 | 0.013589504 |
| S100A12 | Increased | 2.659 | 0.018020758 |
| NLRP12 | Increased | 2.895 | 0.026741223 |
| SAA3 | Increased | 10.704 | 1.87637E-22 |
| M-SAA3.2 | Increased | 9.803 | 1.03104E-15 |
| TREM1 | Increased | 9.018 | 3.38886E-13 |
| FCGR1A | Increased | 4.712 | 3.1779E-12 |
| COL4A5 | Decreased | -4.913 | 5.30708E-10 |
| HP | Increased | 6.242 | 2.24687E-09 |
| TIMP1 | Increased | 6.493 | 8.01636E-09 |
| COL4A3 | Decreased | -5 | 1.31671E-08 |
| COL4A6 | Decreased | -4.55 | 1.97969E-08 |
| C2 | Increased | 4.794 | 5.98867E-08 |
| C5AR2 | Increased | 3.179 | 6.63531E-08 |
| CTSL | Increased | 6.119 | 1.43901E-07 |
| PTX3 | Increased | 5.245 | 2.73194E-07 |
| COL4A4 | Decreased | -3.623 | 5.30579E-06 |
| CD68 | Increased | 3.403 | 9.62397E-06 |
| HIF1A | Increased | 2.858 | 1.0976E-05 |
| MMP25 | Increased | 4.558 | 1.2949E-05 |
| SIRPA | Increased | 2.874 | 2.17603E-05 |
| C5AR1 | Increased | 3.082 | 3.20748E-05 |
| SIRPB1 | Increased | 2.764 | 4.22016E-05 |
| CSF3 | Increased | 5.349 | 5.47754E-05 |
| CSF3R | Increased | 4.56 | 5.6399E-05 |
| EMILIN2 | Increased | 3.506 | 7.15048E-05 |
| CTSS | Increased | 2.092 | 8.24685E-05 |
| LDHA | Increased | 3.245 | 8.76246E-05 |
| AGER | Decreased | -3.56 | 0.000102159 |
| CD163 | Increased | 2.254 | 0.000172577 |
| COL4A2 | Decreased | -2.801 | 0.000239818 |
| HPX | Increased | 5.328 | 0.000295825 |
| CTSK | Increased | 3.262 | 0.000340107 |
| COL2A1 | Decreased | -8.618 | 0.000407214 |
| PCDH20 | Decreased | -4.043 | 0.000502664 |
| SLC2A1 | Increased | 3.111 | 0.00054469 |
| MMP9 | Increased | 6.666 | 0.0005485 |
| SAA2 | Increased | 7.156 | 0.000581446 |
| BPIFA2A | Decreased | -8.432 | 0.000586642 |
| P4HA1 | Increased | 2.113 | 0.000837665 |
| EGLN3 | Increased | 3 | 0.000862343 |
| HAPLN1 | Decreased | -5.027 | 0.001344389 |
| BNIP3 | Increased | 2.819 | 0.001394794 |
| VCAN | Increased | 2.838 | 0.00163004 |
| CSF1R | Increased | 1.999 | 0.001903709 |
| HK2 | Increased | 1.829 | 0.002929651 |
| COL9A3 | Decreased | -3.992 | 0.00395137 |
| COL9A2 | Decreased | -3.972 | 0.004020591 |
| TREM2 | Increased | 2.839 | 0.004328201 |
| FREM3 | Decreased | -4.388 | 0.004328201 |
| SIRPB2 | Increased | 1.998 | 0.00459037 |
| AQP5 | Decreased | -2.185 | 0.00532828 |
| FREM2 | Decreased | -3.424 | 0.005567491 |
| MXRA5 | Increased | 2.961 | 0.007184133 |
| LBP | Increased | 4.723 | 0.008507226 |
| SLC2A3 | Increased | 2.483 | 0.009438529 |
| PLOD1 | Increased | 1.669 | 0.00970143 |
| SFTPC | Decreased | -2.745 | 0.022156718 |
| FCGR2B | Increased | 1.559 | 0.022255499 |
| MMP1 | Increased | 5.189 | 0.026502823 |
| COL9A1 | Decreased | -4.912 | 0.043960054 |
| HOXA3 | Decreased | -2.783 | 0.000138382 |
| BDNF | Decreased | -2.814 | 0.00047073 |
| FOXO4 | Decreased | -2.271 | 0.000657385 |
| ADCY5 | Decreased | -3.38 | 0.001029021 |
| HOXA5 | Decreased | -2.11 | 0.001617777 |
| SOX17 | Decreased | -3.091 | 0.002494805 |
| DSC2 | Decreased | -2.182 | 0.002974663 |
| NTRK2 | Decreased | -3.012 | 0.003370259 |
| SOX7 | Decreased | -2.57 | 0.004264795 |
| FOXO6 | Decreased | -3.647 | 0.004378175 |
| STMN2 | Decreased | -7.962 | 0.008721434 |
| KRT79 | Decreased | -4.114 | 0.027779111 |
| GRIN2B | Decreased | -5.164 | 0.030238535 |
| CTSH | Increased | 3.217 | 9.83004E-09 |
| ZBP1 | Increased | 6.906 | 1.31062E-11 |

**Supplementary Table S7**. *Enriched GO Terms for Grey Hepatization versus Healthy Uninfected Comparisons.*

| **ID** | **Description** | **GeneRatio** | **p.adjust** |  |
| --- | --- | --- | --- | --- |
| GO:0009615 | response to virus | 41/4157 | 4.16395E-12 |  |
| GO:0051607 | defense response to virus | 39/4157 | 1.9638E-11 |  |
| GO:0002682 | regulation of immune system process | 159/4157 | 1.19546E-10 |  |
| GO:0002684 | positive regulation of immune system process | 132/4157 | 1.87121E-10 |  |
| GO:0034097 | response to cytokine | 84/4157 | 2.46984E-09 |  |
| GO:1901652 | response to peptide | 84/4157 | 2.46984E-09 |  |
| GO:0031347 | regulation of defense response | 72/4157 | 2.71982E-09 |  |
| GO:0002218 | activation of innate immune response | 41/4157 | 8.96663E-09 |  |
| GO:0002831 | regulation of response to biotic stimulus | 52/4157 | 1.07534E-08 |  |
| GO:0032101 | regulation of response to external stimulus | 90/4157 | 1.31239E-08 |  |
| GO:0002833 | positive regulation of response to biotic stimulus | 47/4157 | 1.5664E-08 |  |
| GO:0045088 | regulation of innate immune response | 49/4157 | 1.66673E-08 |  |
| GO:0044419 | biological process involved in interspecies interaction between organisms | 152/4157 | 1.66673E-08 |  |
| GO:0002221 | pattern recognition receptor signaling pathway | 30/4157 | 1.78787E-08 |  |
| GO:0045089 | positive regulation of innate immune response | 45/4157 | 1.81442E-08 |  |
| GO:0071345 | cellular response to cytokine stimulus | 74/4157 | 2.25527E-08 |  |
| GO:0009607 | response to biotic stimulus | 146/4157 | 4.40744E-08 |  |
| GO:0007155 | cell adhesion | 188/4157 | 6.77794E-08 |  |
| GO:0001775 | cell activation | 83/4157 | 9.21122E-08 |  |
| GO:0043207 | response to external biotic stimulus | 144/4157 | 9.21122E-08 |  |
| GO:0051707 | response to other organism | 144/4157 | 9.21122E-08 |  |
| GO:0046649 | lymphocyte activation | 64/4157 | 1.15317E-07 |  |
| GO:0006954 | inflammatory response | 71/4157 | 1.19086E-07 |  |
| GO:0002758 | innate immune response-activating signaling pathway | 37/4157 | 1.27315E-07 |  |
| GO:0045321 | leukocyte activation | 73/4157 | 1.27421E-07 |  |
| GO:0031349 | positive regulation of defense response | 47/4157 | 1.27421E-07 |  |
| GO:0051249 | regulation of lymphocyte activation | 47/4157 | 1.27421E-07 |  |
| GO:0098609 | cell-cell adhesion | 130/4157 | 1.64572E-07 |  |
| GO:0019221 | cytokine-mediated signaling pathway | 67/4157 | 1.94601E-07 |  |
| GO:0032103 | positive regulation of response to external stimulus | 56/4157 | 3.2557E-07 |  |
| GO:0098542 | defense response to other organism | 102/4157 | 4.06822E-07 |  |
| GO:0050790 | regulation of catalytic activity | 169/4157 | 4.73819E-07 |  |
| GO:0050865 | regulation of cell activation | 54/4157 | 5.14731E-07 |  |
| GO:0050776 | regulation of immune response | 106/4157 | 7.77338E-07 |  |
| GO:0032944 | regulation of mononuclear cell proliferation | 29/4157 | 7.77338E-07 |  |
| GO:0050670 | regulation of lymphocyte proliferation | 29/4157 | 7.77338E-07 |  |
| GO:0070663 | regulation of leukocyte proliferation | 29/4157 | 7.77338E-07 |  |
| GO:0002694 | regulation of leukocyte activation | 52/4157 | 7.77338E-07 |  |
| GO:1902531 | regulation of intracellular signal transduction | 164/4157 | 8.058E-07 |  |
| GO:0002253 | activation of immune response | 78/4157 | 9.44815E-07 |  |
| GO:0045087 | innate immune response | 92/4157 | 1.04928E-06 |  |
| GO:0050778 | positive regulation of immune response | 95/4157 | 1.40009E-06 |  |
| GO:0140546 | defense response to symbiont | 99/4157 | 1.4061E-06 |  |
| GO:0051239 | regulation of multicellular organismal process | 168/4157 | 2.20367E-06 |  |
| GO:0032943 | mononuclear cell proliferation | 30/4157 | 2.35123E-06 |  |
| GO:0046651 | lymphocyte proliferation | 30/4157 | 2.35123E-06 |  |
| GO:0070661 | leukocyte proliferation | 30/4157 | 2.35123E-06 |  |
| GO:0001525 | angiogenesis | 41/4157 | 2.96666E-06 |  |
| GO:0042110 | T cell activation | 40/4157 | 3.80225E-06 |  |
| GO:0002757 | immune response-activating signaling pathway | 61/4157 | 3.89419E-06 |  |
| GO:0080134 | regulation of response to stress | 87/4157 | 4.06168E-06 |  |
| GO:0030036 | actin cytoskeleton organization | 137/4157 | 4.48066E-06 |  |
| GO:0002764 | immune response-regulating signaling pathway | 62/4157 | 5.60663E-06 |  |
| GO:0007159 | leukocyte cell-cell adhesion | 32/4157 | 9.16773E-06 |  |
| GO:0030029 | actin filament-based process | 139/4157 | 1.46832E-05 |  |
| GO:0140888 | interferon-mediated signaling pathway | 14/4157 | 1.70639E-05 |  |
| GO:0048585 | negative regulation of response to stimulus | 128/4157 | 1.70639E-05 |  |
| GO:0048514 | blood vessel morphogenesis | 42/4157 | 1.99679E-05 |  |
| GO:0051604 | protein maturation | 107/4157 | 2.44116E-05 |  |
| GO:0030198 | extracellular matrix organization | 58/4157 | 2.8375E-05 |  |
| GO:0045229 | external encapsulating structure organization | 58/4157 | 2.8375E-05 |  |
| GO:0001944 | vasculature development | 45/4157 | 2.8375E-05 |  |
| GO:0002683 | negative regulation of immune system process | 35/4157 | 5.06381E-05 |  |
| GO:0034340 | response to type I interferon | 13/4157 | 5.06381E-05 |  |
| GO:0043062 | extracellular structure organization | 58/4157 | 5.06381E-05 |  |
| GO:0051251 | positive regulation of lymphocyte activation | 29/4157 | 5.18742E-05 |  |
| GO:0043086 | negative regulation of catalytic activity | 61/4157 | 5.48447E-05 |  |
| GO:0072359 | circulatory system development | 69/4157 | 6.35089E-05 |  |
| GO:0045069 | regulation of viral genome replication | 15/4157 | 6.56184E-05 |  |
| GO:0045071 | negative regulation of viral genome replication | 15/4157 | 6.56184E-05 |  |
| GO:0019220 | regulation of phosphate metabolic process | 104/4157 | 7.34791E-05 |  |
| GO:0051174 | regulation of phosphorus metabolic process | 104/4157 | 7.34791E-05 |  |
| GO:0001568 | blood vessel development | 42/4157 | 8.30277E-05 |  |
| GO:0043549 | regulation of kinase activity | 71/4157 | 9.96805E-05 |  |
| GO:0042325 | regulation of phosphorylation | 97/4157 | 0.000104308 |  |
| GO:0009968 | negative regulation of signal transduction | 101/4157 | 0.000136928 |  |
| GO:0060337 | type I interferon-mediated signaling pathway | 12/4157 | 0.00014147 |  |
| GO:0071357 | cellular response to type I interferon | 12/4157 | 0.00014147 |  |
| GO:0009967 | positive regulation of signal transduction | 112/4157 | 0.000160422 |  |
| GO:0035239 | tube morphogenesis | 42/4157 | 0.000161788 |  |
| GO:0048525 | negative regulation of viral process | 15/4157 | 0.000172317 |  |
| GO:0002696 | positive regulation of leukocyte activation | 33/4157 | 0.000172871 |  |
| GO:0050867 | positive regulation of cell activation | 33/4157 | 0.000172871 |  |
| GO:0030155 | regulation of cell adhesion | 44/4157 | 0.000178951 |  |
| GO:0032496 | response to lipopolysaccharide | 23/4157 | 0.000178951 |  |
| GO:0048646 | anatomical structure formation involved in morphogenesis | 65/4157 | 0.000178951 |  |
| GO:0042098 | T cell proliferation | 20/4157 | 0.000178951 |  |
| GO:0042129 | regulation of T cell proliferation | 20/4157 | 0.000178951 |  |
| GO:0051338 | regulation of transferase activity | 72/4157 | 0.000220185 |  |
| GO:0002237 | response to molecule of bacterial origin | 24/4157 | 0.000246709 |  |
| GO:0010648 | negative regulation of cell communication | 101/4157 | 0.000352347 |  |
| GO:0022407 | regulation of cell-cell adhesion | 30/4157 | 0.000361914 |  |
| GO:0050863 | regulation of T cell activation | 28/4157 | 0.00039082 |  |
| GO:0062207 | regulation of pattern recognition receptor signaling pathway | 11/4157 | 0.00039082 |  |
| GO:0019079 | viral genome replication | 15/4157 | 0.00039082 |  |
| GO:0023057 | negative regulation of signaling | 101/4157 | 0.000399757 |  |
| GO:1903900 | regulation of viral life cycle | 19/4157 | 0.00041081 |  |
| GO:0035295 | tube development | 44/4157 | 0.000426143 |  |
| GO:0007169 | cell surface receptor protein tyrosine kinase signaling pathway | 89/4157 | 0.00048274 |  |
| GO:1903037 | regulation of leukocyte cell-cell adhesion | 25/4157 | 0.000505712 |  |

**Supplementary Table S8.** *Discussion Relevant Differentially Expressed Genes for Grey Hepatization versus Healthy Uninfected Comparisons.*

| **Gene** | **Change** | **log2 F.C.** | **p.adjusted** |
| --- | --- | --- | --- |
| OAS1Z | Increased | 5.992 | 1.33E-10 |
| ZBP1 | Increased | 6.156 | 1.24E-09 |
| ISG12(B) | Increased | 7.544 | 1.70E-09 |
| IFIT2 | Increased | 6.979 | 5.62E-09 |
| MX2 | Increased | 5.862 | 7.97E-09 |
| OAS1Y | Increased | 5.095 | 1.29E-08 |
| MX1 | Increased | 5.313 | 2.32E-08 |
| IFITM2 | Increased | 4.869 | 6.47E-08 |
| OAS2 | Increased | 4.91 | 7.03E-08 |
| EPSTI1 | Increased | 3.576 | 8.82E-08 |
| DHX58 | Increased | 5.103 | 1.17E-07 |
| XAF1 | Increased | 3.772 | 1.47E-07 |
| IFITM1 | Increased | 4.512 | 2.55E-07 |
| TLR7 | Increased | 4.36 | 3.50E-07 |
| OAS1X | Increased | 4.246 | 5.05E-07 |
| RSAD2 | Increased | 5.314 | 1.33E-06 |
| IFIT3 | Increased | 5.015 | 2.66E-06 |
| TLR2 | Increased | 3.354 | 2.68E-06 |
| IFIH1 | Increased | 3.347 | 3.43E-06 |
| USP18 | Increased | 4.552 | 6.22E-06 |
| CGAS | Increased | 2.449 | 7.61E-05 |
| NLRP12 | Increased | 4.061 | 6.89E-04 |
| CMPK2 | Increased | 2.942 | 2.53E-03 |
| AIM2 | Increased | 4.053 | 4.78E-03 |
| IFITM10 | Increased | 4.196 | 3.49E-02 |
| TNIP3 | Increased | 7.626 | 5.09E-12 |
| NCF1 | Increased | 4.975 | 9.99E-11 |
| LCN2 | Increased | 7.034 | 2.59E-08 |
| NCF4 | Increased | 3.479 | 4.34E-08 |
| S100A9 | Increased | 4.113 | 6.96E-08 |
| CYBB | Increased | 2.672 | 2.16E-07 |
| DDIT4 | Increased | 3.771 | 2.15E-06 |
| CXCL2 | Increased | 5.889 | 2.72E-06 |
| CXCR2 | Increased | 4.646 | 8.43E-06 |
| S100A8 | Increased | 3.386 | 1.04E-05 |
| CXCL5 | Increased | 4.114 | 1.70E-05 |
| OLFM4 | Increased | 8.247 | 1.77E-05 |
| TNIP1 | Increased | 2.798 | 2.71E-05 |
| CXCR1 | Increased | 4.742 | 3.50E-05 |
| S100A12 | Increased | 4.151 | 4.90E-05 |
| CXCL8 | Increased | 5.08 | 9.46E-05 |
| SOCS1 | Increased | 3.958 | 1.58E-04 |
| NFKBIA | Increased | 2.174 | 6.34E-04 |
| TNFAIP3 | Increased | 2.301 | 9.42E-04 |
| SOCS3 | Increased | 2.705 | 1.12E-03 |
| IKBKE | Increased | 2.283 | 9.74E-03 |
| CXCL3 | Increased | 2.063 | 1.94E-02 |
| SAA3 | Increased | 11.188 | 2.39E-25 |
| M-SAA3.2 | Increased | 9.814 | 4.16E-16 |
| TREM1 | Increased | 9.261 | 1.70E-14 |
| FCGR1A | Increased | 5.047 | 1.83E-14 |
| OSMR | Increased | 3.04 | 1.49E-13 |
| HP | Increased | 6.293 | 1.04E-09 |
| C5AR2 | Increased | 3.494 | 1.26E-09 |
| TYROBP | Increased | 2.785 | 1.39E-07 |
| SIRPB1 | Increased | 3.352 | 2.23E-07 |
| PTX3 | Increased | 5.164 | 2.42E-07 |
| C2 | Increased | 4.478 | 2.76E-07 |
| CSF3R | Increased | 5.561 | 2.94E-07 |
| C5AR1 | Increased | 3.572 | 5.05E-07 |
| CD68 | Increased | 3.718 | 5.06E-07 |
| SIRPA | Increased | 2.962 | 5.72E-06 |
| HPX | Increased | 5.694 | 5.54E-05 |
| TREM2 | Increased | 3.546 | 1.43E-04 |
| CD163 | Increased | 2.192 | 1.57E-04 |
| CSF1R | Increased | 2.118 | 5.77E-04 |
| LBP | Increased | 5.748 | 6.20E-04 |
| FCGR2B | Increased | 2.051 | 1.12E-03 |
| SAA2 | Increased | 6.559 | 1.24E-03 |
| FCGR2A | Increased | 2.032 | 2.61E-03 |
| C9 | Increased | 8.299 | 5.90E-03 |
| CTSL | Increased | 7.145 | 2.60E-10 |
| CTSH | Increased | 3.233 | 5.28E-09 |
| TIMP1 | Increased | 6.445 | 6.74E-09 |
| MMP25 | Increased | 5.137 | 3.30E-07 |
| P4HA1 | Increased | 2.897 | 1.18E-06 |
| CTSK | Increased | 3.959 | 4.78E-06 |
| EMILIN2 | Increased | 3.846 | 5.77E-06 |
| MMP9 | Increased | 8.001 | 1.28E-05 |
| MEDAG | Increased | 2.802 | 1.69E-05 |
| VCAN | Increased | 3.409 | 6.21E-05 |
| MMP1 | Increased | 8.425 | 6.79E-05 |
| MMP14 | Increased | 2.747 | 6.82E-05 |
| SULF1 | Increased | 3.974 | 1.02E-04 |
| CTSZ | Increased | 2.613 | 1.66E-04 |
| PLOD2 | Increased | 4.192 | 1.69E-04 |
| PLOD1 | Increased | 2.037 | 7.28E-04 |
| MXRA5 | Increased | 3.487 | 7.42E-04 |
| FAP | Increased | 4.404 | 1.66E-03 |
| MMP13 | Increased | 4.25 | 2.69E-03 |
| TGFBI | Increased | 2.065 | 3.19E-03 |
| CTHRC1 | Increased | 3.466 | 4.49E-02 |
| LDHA | Increased | 4.288 | 5.51E-08 |
| HIF1A | Increased | 3.206 | 3.04E-07 |
| HSPE1 | Increased | 2.51 | 1.19E-06 |
| SEMA4D | Increased | 3.937 | 1.53E-06 |
| BNIP3 | Increased | 3.852 | 3.30E-06 |
| VCAM1 | Increased | 3.031 | 7.41E-06 |
| SLC2A1 | Increased | 3.809 | 8.21E-06 |
| HSPH1 | Increased | 3.163 | 9.66E-06 |
| PROK2 | Increased | 5.412 | 1.04E-05 |
| EGLN3 | Increased | 3.721 | 1.24E-05 |
| GDF10 | Decreased | -4.657 | 1.63E-05 |
| ENO1 | Increased | 2.859 | 2.82E-05 |
| NID2 | Increased | 2.351 | 3.95E-05 |
| SESN2 | Increased | 3.265 | 5.54E-05 |
| SLC2A3 | Increased | 3.522 | 6.50E-05 |
| HK2 | Increased | 2.227 | 1.18E-04 |
| IL21R | Increased | 3.452 | 1.87E-04 |
| RASA4B | Increased | 3.284 | 1.99E-04 |
| DDIT3 | Increased | 1.854 | 3.37E-04 |
| FGF23 | Increased | 6.081 | 5.41E-04 |
| ALDOC | Increased | 2.55 | 5.54E-04 |
| CREB3L1 | Increased | 3.292 | 1.00E-03 |
| GDF15 | Increased | 3.769 | 1.01E-02 |
| THBS2 | Increased | 3.307 | 1.95E-02 |
| HSPA1A | Increased | 2.264 | 2.11E-02 |
| ETV3L | Increased | 4.382 | 3.29E-02 |
| SEMA3A | Increased | 2.038 | 4.24E-02 |
| IL11 | Increased | 2.806 | 4.55E-02 |
| GUCY2D | Increased | 3.768 | 4.60E-02 |
| AGER | Decreased | -5.074 | 5.42E-09 |
| AQP5 | Decreased | -3.465 | 1.69E-06 |
| CLDN5 | Decreased | -4.135 | 1.98E-06 |
| SLC22A31 | Decreased | -3.977 | 2.07E-06 |
| SLC16A11 | Decreased | -2.347 | 5.99E-05 |
| SLC45A1 | Decreased | -5.154 | 9.20E-05 |
| SFTPC | Decreased | -4.157 | 1.43E-04 |
| BPIFA2A | Decreased | -8.509 | 3.06E-04 |
| SCGB1D | Decreased | -5.189 | 3.98E-04 |
| SCNN1B | Decreased | -3.461 | 1.31E-03 |
| SPINK5 | Decreased | -4.55 | 6.98E-03 |
| CLDN18 | Decreased | -2.185 | 7.08E-03 |
| SLC34A2 | Decreased | -2.143 | 8.49E-03 |
| SLC5A9 | Decreased | -2.598 | 9.38E-03 |
| SLC22A17 | Decreased | -2.075 | 1.43E-02 |
| SLC9A4 | Decreased | -4.186 | 2.66E-02 |
| MUC4 | Decreased | -4.982 | 3.09E-02 |
| SCGB2A2 | Unchanged | -1.544 | 1.80E-01 |
| COL4A5 | Decreased | -5.778 | 4.50E-14 |
| COL4A3 | Decreased | -5.932 | 5.09E-12 |
| SPOCK2 | Decreased | -3.947 | 1.25E-10 |
| MXRA7 | Decreased | -3.944 | 5.45E-08 |
| COL25A1 | Decreased | -4.557 | 1.43E-06 |
| COL27A1 | Decreased | -3.284 | 3.74E-06 |
| COL15A1 | Decreased | -3.785 | 1.12E-05 |
| COL21A1 | Decreased | -3.781 | 1.83E-05 |
| WFDC1 | Decreased | -3.144 | 2.76E-05 |
| COL13A1 | Decreased | -4.508 | 3.27E-05 |
| WFIKKN2 | Decreased | -5.761 | 6.34E-05 |
| FREM2 | Decreased | -4.564 | 9.12E-05 |
| EMID1 | Decreased | -2.819 | 2.23E-04 |
| COL26A1 | Decreased | -3.718 | 2.42E-04 |
| COL6A6 | Decreased | -4.642 | 2.67E-04 |
| HMCN2 | Decreased | -4.3 | 5.22E-04 |
| COL24A1 | Decreased | -4.247 | 9.23E-04 |
| COL2A1 | Decreased | -7.68 | 9.69E-04 |
| HMCN1 | Decreased | -2.878 | 1.90E-03 |
| FREM1 | Decreased | -2.532 | 2.53E-03 |
| SPOCK1 | Decreased | -2.823 | 2.81E-03 |
| COL9A3 | Decreased | -3.958 | 2.91E-03 |
| COL9A2 | Decreased | -3.907 | 3.21E-03 |
| COL9A1 | Decreased | -6.78 | 3.26E-03 |
| COL4A1 | Decreased | -2.082 | 3.35E-03 |
| COL5A3 | Decreased | -2.233 | 4.17E-03 |
| FREM3 | Decreased | -4.175 | 4.76E-03 |
| COL11A2 | Decreased | -3.53 | 7.39E-03 |
| COLQ | Decreased | -3.964 | 2.07E-02 |
| PRELP | Decreased | -2.073 | 4.52E-02 |
| BMP5 | Decreased | -4.328 | 1.67E-09 |
| BMP6 | Decreased | -4.018 | 9.37E-07 |
| TBX2 | Decreased | -3.446 | 9.73E-07 |
| FGFR3 | Decreased | -4.063 | 1.61E-06 |
| HOXA5 | Decreased | -2.927 | 3.34E-06 |
| PRDM6 | Decreased | -3.152 | 5.72E-06 |
| FGFR4 | Decreased | -4.553 | 1.22E-05 |
| SOX7 | Decreased | -3.604 | 1.67E-05 |
| SOX17 | Decreased | -3.978 | 3.97E-05 |
| HOXA3 | Decreased | -2.883 | 4.13E-05 |
| NKD1 | Decreased | -3.248 | 7.96E-05 |
| SOX4 | Decreased | -2.626 | 8.00E-05 |
| WNT7A | Decreased | -3.654 | 8.64E-05 |
| TCF21 | Decreased | -3.15 | 8.77E-05 |
| WNT9A | Decreased | -3.238 | 1.72E-04 |
| WNT11 | Decreased | -3.838 | 1.79E-04 |
| FGFR1 | Decreased | -2.113 | 1.85E-04 |
| AXIN2 | Decreased | -3.191 | 1.87E-04 |
| GATA6 | Decreased | -2.029 | 3.95E-04 |
| HOXB3 | Decreased | -2.35 | 4.60E-04 |
| TBX3 | Decreased | -2.46 | 5.37E-04 |
| IRX3 | Decreased | -2.107 | 6.81E-04 |
| IRX5 | Decreased | -2.118 | 8.68E-04 |
| GATA5 | Decreased | -3.266 | 8.81E-04 |
| FGFR2 | Decreased | -2.311 | 9.49E-04 |
| GATA3 | Decreased | -2.269 | 1.01E-03 |
| TBX4 | Decreased | -2.365 | 1.14E-03 |
| BMPER | Decreased | -2.426 | 1.63E-03 |
| IRX2 | Decreased | -2.046 | 1.81E-03 |
| HOXB5 | Decreased | -2.559 | 2.18E-03 |
| IRX1 | Decreased | -2.45 | 2.31E-03 |
| PRDM16 | Decreased | -2.605 | 2.33E-03 |
| NKD2 | Decreased | -2.561 | 2.36E-03 |
| FGF1 | Decreased | -2.108 | 4.86E-03 |
| HOXA2 | Decreased | -2.552 | 6.50E-03 |
| FGF12 | Decreased | -2.402 | 7.67E-03 |
| SOX13 | Decreased | -2.322 | 8.51E-03 |
| HOXB6 | Decreased | -2.214 | 9.08E-03 |
| WNT2 | Decreased | -2.163 | 1.27E-02 |
| WNT8B | Decreased | -3.35 | 2.32E-02 |
| NOTUM | Decreased | -3.725 | 2.52E-02 |
| TBX1 | Decreased | -2.099 | 3.33E-02 |
| FGF20 | Decreased | -3.244 | 3.95E-02 |
| GPRASP1 | Decreased | -3.365 | 9.88E-09 |
| GPRASP2 | Decreased | -2.492 | 1.29E-08 |
| CNR1 | Decreased | -7.277 | 1.25E-07 |
| RIMS3 | Decreased | -3.683 | 7.66E-07 |
| ADCY5 | Decreased | -4.772 | 1.98E-06 |
| GRIN3A | Decreased | -4.358 | 1.09E-05 |
| CNR2 | Decreased | -3.023 | 3.60E-05 |
| GPR37 | Decreased | -4.5 | 5.80E-05 |
| PDYN | Decreased | -4.913 | 5.54E-04 |
| CHRNB3 | Decreased | -5.183 | 6.73E-04 |
| SYNDIG1L | Decreased | -2.49 | 7.87E-04 |
| ADCYAP1R1 | Decreased | -3.727 | 8.00E-04 |
| GPR55 | Decreased | -3.381 | 8.98E-04 |
| GAP43 | Decreased | -6.838 | 1.40E-03 |
| STMN2 | Decreased | -8.574 | 2.92E-03 |
| RIMS1 | Decreased | -3.918 | 3.05E-03 |
| SYT13 | Decreased | -6.516 | 5.17E-03 |
| GRIN1 | Decreased | -2.519 | 8.72E-03 |
| NTM | Decreased | -7.39 | 1.38E-02 |
| GRIN2B | Decreased | -5.462 | 1.55E-02 |
| SYT7 | Decreased | -2.243 | 2.29E-02 |
| SYT8 | Decreased | -4.376 | 2.53E-02 |
| GALR3 | Decreased | -2.885 | 3.23E-02 |
| CRHR2 | Decreased | -3.895 | 3.28E-02 |
| SCGN | Decreased | -4.119 | 3.63E-02 |
| CHRNB4 | Decreased | -4.041 | 4.99E-02 |
| GSTA3 | Decreased | -7.536 | 3.54E-08 |
| CYP1A1 | Decreased | -9.384 | 6.84E-08 |
| GSTT2 | Decreased | -4.433 | 1.44E-07 |
| ABCC6 | Decreased | -4.355 | 4.87E-07 |
| TRPV4 | Decreased | -3.006 | 9.87E-07 |
| AQP5 | Decreased | -3.465 | 1.69E-06 |
| SLC22A31 | Decreased | -3.977 | 2.07E-06 |
| CYP2A13 | Decreased | -6.476 | 2.75E-06 |
| TRPC3 | Decreased | -3.529 | 1.06E-05 |
| CNGA2 | Decreased | -5.899 | 1.17E-05 |
| HPGD | Decreased | -4.564 | 1.69E-05 |
| UGT1A6 | Decreased | -7.163 | 1.89E-05 |
| TRPM4 | Decreased | -3.236 | 3.10E-05 |
| CYP4B1 | Decreased | -3.892 | 4.72E-05 |
| GSTA2 | Decreased | -4.298 | 1.06E-04 |
| ABCB1 | Decreased | -3.11 | 2.77E-04 |
| CYP2C90 | Decreased | -4.468 | 5.50E-04 |
| SLC16A9 | Decreased | -3.301 | 8.26E-04 |
| GSTA1 | Decreased | -3.876 | 9.13E-04 |
| P2RX1 | Decreased | -2.708 | 1.18E-03 |
| KCNB2 | Decreased | -3.437 | 1.48E-03 |
| KCNK3 | Decreased | -4.047 | 4.61E-03 |
| KCND3 | Decreased | -3.373 | 6.57E-03 |
| P2RX5 | Decreased | -1.484 | 7.34E-03 |
| AQP1 | Decreased | -2.087 | 8.38E-03 |
| UGT2A1 | Decreased | -3.721 | 8.84E-03 |
| GSTM3 | Decreased | -2.206 | 9.05E-03 |
| SLC5A9 | Decreased | -2.598 | 9.38E-03 |
| SCN11A | Decreased | -5.913 | 9.50E-03 |
| KCNH2 | Decreased | -2.998 | 9.71E-03 |
| CYP4A22 | Decreased | -6.401 | 1.31E-02 |
| CYP17A1 | Decreased | -2.331 | 1.38E-02 |
| SLC24A3 | Decreased | -1.91 | 1.80E-02 |
| SCN3B | Decreased | -3.378 | 1.95E-02 |
| SLC6A1 | Decreased | -2.808 | 2.05E-02 |
| FMO5 | Decreased | -1.259 | 2.31E-02 |
| SLC8A3 | Decreased | -2.804 | 2.40E-02 |
| ASIC2 | Decreased | -3.12 | 3.25E-02 |
| CYP2C87 | Decreased | -4.292 | 3.47E-02 |
| ASIC3 | Decreased | -3.276 | 4.36E-02 |
| CYP1A2 | Decreased | -4.603 | 5.94E-02 |
| CCR1 | Increased | 4.995 | 1.04E-08 |
| SPI1 | Increased | 3.439 | 6.00E-08 |
| IL7R | Increased | 5.065 | 7.74E-08 |
| CD69 | Increased | 4.314 | 1.60E-05 |
| POU2F2 | Increased | 3.219 | 2.53E-05 |
| CCR2 | Increased | 2.785 | 3.27E-05 |
| IL23R | Increased | 4.004 | 3.95E-05 |
| CCR5 | Increased | 3.006 | 6.02E-05 |
| PRDM1 | Increased | 2.132 | 1.22E-04 |
| CD274 | Increased | 3.27 | 2.43E-04 |
| CTLA4 | Increased | 2.384 | 8.98E-04 |
| LAG3 | Increased | 3.118 | 4.81E-03 |
| BATF | Increased | 2.476 | 5.66E-03 |
| CD1B | Decreased | -4.734 | 5.75E-03 |
| CXCL10 | Increased | 2.351 | 1.33E-02 |
| BOLA-DQA2 | Decreased | -7.688 | 4.40E-02 |
| CD1A | Decreased | -3.024 | 5.33E-02 |
| BOLA-DQA5 | Decreased | -3.644 | 1.11E-01 |
| EMILIN3 | Decreased | -10.069 | 1.25E-10 |
| CYM | Decreased | -12.256 | 3.55E-07 |
| CLEC12B | Increased | 16.459 | 9.12E-08 |

**Supplementary Table S9**. *Enriched GO Terms for Necrosis/Sequestra versus Healthy Uninfected Comparisons.*

| **ID** | **Description** | **GeneRatio** | **p.adjust** |  |  |
| --- | --- | --- | --- | --- | --- |
| GO:0007155 | cell adhesion | 295/7188 | 5.84824E-09 |  |  |
| GO:0030036 | actin cytoskeleton organization | 221/7188 | 3.35736E-08 |  |  |
| GO:0030029 | actin filament-based process | 226/7188 | 8.42582E-08 |  |  |
| GO:0098609 | cell-cell adhesion | 196/7188 | 2.56612E-07 |  |  |
| GO:0051607 | defense response to virus | 43/7188 | 6.53806E-07 |  |  |
| GO:0022008 | neurogenesis | 273/7188 | 6.53806E-07 |  |  |
| GO:0009615 | response to virus | 44/7188 | 1.00697E-06 |  |  |
| GO:0048646 | anatomical structure formation involved in morphogenesis | 103/7188 | 5.9788E-06 |  |  |
| GO:0048699 | generation of neurons | 241/7188 | 2.13814E-05 |  |  |
| GO:0030182 | neuron differentiation | 227/7188 | 2.21367E-05 |  |  |
| GO:0001944 | vasculature development | 65/7188 | 2.2592E-05 |  |  |
| GO:0032535 | regulation of cellular component size | 79/7188 | 3.1339E-05 |  |  |
| GO:0034097 | response to cytokine | 107/7188 | 3.42487E-05 |  |  |
| GO:1901652 | response to peptide | 107/7188 | 3.42487E-05 |  |  |
| GO:0072359 | circulatory system development | 104/7188 | 3.8905E-05 |  |  |
| GO:0022603 | regulation of anatomical structure morphogenesis | 93/7188 | 3.8905E-05 |  |  |
| GO:0051239 | regulation of multicellular organismal process | 254/7188 | 4.48117E-05 |  |  |
| GO:0071345 | cellular response to cytokine stimulus | 96/7188 | 4.51462E-05 |  |  |
| GO:0019221 | cytokine-mediated signaling pathway | 89/7188 | 6.08328E-05 |  |  |
| GO:0051241 | negative regulation of multicellular organismal process | 75/7188 | 6.16372E-05 |  |  |
| GO:0000902 | cell morphogenesis | 170/7188 | 6.42577E-05 | |  |
| GO:0048514 | blood vessel morphogenesis | 58/7188 | 8.49715E-05 |  | |
| GO:0120035 | regulation of plasma membrane bounded cell projection organization | 84/7188 | 8.49715E-05 |  | |
| GO:0050790 | regulation of catalytic activity | 248/7188 | 0.000106328 | |  |
| GO:0001568 | blood vessel development | 60/7188 | 0.000110489 |  | |
| GO:0051128 | regulation of cellular component organization | 267/7188 | 0.00011775 | |  |
| GO:0001775 | cell activation | 110/7188 | 0.00011775 | |  |
| GO:0090066 | regulation of anatomical structure size | 97/7188 | 0.000123044 |  | |
| GO:0048638 | regulation of developmental growth | 32/7188 | 0.000150714 |  | |
| GO:0007015 | actin filament organization | 139/7188 | 0.00015188 | |  |
| GO:0016477 | cell migration | 203/7188 | 0.000152573 | |  |
| GO:0010975 | regulation of neuron projection development | 60/7188 | 0.000161376 |  | |
| GO:0045926 | negative regulation of growth | 26/7188 | 0.000162415 |  | |
| GO:0031344 | regulation of cell projection organization | 85/7188 | 0.000162415 |  | |
| GO:0001525 | angiogenesis | 53/7188 | 0.000162415 |  | |
| GO:0008154 | actin polymerization or depolymerization | 63/7188 | 0.000219426 |  | |
| GO:0048666 | neuron development | 180/7188 | 0.000241426 | |  |
| GO:0035239 | tube morphogenesis | 60/7188 | 0.000247561 |  | |
| GO:0048640 | negative regulation of developmental growth | 23/7188 | 0.000266566 |  | |
| GO:0098660 | inorganic ion transmembrane transport | 243/7188 | 0.000309814 | |  |
| GO:0045069 | regulation of viral genome replication | 18/7188 | 0.000309814 |  | |
| GO:0045071 | negative regulation of viral genome replication | 18/7188 | 0.000309814 |  | |
| GO:0098662 | inorganic cation transmembrane transport | 221/7188 | 0.000309814 | |  |
| GO:0048585 | negative regulation of response to stimulus | 190/7188 | 0.000361487 | |  |
| GO:1902531 | regulation of intracellular signal transduction | 238/7188 | 0.000361487 | |  |
| GO:0001558 | regulation of cell growth | 33/7188 | 0.00039646 |  | |
| GO:0032943 | mononuclear cell proliferation | 36/7188 | 0.000408748 |  | |
| GO:0046651 | lymphocyte proliferation | 36/7188 | 0.000408748 |  | |
| GO:0070661 | leukocyte proliferation | 36/7188 | 0.000408748 |  | |
| GO:0046649 | lymphocyte activation | 80/7188 | 0.000478347 |  | |
| GO:0008361 | regulation of cell size | 34/7188 | 0.000484476 |  | |
| GO:0040008 | regulation of growth | 40/7188 | 0.000484476 |  | |
| GO:0007167 | enzyme-linked receptor protein signaling pathway | 186/7188 | 0.000484476 | |  |
| GO:0035295 | tube development | 64/7188 | 0.000484476 |  | |
| GO:0098657 | import into cell | 191/7188 | 0.000484476 | |  |
| GO:0006954 | inflammatory response | 90/7188 | 0.000505513 |  | |
| GO:0045321 | leukocyte activation | 93/7188 | 0.000566567 |  | |
| GO:0032101 | regulation of response to external stimulus | 113/7188 | 0.000566567 | |  |
| GO:0030155 | regulation of cell adhesion | 62/7188 | 0.000605164 |  | |
| GO:0061387 | regulation of extent of cell growth | 25/7188 | 0.000605164 |  | |
| GO:1903900 | regulation of viral life cycle | 25/7188 | 0.000605164 |  | |
| GO:0030308 | negative regulation of cell growth | 23/7188 | 0.000630403 |  | |
| GO:0098655 | monoatomic cation transmembrane transport | 228/7188 | 0.000630403 | |  |
| GO:0050768 | negative regulation of neurogenesis | 21/7188 | 0.000630403 |  | |
| GO:0051961 | negative regulation of nervous system development | 21/7188 | 0.000630403 |  | |
| GO:0034220 | monoatomic ion transmembrane transport | 260/7188 | 0.000630403 | |  |
| GO:0032944 | regulation of mononuclear cell proliferation | 33/7188 | 0.00064824 |  | |
| GO:0050670 | regulation of lymphocyte proliferation | 33/7188 | 0.00064824 |  | |
| GO:0070663 | regulation of leukocyte proliferation | 33/7188 | 0.00064824 |  | |
| GO:0048870 | cell motility | 231/7188 | 0.000703822 | |  |
| GO:0031175 | neuron projection development | 163/7188 | 0.000720841 | |  |
| GO:0030042 | actin filament depolymerization | 26/7188 | 0.000828046 |  | |
| GO:0030041 | actin filament polymerization | 57/7188 | 0.000831033 |  | |
| GO:0022407 | regulation of cell-cell adhesion | 41/7188 | 0.000904688 |  | |
| GO:0031345 | negative regulation of cell projection organization | 24/7188 | 0.000913672 |  | |
| GO:0048675 | axon extension | 24/7188 | 0.000913672 |  | |
| GO:0048525 | negative regulation of viral process | 18/7188 | 0.000980407 |  | |
| GO:0010977 | negative regulation of neuron projection development | 22/7188 | 0.000980407 |  | |
| GO:0030516 | regulation of axon extension | 22/7188 | 0.000980407 |  | |
| GO:0050771 | negative regulation of axonogenesis | 20/7188 | 0.001012288 |  | |
| GO:0007159 | leukocyte cell-cell adhesion | 39/7188 | 0.001100967 |  | |
| GO:0040007 | growth | 51/7188 | 0.001100967 |  | |
| GO:0048589 | developmental growth | 43/7188 | 0.001151526 |  | |
| GO:0016049 | cell growth | 36/7188 | 0.001166944 |  | |
| GO:0050793 | regulation of developmental process | 210/7188 | 0.001205215 | |  |
| GO:0140888 | interferon-mediated signaling pathway | 15/7188 | 0.001466291 |  | |
| GO:0001667 | ameboidal-type cell migration | 28/7188 | 0.001470469 |  | |
| GO:0120039 | plasma membrane bounded cell projection morphogenesis | 117/7188 | 0.001470469 | |  |
| GO:0048858 | cell projection morphogenesis | 119/7188 | 0.001500439 | |  |
| GO:0048667 | cell morphogenesis involved in neuron differentiation | 107/7188 | 0.00162842 | |  |
| GO:0051336 | regulation of hydrolase activity | 128/7188 | 0.001647444 | |  |
| GO:0030001 | metal ion transport | 243/7188 | 0.00164779 | |  |
| GO:0050792 | regulation of viral process | 26/7188 | 0.00173349 |  | |
| GO:0060284 | regulation of cell development | 71/7188 | 0.001906396 |  | |
| GO:0030335 | positive regulation of cell migration | 53/7188 | 0.002010777 |  | |
| GO:1990138 | neuron projection extension | 27/7188 | 0.002345742 |  | |
| GO:0034340 | response to type I interferon | 14/7188 | 0.002739999 |  | |
| GO:0048812 | neuron projection morphogenesis | 114/7188 | 0.003003812 | |  |
| GO:0048588 | developmental cell growth | 28/7188 | 0.003003812 |  | |
| GO:0060560 | developmental growth involved in morphogenesis | 28/7188 | 0.003003812 |  | |

**Supplementary Table S10.** *Relevant Differentially Expressed Genes for Necrosis versus Healthy Uninfected Comparisons.*

| **Gene** | **Change** | **log2 F.C.** | **p.adjusted** |
| --- | --- | --- | --- |
| NTN4 | Decreased | -4.263 | 2.1195E-30 |
| AQP5 | Decreased | -4.263 | 2.1195E-30 |
| SAA3 | Increased | 10.343 | 6.9202E-29 |
| CLDN7 | Decreased | -4.442 | 6.9202E-29 |
| M-SAA3.2 | Increased | 10.841 | 7.064E-26 |
| CLIC5 | Decreased | -5.139 | 4.017E-25 |
| CLDN4 | Decreased | -5.269 | 3.6715E-23 |
| KRT8 | Decreased | -4.499 | 1.1097E-22 |
| KRT19 | Decreased | -4.902 | 1.3274E-22 |
| CTSK | Increased | 6.584 | 3.7475E-22 |
| CXCL17 | Decreased | -4.149 | 8.4206E-21 |
| KRT7 | Decreased | -4.375 | 1.8601E-20 |
| KLF5 | Decreased | -3.192 | 2.5026E-19 |
| AGER | Decreased | -6.164 | 6.5596E-19 |
| CYP4B1 | Decreased | -6.638 | 1.0355E-18 |
| CTSL | Increased | 8.22 | 1.3294E-18 |
| SPP1 | Increased | 10.709 | 1.3429E-18 |
| CLDN5 | Decreased | -6.149 | 8.5965E-18 |
| MMP13 | Increased | 9.178 | 4.6635E-17 |
| COL4A5 | Decreased | -5.235 | 8.0345E-17 |
| OCLN | Decreased | -4.355 | 1.0666E-16 |
| CLDN18 | Decreased | -5.079 | 1.9986E-16 |
| ESAM | Decreased | -3.873 | 2.0832E-16 |
| DKK3 | Decreased | -2.788 | 2.5846E-16 |
| HOPX | Decreased | -3.548 | 4.4242E-16 |
| UGT1A1 | Decreased | -5.017 | 7.2835E-16 |
| SFTPB | Decreased | -4.842 | 1.7623E-15 |
| MMP9 | Increased | 11.494 | 3.664E-15 |
| SFTPD | Decreased | -4.726 | 4.9751E-15 |
| EPCAM | Decreased | -4.063 | 7.8579E-15 |
| SLC6A14 | Decreased | -4.652 | 9.3967E-15 |
| BMP2 | Decreased | -3.394 | 1.672E-14 |
| LAMB2 | Decreased | -4.044 | 1.8756E-14 |
| CD34 | Decreased | -3.827 | 2.1086E-14 |
| SCGB1A1 | Decreased | -5.39 | 2.3592E-14 |
| LAMA3 | Decreased | -3.998 | 2.5126E-14 |
| GATA6 | Decreased | -3.454 | 3.2659E-14 |
| ELN | Decreased | -3.846 | 7.7832E-14 |
| AQP1 | Decreased | -4.474 | 1.0274E-13 |
| FMO3 | Decreased | -4.035 | 1.2015E-13 |
| S1PR1 | Decreased | -3.622 | 1.4383E-13 |
| MMP1 | Increased | 12.415 | 3.0352E-13 |
| CLDN3 | Decreased | -3.875 | 3.72E-13 |
| CDH5 | Decreased | -3.756 | 4.6676E-13 |
| CXCL8 | Increased | 7.374 | 6.0177E-13 |
| ISG15 | Increased | 6.533 | 6.3192E-13 |
| WNT2B | Decreased | -3.63 | 1.1668E-12 |
| COL4A3 | Decreased | -4.97 | 1.2968E-12 |
| CCND1 | Decreased | -3.671 | 1.8891E-12 |
| IFIT2 | Increased | 6.76 | 2.3143E-12 |
| KLF4 | Decreased | -3.367 | 2.962E-12 |
| GJA5 | Decreased | -3.539 | 4.2606E-12 |
| SCGB2A2 | Decreased | -5.616 | 5.1077E-12 |
| FMO5 | Decreased | -2.852 | 7.9782E-12 |
| AURKA | Increased | 2.495 | 9.6536E-12 |
| CLDN1 | Decreased | -2.964 | 1.5365E-11 |
| KRT18 | Decreased | -3.444 | 1.6885E-11 |
| MMP19 | Increased | 3.265 | 1.8441E-11 |
| SLC16A11 | Decreased | -3.224 | 2.7484E-11 |
| CYP2F1 | Decreased | -4.971 | 2.8247E-11 |
| TEK | Decreased | -3.648 | 3.1758E-11 |
| BMP3 | Decreased | -3.739 | 3.4578E-11 |
| IL7R | Increased | 4.994 | 4.1244E-11 |
| NKX2-1 | Decreased | -3.369 | 4.2291E-11 |
| IFIT3 | Increased | 5.614 | 4.6976E-11 |
| CD83 | Decreased | -2.423 | 1.9399E-10 |
| AOX1 | Decreased | -4.322 | 2.5752E-10 |
| SFTPC | Decreased | -5.413 | 3.6475E-10 |
| SFTPA1 | Decreased | -4.579 | 5.0406E-10 |
| ANGPTL6 | Increased | 4.38 | 5.2626E-10 |
| KCNH4 | Increased | 5.845 | 7.1929E-10 |
| CXCL2 | Increased | 6.096 | 1.2665E-09 |
| SCGB1D | Decreased | -6.854 | 3.417E-09 |
| IFT57 | Decreased | -2.17 | 4.1279E-09 |
| CCNB2 | Increased | 2.57 | 4.3638E-09 |
| MMP3 | Increased | 10.168 | 4.4896E-09 |
| SLC6A20 | Decreased | -3.116 | 6.7482E-09 |
| LAMA5 | Decreased | -3.303 | 6.8436E-09 |
| TNFSF15 | Decreased | -3.231 | 7.6277E-09 |
| COL4A2 | Decreased | -3.369 | 8.4221E-09 |
| IFIH1 | Increased | 3.328 | 8.9745E-09 |
| ILDR1 | Decreased | -3.093 | 1.1472E-08 |
| TEAD1 | Decreased | -2.237 | 1.6429E-08 |
| MX2 | Increased | 4.652 | 1.6636E-08 |
| IL20RB | Decreased | -2.529 | 1.7486E-08 |
| COL4A6 | Decreased | -3.627 | 2.736E-08 |
| MCM10 | Increased | 2.656 | 4.1968E-08 |
| MMRN2 | Decreased | -3.121 | 6.6147E-08 |
| PAX2 | Increased | 10.049 | 1.0013E-07 |
| PIGR | Decreased | -3.131 | 1.3026E-07 |
| BMP4 | Decreased | -2.102 | 1.3095E-07 |
| IFI30 | Increased | 2.508 | 1.4138E-07 |
| UGT1A6 | Decreased | -7.259 | 1.5795E-07 |
| IL1B | Increased | 3.445 | 1.7086E-07 |
| SLC22A31 | Decreased | -3.652 | 1.7533E-07 |
| TEAD3 | Decreased | -2.321 | 1.89E-07 |
| MX1 | Increased | 4.007 | 2.2595E-07 |
| IL17F | Increased | 7.041 | 2.7854E-07 |
| FOXJ1 | Decreased | -4.195 | 3.2697E-07 |
| ILDR2 | Decreased | -3.304 | 3.8134E-07 |
| TSLP | Decreased | -3.331 | 4.8064E-07 |
| FBLN5 | Decreased | -2.84 | 4.8109E-07 |
| LAMB3 | Decreased | -2.344 | 5.1236E-07 |
| YAP1 | Decreased | -2.6 | 6.535E-07 |
| FMO2 | Decreased | -3.571 | 7.1728E-07 |
| WNT7A | Decreased | -3.903 | 7.9332E-07 |
| MUC1 | Decreased | -2.777 | 8.146E-07 |
| KCNK9 | Increased | 7.695 | 1.0418E-06 |
| USP18 | Increased | 3.969 | 1.1992E-06 |
| AOX2 | Decreased | -4.374 | 1.5009E-06 |
| FOXA1 | Decreased | -2.745 | 1.7079E-06 |
| CDC6 | Increased | 2.764 | 2.0437E-06 |
| COL4A1 | Decreased | -2.635 | 2.2001E-06 |
| BMP6 | Decreased | -3.171 | 2.5395E-06 |
| WNT9B | Increased | 6.263 | 2.9532E-06 |
| ZBP1 | Increased | 3.894 | 3.0035E-06 |
| RASIP1 | Decreased | -3.145 | 3.0945E-06 |
| CYP2A13 | Decreased | -5.175 | 3.8214E-06 |
| PLK1 | Increased | 2.095 | 3.8473E-06 |
| OAS2 | Increased | 3.439 | 4.0642E-06 |
| OAS1Z | Increased | 3.491 | 6.3223E-06 |
| DKK2 | Decreased | -4.646 | 6.3952E-06 |
| RSAD2 | Increased | 4.027 | 6.8009E-06 |
| IL17A | Increased | 6.368 | 8.2918E-06 |
| OAS1X | Increased | 3.071 | 8.9733E-06 |
| ECM1 | Increased | 2.876 | 1.1939E-05 |
| IRF4 | Increased | 2.973 | 1.2838E-05 |
| MMP25 | Increased | 3.58 | 1.4884E-05 |
| MUC15 | Decreased | -3.256 | 1.5784E-05 |
| NOTCH3 | Decreased | -2.616 | 1.594E-05 |
| ITIH5 | Decreased | -3.196 | 1.6088E-05 |
| HMCN1 | Decreased | -3.15 | 1.7653E-05 |
| IL21R | Increased | 3.209 | 1.8073E-05 |
| BIRC5 | Increased | 2.773 | 1.9409E-05 |
| SPON2 | Decreased | -3.198 | 1.9524E-05 |
| IL21 | Increased | 4.65 | 2.195E-05 |
| AURKB | Increased | 2.459 | 2.3075E-05 |
| IFI44L | Increased | 2.962 | 2.4047E-05 |
| DNAL1 | Decreased | -1.791 | 2.5059E-05 |
| UGT2A1 | Decreased | -4.653 | 2.8272E-05 |
| TIE1 | Decreased | -2.44 | 2.8292E-05 |
| OAS1Y | Increased | 3.049 | 3.6003E-05 |
| TEAD4 | Decreased | -2.268 | 3.7293E-05 |
| IFI6 | Increased | 3.003 | 3.8981E-05 |
| IFI44 | Increased | 2.765 | 3.8991E-05 |
| MMP12 | Increased | 5.03 | 4.4509E-05 |
| PAX1 | Increased | 8.637 | 4.9081E-05 |
| PRELP | Decreased | -3.114 | 7.045E-05 |
| CCNB1 | Increased | 2.748 | 7.5391E-05 |
| IRF1 | Increased | 2.089 | 8.4014E-05 |
| CXCL5 | Increased | 3.06 | 9.0775E-05 |
| TNF | Decreased | -3.114 | 9.3236E-05 |
| IL17RB | Decreased | -2.822 | 0.00010792 |
| IRF7 | Increased | 2.99 | 0.00011183 |
| DNAH11 | Decreased | -2.77 | 0.00014858 |
| CDC20 | Increased | 2.293 | 0.00016415 |
| CCL8 | Increased | 3.982 | 0.00025613 |
| TEKT3 | Decreased | -2.155 | 0.0002678 |
| TEKT1 | Decreased | -2.777 | 0.00026959 |
| RSPO4 | Increased | 6.311 | 0.0003113 |
| CNTNAP2 | Increased | 3.394 | 0.00036541 |
| MXRA5 | Increased | 2.918 | 0.00045964 |
| BMP1 | Increased | 2.014 | 0.0005029 |
| SCGB3A2 | Decreased | -3.131 | 0.00057163 |
| KIF20A | Increased | 2.236 | 0.00057713 |
| PAX6 | Increased | 4.456 | 0.0006413 |
| CDHR2 | Increased | 8.482 | 0.00070357 |
| COL22A1 | Increased | 6.476 | 0.0008072 |
| IL24 | Increased | 2.595 | 0.00092294 |
| HOXD10 | Increased | 5.234 | 0.00095554 |
| MMP17 | Increased | 4.925 | 0.00103251 |
| SLC45A1 | Decreased | -3.542 | 0.00111048 |
| GDF15 | Increased | 3.664 | 0.0011407 |
| PLVAP | Decreased | -2.717 | 0.00115274 |
| CFAP47 | Decreased | -2.703 | 0.00116123 |
| CDH18 | Increased | 8.461 | 0.00116329 |
| DLX4 | Increased | 3.492 | 0.00120019 |
| CD209 | Decreased | -2.388 | 0.00127577 |
| POU3F1 | Increased | 4.362 | 0.0012807 |
| JAG2 | Decreased | -2.704 | 0.00143543 |
| PCDH15 | Increased | 6.257 | 0.00143881 |
| KIF18B | Increased | 2.23 | 0.00165451 |
| IL23R | Increased | 2.501 | 0.00178153 |
| POU3F3 | Increased | 8.847 | 0.0018428 |
| CNTN2 | Increased | 3.928 | 0.00213671 |
| DNAL4 | Decreased | -1.459 | 0.00220723 |
| PCDH8 | Increased | 6.457 | 0.00258589 |
| WNT6 | Increased | 6.436 | 0.00267812 |
| KLRK1 | Decreased | -2.012 | 0.0027162 |
| IL22 | Increased | 6.075 | 0.00274767 |
| DNAH5 | Decreased | -2.75 | 0.00327775 |
| DLX3 | Increased | 3.51 | 0.00345244 |
| PAX3 | Increased | 7.165 | 0.00356607 |
| CYP2B6 | Decreased | -3.087 | 0.00423988 |
| CYP1A1 | Decreased | -3.976 | 0.00441268 |
| CCL3 | Increased | 2.472 | 0.00629054 |
| CFAP43 | Decreased | -2.175 | 0.00670925 |
| WNT11 | Decreased | -2.302 | 0.00748132 |
| COL23A1 | Increased | 2.386 | 0.00841791 |
| COL19A1 | Increased | 3.558 | 0.0090981 |
| CFAP299 | Decreased | -2.282 | 0.01066218 |
| HOXA10 | Increased | 7.939 | 0.01114039 |
| IL1A | Increased | 2.154 | 0.01247701 |
| CFAP52 | Decreased | -2.013 | 0.01304591 |
| BARX1 | Increased | 4.465 | 0.01305502 |
| HOXC8 | Increased | 6.993 | 0.01340792 |
| POU3F2 | Increased | 5.722 | 0.01486684 |
| IL26 | Increased | 3.076 | 0.01598864 |
| HOXA11 | Increased | 7.463 | 0.01691149 |
| CDH9 | Unchanged | 6.921 | 0.0187118 |
| DLX5 | Increased | 4.785 | 0.02327351 |
| DLX1 | Increased | 7.714 | 0.0278347 |
| WNT10A | Increased | 3.609 | 0.02796113 |
| HOXD13 | Increased | 7.568 | 0.0280044 |
